# The intrarenal landscape of T cell receptor repertoire in clear cell renal cell cancer

**DOI:** 10.1186/s12967-022-03771-3

**Published:** 2022-12-03

**Authors:** Wei Zhang, Qian Zhang, Chao Zhu, Zhiyuan Shi, Chen Shao, Yujie Chen, Nan Wang, Yanxia Jiang, Qing Liang, Kejia Wang

**Affiliations:** 1 Fujian Provincial Key Laboratory of Organ and Tissue Regeneration, Xiamen Key Laboratory of Regeneration Medicine, Organ Transplantation Institute of Xiamen University, School of Medicine, Xiamen University, Xiamen, China; 2 Department of Pathology, The 971 Hospital of People’s Liberation Army Navy, Qingdao, China; 3 Department of Nephrology, Changhai Hospital, Second Military Medical University, Shanghai, China; 4 Department of Urology, Xiang’an Hospital of Xiamen University, School of Medicine, Xiamen University, Xiamen, China; 5 Department of Pathology, The Affiliated Hospital of Qingdao University, Qingdao, China

**Keywords:** Clear cell renal cell cancer, Immune repertoire, T-cell receptor, T-cell infiltration

## Abstract

**Background:**

Clear cell renal cell cancer (ccRCC) is accompanied by T-cell infiltration. In this study, we sought to determine the difference in T-cell infiltration and the T-cell receptor (TCR) immune repertoire between ccRCC and peritumour tissue.

**Methods:**

T-cell infiltration was examined using immunohistochemistry (IHC) and haematoxylin and eosin (HE) staining. The chi-squared test and Pearson correlation analysis were applied to evaluate the relationship between clinical traits and CD3, CD4, and CD8 expression. Immune repertoire sequencing (IR-Seq) was used to describe the profile of the TCR repertoire.

**Results:**

The adjacent tissue showed increased expression of CD3, CD4 and CD8 compared with ccRCC tissue (*P*_*CD3*_ = 0.033; *P*_*CD4*_ = 0.014; *P*_*CD8*_ = 0.004). Indicated CD3^+^ T-cell density in ccRCC tissue was positively correlated with that in peritumour tissue (*P* = 0.010, r = 0.514), which implied the T cells in peritumour tissue directly infect the number of cells infiltrating in ccRCC tissue. Moreover, there was a positive correlation between Vimentin expression and indicated positive T-cell marker in ccRCC tissue (*P*_*CD3*_ = 0.035; *P*_*CD4*_ = 0.020; *P*_*CD8*_ = 0.027). Advanced stage revealed less CD4^+^ T-cell infiltration in ccRCC tissue (*P*_*CD4*_ = 0.023). The results from IR-Seq revealed an obvious increase in VJ and VDJ segment usage, as well as higher complementarity-determining region 3 (CDR3) amino acid (aa) clonotypes in ccRCC. The matched antigen recognized by the TCR of ccRCC may be potential targets.

**Conclusions:**

The current study collectively demonstrates diminished T-cell infiltration and increased CDR3 aa diversity in ccRCC, which may be associated with immunotherapeutic targets for ccRCC patients.

**Supplementary Information:**

The online version contains supplementary material available at 10.1186/s12967-022-03771-3.

## Introduction

Clear cell renal carcinoma (ccRCC) is a highly aggressive renal malignant tumour, with an estimated 2.3% of global cancer deaths in 2022 [1]. Although advanced imaging equipment improves ccRCC diagnosis, nearly 30% of patients are diagnosed at an advanced stage due to no obvious symptoms [2]. Half of the patients with ccRCC eventually develop metastases [3, 4]. Moreover, all treatment of metastatic ccRCC patients is restricted to similar surgery, chemotherapy, radiotherapy and drugs, and its 5-year survival rate is under 10% [5]. Therefore, to prolong patient survival time, since 2015, tumour-infiltrating lymphocyte (TIL) adoptive therapy has been applied in ccRCC patients and has shown modest success [6]. However, the function of these TILs found in ccRCC is affected by the tumour microenvironment (TME), which is often impaired and incompletely activated or anergic. The study of Ulla Kring Hansen et al*.* confirmed this opinion [7]; They found that the immune responses of expanded CD8^+^ ccRCC-TILs were typically weaker (2.20%) and displayed a mono/oligo functional pattern. A better understanding of the interplay between TILs and tumours is required to develop more effective therapies for ccRCC.

In recent years, several studies have confirmed that ccRCC is accompanied by immune cell infiltration [8–10]. The ccRCC-associated TME provides potential targets for immunotherapy. Determining the features of TILs in ccRCC in detail could improve therapy. TIL is the capacity of specific neoantigen recognition. Therefore, T-cell receptor (TCR) features have become a major subject to study [11, 12]. The majority of TCRs are composed of α chains and β chains, and each chain has a constant and variable domain. The variable domain is encoded by variable (V), diversity (D) and connected (J) segments. Its recombination randomly causes TCR diversity, which is closely associated with the host immune response and cancer prognosis. TCR repertoires play a pivotal role in the development of effective immunotherapy, and the preferential accumulation of proinflammatory immune cells is associated with longer survival in patients suffering from ccRCC [13, 14]. However, the characteristics of the TCR repertoire in ccRCC are less clear, and its profile of proinflammatory immune cells has not been confirmed. Therefore, it is important to identify TCR specificity, which may lead to further identification of possible therapeutic strategies for ccRCC (Additional files 1, 2).

Here, we measured the infiltration of CD3^+^, CD4^+^, and CD8^+^ cells in ccRCC and peritumour tissue and analysed the correlation between clinical traits and T-cell expression. Furthermore, we detected TCR genomic features to identify potential immunotherapeutic targets in ccRCC (Additional files 3, 4, 5).

## Methods

### Patients

A total of 44 matched ccRCC and adjacent normal renal tissues were obtained from The 971 Hospital of People’s Liberation Army Navy and The Affiliated Hospital of Qingdao University between 2019 and 2021. A total of 25 items were obtained from the patients' medical records, including patients’ information and clinical features. The pathologic diagnosis was confirmed independently by 3 pathologists with extensive clinical experience. The present study was approved by the Ethics Committee of School of Medicine, Xiamen University. All patients in this study provided written informed consent for their participation.

### Immunohistochemical (IHC) staining

All specimens were fixed in 4% paraformaldehyde at 4 °C for 48 h and serially sectioned into 5 μm-thick sections. Part of the sections was stained with haematoxylin for 5 min and eosin for 2 min. The other part of the section was used for IHC staining. Immunohistochemical staining was performed using CD3 (Cat: Kit-0003, Clone: SP7, Ready-to-use, MXB Biotechnologies), CD4 (Cat: RMA-0620, Clone: SP35, Ready-to-use, MXB Biotechnologies), CD8 (Cat: RMA-0514, Clone: SP16, Ready-to-use, MXB Biotechnologies), CK7 (Cat: ZA-0573, Clone: EP16, 1:200, ZSGB-BIO), CD10 (Cat: 05857856001, Clone: SP67, Ready-to-use, Ventana), CD117 (Cat: ZA-0523, Clone: EP10, 1:60, ZSGB-BIO), Vimentin (Cat: 05278139001, Clone: V9, Ready-to-use, Ventana), E-Cadherin (Cat: MAB-0738, Clone: MX020, Ready-to-use, MXB Biotechnologies), carbonic anhydrase (CA-IX) (Cat: RAB-0615, Clone: RAB-0615, Ready-to-use, MXB Biotechnologies), Pax-8 (Cat: ab191870, Clone: EPR18715, 1:100, Abcam), P540S (Cat: RMA-0546, Clone: 13H4, 1:80, MXB Biotechnologies), transcription Factor E3 (TFE3) (Cat: ab179804, Clone: EPR11591, 1:400, Abcam) and Ki67 (Cat: 05278384001, Clone: 30-9, Ready-to-use, Ventana) primary antibodies at 4 °C overnight. Then, the cells were stained with secondary antibody for 1 h at room temperature using OptiView DAB IHC Detection Kit (Cat: 06396500001, Ready-to-use, Ventana). The brown signals located in the plasma membrane represented positive staining for the respective proteins. Normal renal cortex treated with monoclonal antibodies was used as negative control (Additional file 6: Fig. S1). The expression of CD3, CD4, CD8, CK7, CD10, CD117, Vimentin, E-Cadherin, CA-IX, Pax-8, P540S, TFE3, and Ki67 was assessed independently based on the proportion of positive cells by two pathologists who were blinded to the clinical data. The positive staining value of peritumour and tumour tissue was assessed by the positive staining density using ImageJ (V1.53, National Institutes of Health). The final positive value was obtained by averaging the positive value in three different visual fields. All specimens were tested in parallel for the entire set.

### Flow cytometry

Peritumour tissue and ccRCC tissue from ccRCC patients were individually dissociated in 50 U/mml of Dispase II (S25046, YuanyeBio-Technology, China) on gentleMACS octo with Heater (Miltenyi Biotec, German) to enable efficient dissociation of lymphocytes from the tumour cells and stromal tissue. Staining steps were performed at 4 ℃ over 20 min with FACS buffer washes between steps. Flow cytometry antibodies were used: FITC-CD3 (Cat: 317305, Clone: OKT3, 1:20, Biolegend), PE-IFN-γ (Cat: 502508, Clone: 4SB3, 1:20, Biolegend), APC-PD-1 (Cat: 379207, Clone: A17188A, 1:20, Biolegend).

### TCR sequence analysis

For total RNA extraction, fresh tissue lysates were first prepared by using sterile steel balls and TRIzol. This was followed by the addition of chloroform to dissolve the total RNA. The RNA was precipitated by adding isopropyl alcohol equal to the volume of aqueous solution. Then, 200 ng of total RNA was reverse-transcribed into cDNA using a Transcriptor First Strand cDNA Synthesis Kit (LABLEAD, Beijing, China) on a C1000 TouchTM Thermal Cycler (Bio-Rad Inc., Hercules, CA, United States). Two-round nested amplicon arm-PCR with specific primers was performed using 2 × Taq master Mix (Vazyme, Nanjing, China) as previously described [15]. Amplicons were extracted from 1.5% agarose gels and purified using the AxyPrep DNA Gel Extraction Kit (Hlingene, Shanghai, China). Purified amplicons were paired-end sequenced (PE250) on the Illumina platform according to standard protocols. The sequencing data were stored in FASTQ format. First, the low-quality sequences were filtered out, and the remaining sequences were reserved for further analysis. BLAT software was used to find TCR V, D, and J genes in each sequence in the TCR reference genome downloaded from the International Immunogenetics Information System (IMGT)/GeneDB database. Sequences containing V, D, and J gene segments were extracted and further translated into CDR3 aa sequences. Finally, the ggseqlogo 0.1 package was used to identify the motif of CDR3. The VDJmatch 1.2.2, VDJtools 1.2.1 package, and VDJdb (2022) [16] were used to identify the antigen and diversify clonotypes of Vα-CDR3-Jα and Vβ-CDR3-Jβ combinations. Other data analyses were performed as previously described [17].

### Statistical analysis

GraphPad Prism 9.0 (GraphPad Software, La Jolla, CA) and SPSS 26.0 software (IBM Corporation, Armonk, NY) were used to plot data and calculate statistics. The positive CD3, CD4 and CD8 value of all ccRCC patients has been divided into two parts (low and high expression level) by the median spacing of the number of positive indicated positive T cells marker. The chi-squared test (χ^2^) is used to evaluate the association between clinical parameters and the number of positive indicated positive T cells marker (CD3, CD4, and CD8) in ccRCC patients (Table 1); When n < 5, we used Fisher's exact test instead of chi-squared test. The correlation of CD3^+^, CD4^+^, and CD8^+^ T cell density between ccRCC and the peritumour group is assessed by using the Pearson correlation analysis on SPSS software (Table 2). The CD3^+^, CD4^+^, and CD8^+^ T cell mean density used in Table 2 are obtained through ImageJ we mentioned before (Method, IHC staining part). Student's t‐test was used to compare distinct genes in ccRCC and peritumour groups, and two groups were considered significantly different when *P* < 0.05. Volcano plots were used to plot the distinct genes according to the *P* value.

**Table 1:** The correlation between clinical parameters and indicated positive T cell marker in ccRCC patients (n = 44)

		CD3			CD4			CD8		
		Low expression	High expression	χ2 / *P*	Low expression	High expression	χ2 / *P*	Low expression	High expression	χ2 / *P*
Tumour size										
< 5 cm	18 (40.90%)	9 (20.45%)	9 (20.45%)	0.063/0.802	9 (20.45%)	9 (20.45%)	0.254/0.614	8 (18.18%)	10 (22.73%)	0.376/0.540
≥ 5 cm	26 (59.09%)	14 (31.82%)	12 (27.27%)		15 (34.09)	11 (25.00%)		14 (31.82%)	12 (27.27%)	
ISUP grade										
Grade 1	6 (13.64%)	3 (6.82%)	3 (6.82%)	1.714/0.634	-	6 (13.64%)	**9.545/0.023**	1 (2.27%)	5 (11.36%)	5.918/0.116
Grade 2	21 (47.73%)	12 (27.27%)	9 (20.45%)		11 (25.00%)	10 (22.73%)		13 (29.55%)	8 (18.18%)	
Grade 3	14 (31.82%)	6 (13.64%)	8 (18.18%)		10 (22.73%)	4 (9.09%)		5 (11.36%)	9 (20.45%)	
Grade 4	1 (2.27%)	-	1 (2.27%)		1 (2.27%)	-		1 (2.27%)	-	
CK7										
Absent	41 (93.18%)	21 (47.73%)	20 (45.45%)	0.931/0.335	22 (50.00%)	19 (43.18%)	0.846/0.358	21 (47.73%)	20 (45.45%)	0.931/0.335
Present	1 (2.27%)	1 (2.27%)	-		1 (2.27%)	-		1 (2.27%)	-	
CD10										
Absent	1 (2.27%)	1 (2.27%)	-	0.885/0.347	1 (2.27%)	-	0.802/0.370	1 (2.27%)	-	0.885/0.347
Present	40 (90.91%)	21 (47.73%)	19 (43.18%)		22 (50.00%)	18 (40.91%)		21 (47.73%)	19 (43.18%)	
CD117										
Absent	35 (79.55%)	18 (40.91%)	17 (38.64%)	0.129/0.720	20 (45.45%)	15 (34.09)	0.015/0.904	19 (43.18%)	16 (36.36%)	0.058/0.810
Present	5 (11.36%)	3 (6.82%)	2 (4.55%)		3 (6.82%)	2 (4.55%)		3 (6.82%)	2 (4.55%)	
Vimentin										
Absent	5 (11.36%)	5 (11.36%)	-	**4.432/0.035**	5 (11.36%)	-	**5.449/0.020**	5 (11.36%)	-	**4.916/0.027**
Present	34 (77.27%)	17 (38.64%)	17 (38.64%)		15 (34.09)	19 (43.18%)		16 (36.36%)	18 (40.91%)	
E-Cadherin										
Absent	5 (11.36%)	2 (4.55%)	1 (2.27%)	0.257/0.612	1 (2.27%)	2 (4.55%)	1.008/0.315	2 (4.55%)	1 (2.27%)	0.103/0.748
Present	35 (79.55%)	18 (40.91%)	17 (38.64%)		22 (50.00%)	13 (29.55%)		20 (45.45%)	15 (34.09)	
CA-IX										
Absent	1 (2.27%)	1 (2.27%)	-	0.924/0.336	1 (2.27%)	-	0.831/0.362	1 (2.27%)	-	1.027/0.311
Present	37 (84.09%)	19 (43.18%)	18 (40.91%)		20 (45.45%)	17 (38.64%)		18 (40.91%)	19 (43.18%)	
Pax-8										
Absent	3 (6.82%)	3 (6.82%)	-	2.265/0.132	2 (4.55%)	1 (2.27%)	0.146/0.702	1 (2.27%)	2 (4.55%)	0.521/0.471
Present	29 (65.91%)	16 (36.36%)	13 (29.55%)		16 (36.36%)	13 (29.55%)		16 (36.36%)	13 (29.55%)	
P504S										
Absent	9 (20.45%)	3 (6.82%)	6 (13.64%)	2.918/0.088	5 (11.36%)	4 (9.09%)	0/0.984	4 (9.09%)	5 (11.36%)	0.558/0.455
Present	29 (65.91%)	19 (43.18%)	10 (22.73%)		16 (36.36%)	13 (29.55%)		17 (38.64%)	12 (27.27%)	
TFE3										
Absent	32 (72.73%)	17 (38.64%)	15 (34.09)	0.020/0.888	15 (34.09)	17 (38.64%)	0.792/0.374	17 (38.64%)	15 (34.09)	0.792/0.374
Present	6 (13.64%)	3 (6.82%)	3 (6.82%)		4 (9.09%)	2 (4.55%)		2 (4.55%)	4 (9.09%)	
Ki-67										
< 10%	16 (36.36%)	11 (25.00%)	5 (11.36%)	3.303/0.192	9	7 (15.91%)	1.971/0.373	9 (20.45%)	7 (15.91%)	2.076/0.354
10–30%	12 (27.27%)	6 (13.64%)	6 (13.64%)		7 (15.91%)	5 (11.36%)		9 (20.45%)	3 (6.82%)	
> 30%	7 (15.91%)	2 (4.55%)	5 (11.36%)		6 (13.64%)	1 (2.27%)		3 (6.82%)	4 (9.09%)	

Bold values represent *P*<0.05

The expression levels of CD3 are divided into low and high expression level by the median spacing of the number of positive indicated positive T cells marker

*ccRCC* clear cell renal cell cancer, *ISUP* Internal Society of Urologic Pathology, *CA-IX* carbonic anhydrase, *TFE3* transcription factor E3

**Table 2:** Correlation analysis of CD3, CD4, and CD8 T cell density in ccRCC and peritumour tissue

		ccRCC		
	*P* value/CC	CD3	CD4	CD8
Peritumour	CD3	**0.010/ 0.514**	0.933/ 0.018	0.920/ 0.022
	CD4	0.643/ 0.097	0.573/ 0.118	0.692/− 0.083
	CD8	0.157/ 0.292	0.061/ − 0.380	0.127/ − 0.314

Bold values represent *P*<0.05

*ccRCC* clear cell renal cell cancer, *CC* correlation coefficient

## Results

### Clinical characteristics

The clinical characteristics of 44 ccRCC patients are listed in Table 1. Among all subjects, the mean age was 58 years. Over two-thirds (68.18%) of the patients were males, and over half of them (59.09%) had tumours larger than 5 cm in diameter. According to the International Society of Urological Pathology (ISUP) grading system, there were 6 patients (13.64%) in stage I, 21 patients (47.73%) in stage II, 14 patients (31.82%) in stage III and 1 patient (2.27%) in stage IV. The majority of patients were negative for CK7 (93.18%), CD117 (79.55%) and TFE3 (13.64%). A total of 40 patients (90.91%) were positive for CD10, 34 patients (77.27%) were positive for Vimentin, 35 patients (79.55%) were positive for E-cadherin, 37 patients (84.09%) were positive for CA IX and 29 patients (65.91%) were positive for Pax-8 and P504S. Ki-67 was highly expressed (> 30.00%) in 7 (15.91%) patients and slightly expressed (0–10.00%) in 16 (36.36%) patients.

### Fewer T-cell infiltration in ccRCC tissues

**Fig. 1:**
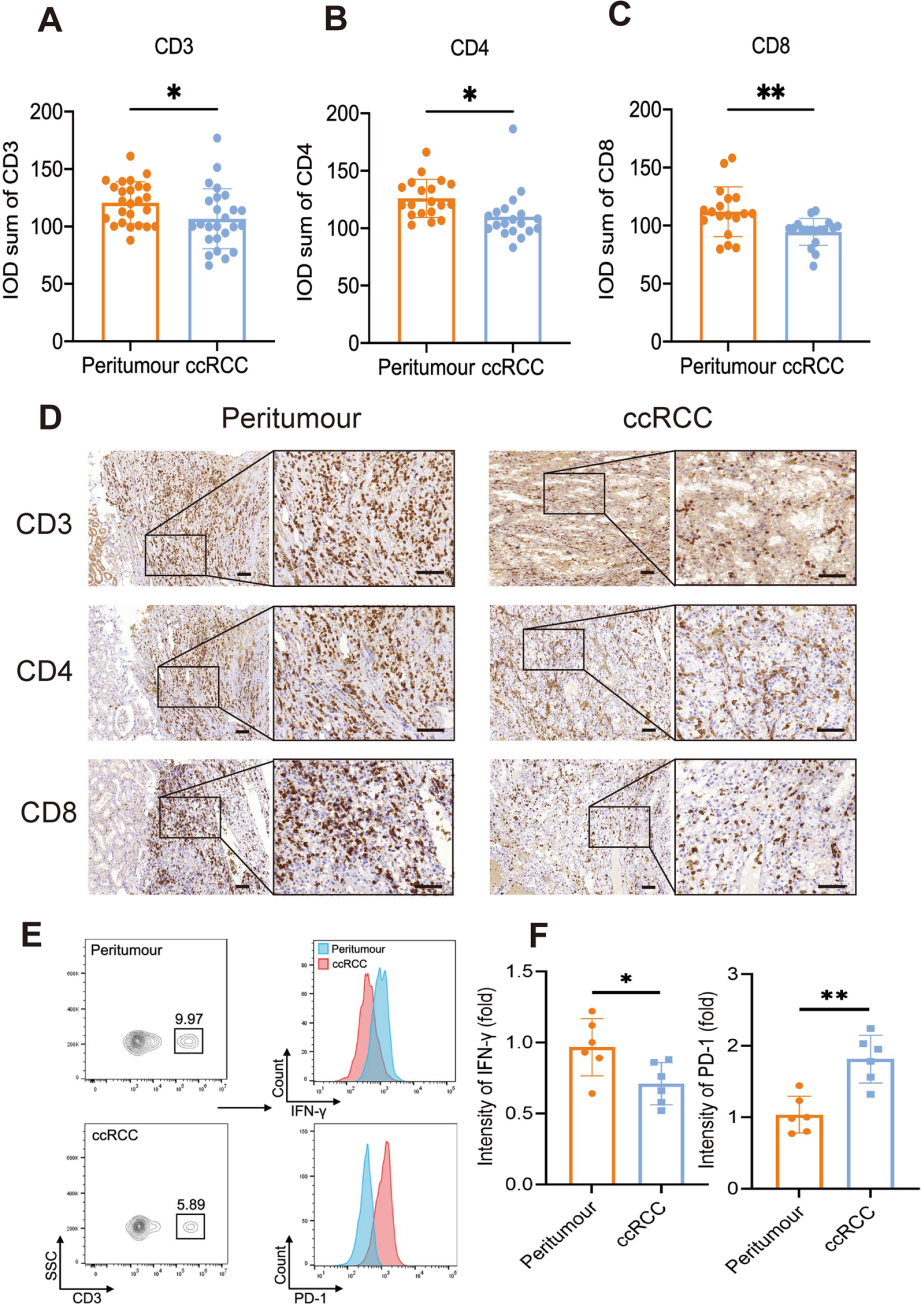
T-cell infiltration reduction in ccRCC than in peritumour tissue. **A**, **B**, **C** Statistical analysis of CD3, CD4, and CD8 immunohistochemistry results in ccRCC and peritumour tissue (n = 44, *P* < 0.05). **D** Representative images of CD3, CD4, and CD8 immunohistochemistry results in peritumour (Left) and ccRCC (Right) tissue. Scale bar, 100 μm. **E** Representative images of CD3, PD-1 expression and IFN-γ secretion by flow cytometry in peritumour and ccRCC tissue. **F** Statistical analysis of flow cytometry results of IFN-γ secretion (Left) and PD-1 expression (Right). Data are mean ± SEM. Student’s t-test is used to compare the immunohistochemistry results’ difference, IFN-γ secretion, or PD-1 expression between the peritumour and ccRCC group. *P value < 0.05; **P value < 0.01; ***P value < 0.001; *ns* not significant

The correlation between clinical characteristics (including tumour size, Vimentin, Ki67, and E-cadherin) and the indicated positive T cells marker in ccRCC patients are shown in Table 1. There was no significant correlation between clinical characteristics and CD3^+^, CD4^+^ and CD8^+^ T-cell infiltration in ccRCC tissue, except for Vimentin (*P*_CD3_ = 0.035, χ^2^ = 4.432), (*P*_CD4_ = 0.020, χ^2^ = 5.449), (*P*_CD8_ = 0.027, χ^2^ = 4.916) and ISUP grade (*P*_CD4_ = 0.023, χ^2^ = 9.545). Compared to adjacent tissue, CD3^+^, CD4^+^ and CD8^+^ T cells were reduced in ccRCC tissue (*P*_*CD3*_ = 0.033; *P*_*CD4*_ = 0.014; *P*_*CD8*_ = 0.004, Fig. 1A–C). IHC (Fig. 1D) and HE staining (Additional file 7: Fig. S2) showed higher T-cell infiltration in adjacent tissue than in ccRCC tissue. The flow cytometry image also got similar results (Fig. 1E). Compared to peritumour tissue, CD3^+^ (9.97% *vs.* 5.89%) expression decreased in the ccRCC group. Besides, ccRCC tissue showed a higher PD-1 expression (*P* < 0.01) and lower IFN-γ secretion than in peritumour tissue (*P* < 0.05) (Fig. 1F). We also assessed whether there was a relationship of T-cell expression between the tumour and adjacent tissue. The data indicated that CD3^+^ T cell density in ccRCC is positively correlated with it in peritumour tissues (*P* = 0.010, r = 0.514, Table 2). When peritumour infiltrating T cell number increases, T cell number in ccRCC tissue should be theoretically increased. Indeed, the IHC results showed fewer T cells infiltrated in ccRCC, which implied the recruitment of lymphocytes to the site of the tumour seems to be inhibited to some extent.

### The TCR profile between ccRCC and peritumour tissues

**Fig. 2:**
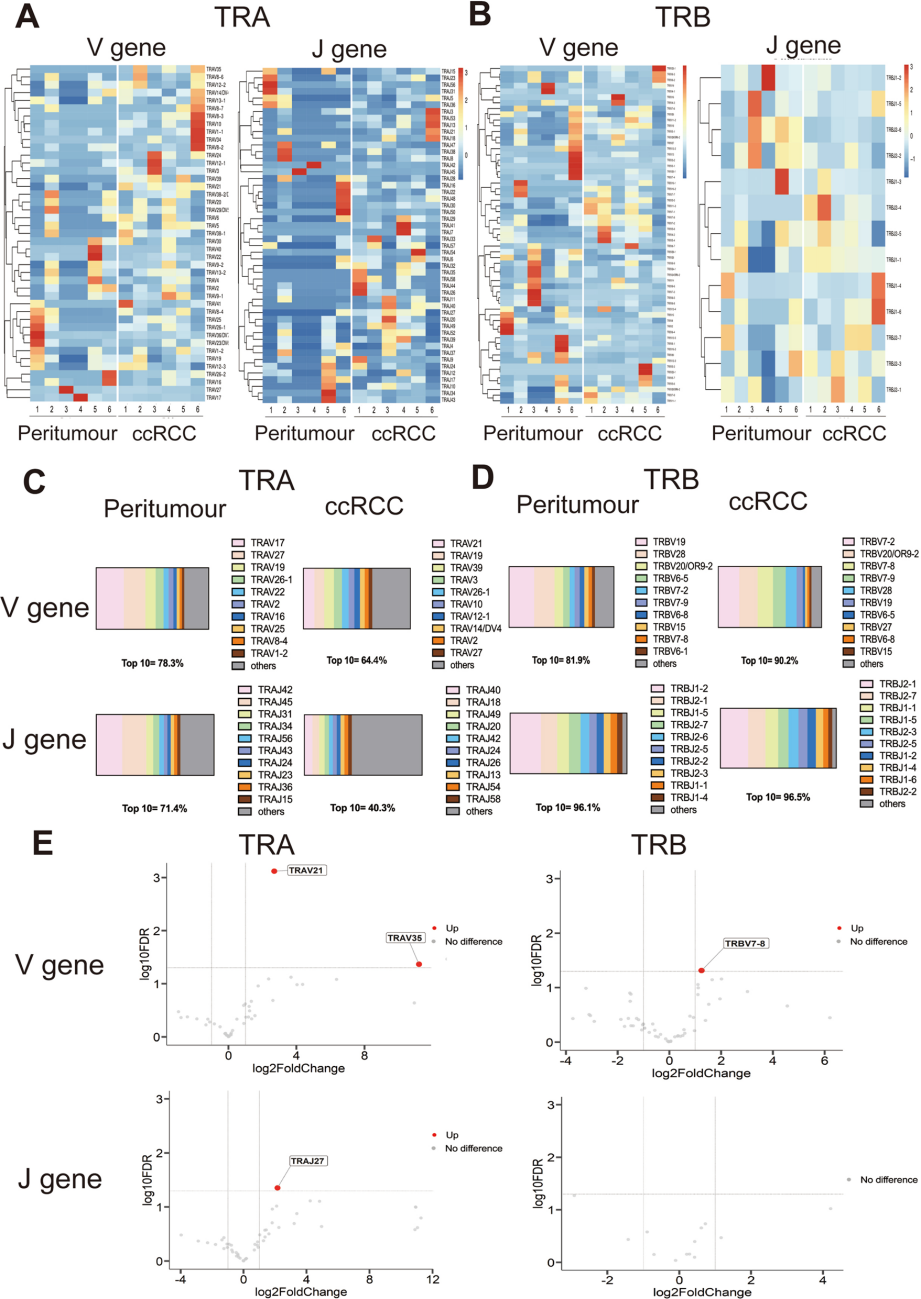
The usage patterns of the V and J genes in ccRCC and peritumour tissue. **A** Heatmaps of V (Left) and J (Right) gene frequencies for TRA in peritumour and ccRCC tissue. **B** Heatmaps of V (Left) and J (Right) gene frequencies for TRB in peritumour and ccRCC tissue. **C**, **D** The V and J gene segments distribution for TRA and TRB in peritumour and ccRCC tissue. **E** Volcano plot showing upregulation (red plot) of the V and J genes in ccRCC compared with peritumour tissue, top 10 upregulation genes had been marked. Student’s t-test is used to compare the distinct gene in the ccRCC group with than peritumour. Red plot: P Value < 0.05; Grey plot: P Value > 0.05

Furthermore, we assessed the variation in the TCR immune repertoire in ccRCC tissue. The heatmap showed obvious differences in the V α/β and J α/β genes in ccRCC and peritumour tissues (Fig. 2A, B). There were also obvious V/J gene usage biases in the two groups. In ccRCC, of all identified TRA segments, TRAV21 (10.20%) and TRAJ40 (6.88%) were the most frequent TRAV/J segments in all patients. The most abundant segments in the TRBV/J genes in the clonotypes were TRBV7-2 (20.21%) and TRBJ2-1 (23.87%). In the peritumour group, among all identified TRAV/J segments, TRAV17 (24.53%) and TRAJ42 (22.02%) were the most frequent segments in peritumour tissue. The most abundant segments in the TRBV/J genes were TRBV19 (23.96%) and TRBJ1-2 (26.34%) (Fig. 2C, D). Full TRAV and TRBV information is in Additional file 1, 2. In addition, TRAV21 and TRAJ27 of the alpha chain and TRBV7-8 of the beta chain had significantly higher expression in ccRCC than in the peritumour group (*P* < 0.05) (Fig. 2E).

**Fig. 3:**
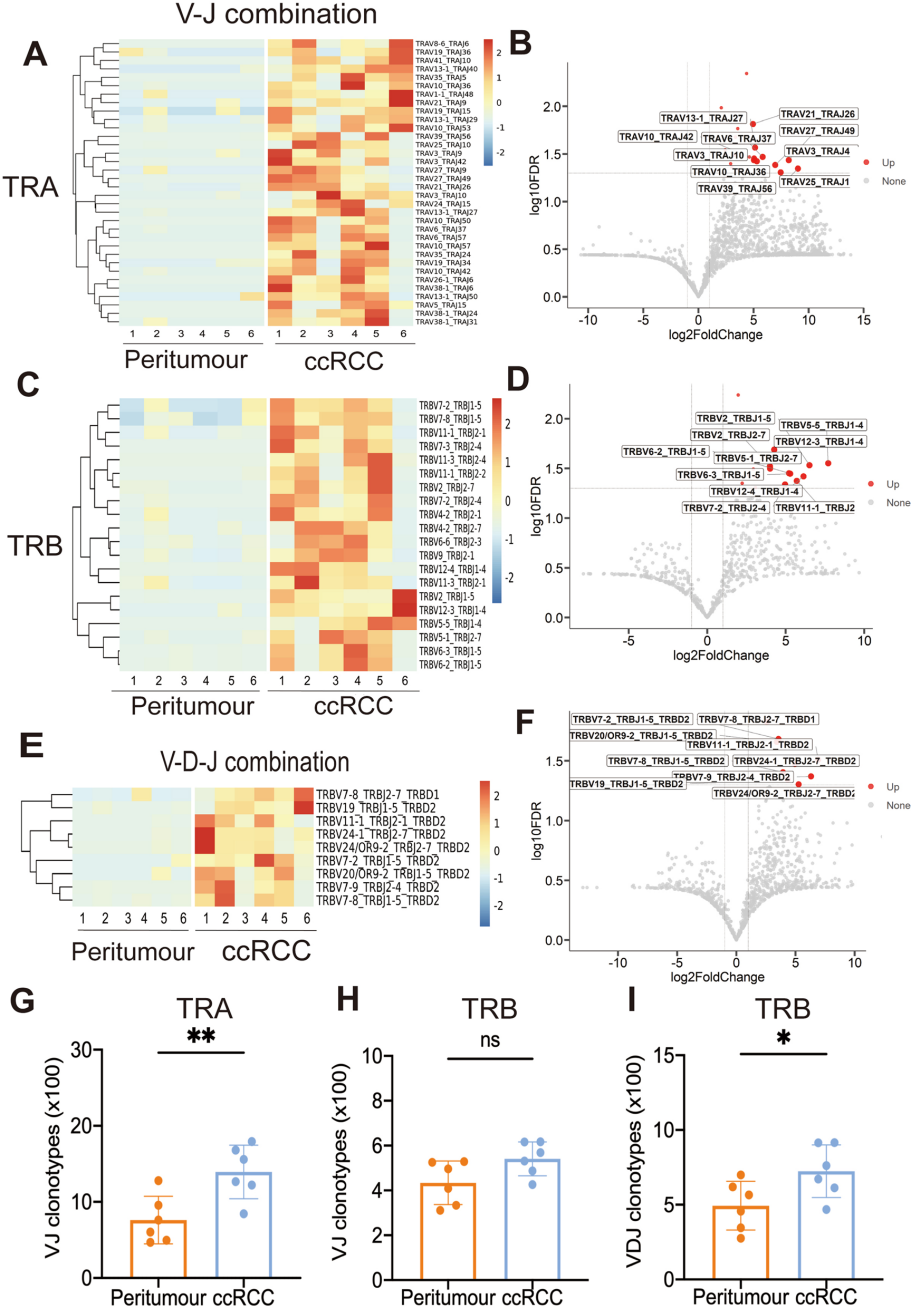
The V-J and V-D-J combination usage patterns for TRA and TRB in ccRCC and peritumour tissue. **A**, **C**, **E** Heatmaps showing the hierarchical clustering of the differentially expressed percentage of V-J for TRA and TRB and V-D-J for TRB in peritumour and ccRCC tissue. **B**, **D**, **F** Volcano plots showing upregulate V-J and V-D-J combinations in ccRCC compared with peritumour tissue (red plots). **G**, **H**, **I** Comparison of V-J clonotypes for TRA and TRB and V-D-J clonotypes for TRB in peritumour and ccRCC tissue. Student’s t-test is used to compare the distinct gene, V-J gene, and V-D-J gene between the peritumour and ccRCC group. Red plot in volcano pot: *P* value < 0.05; Grey plot in volcano pot: *P value < 0.05; **P value < 0.01; ***P value < 0.001; ns, not significant

We also detected the composition of V-J gene combinations and V-D-J combinations. The heatmap revealed that the Vα-Jα, Vβ-Jβ and Vβ-Dβ-Jβ paired genes had distinctive usage patterns between ccRCC and peritumour tissue (Fig. 3A, C, E). Volcano plots were generated to show increased genes in ccRCC compared to peritumour tissue. ccRCC tissue showed 34 distinct Vα-Jα, 20 distinct Vβ-Jβ and 9 distinct Vβ-Dβ-Jβ peptide expression levels (Fig. 3B, D, F). Furthermore, ccRCC tissue had an obvious richness in the Vα-Jα and Vβ-Dβ-Jβ clonotypes compared to peritumour tissue (Fig. 3G, I). The clonotypes of Vβ-Jβ were nearly identical in the two groups (Fig. 3H). Complete information on the TRA/B V-J pair is available in Additional file 4.

### Expanded TCR clonotypes in ccRCC tissue

**Fig. 4:**
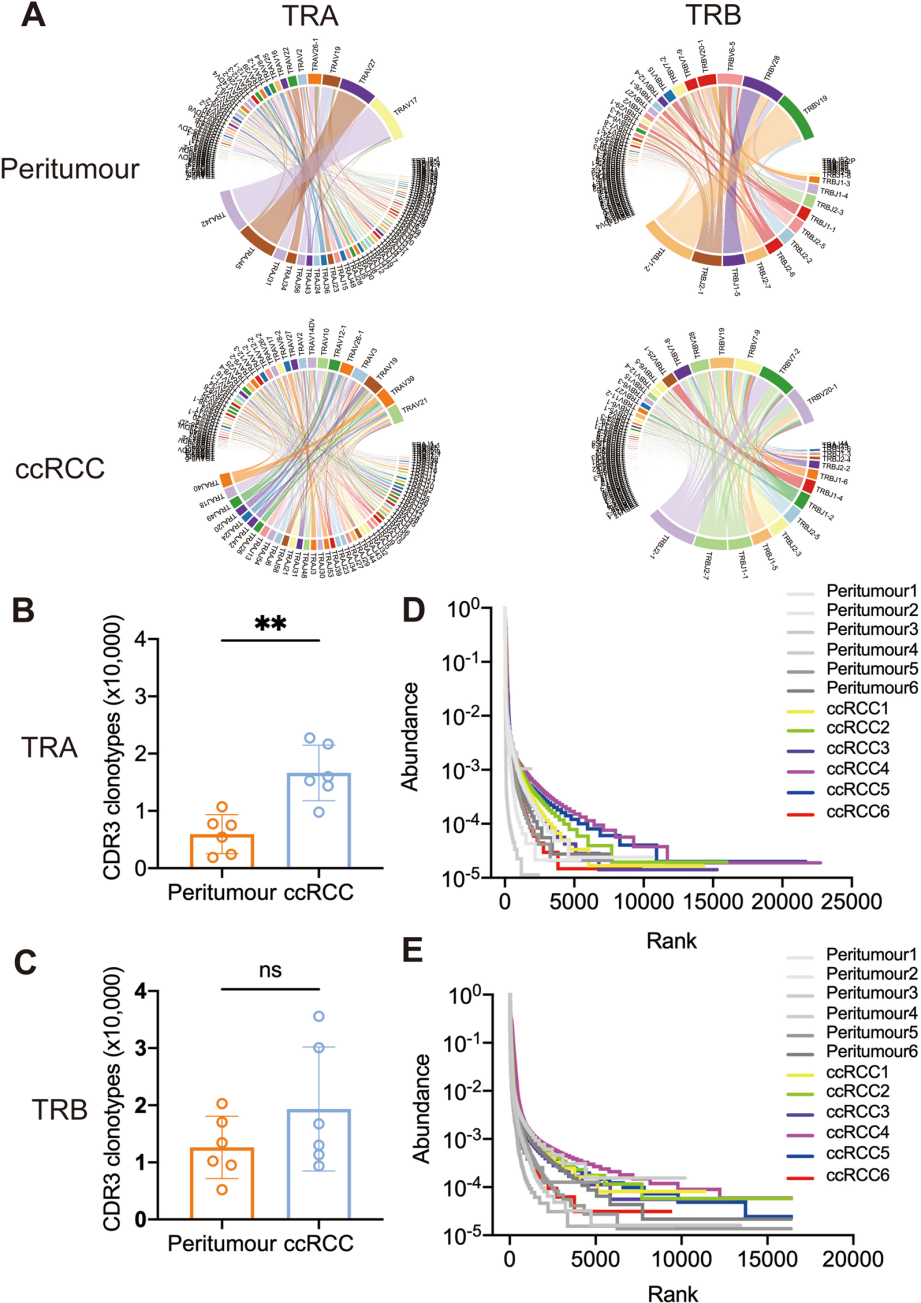
CDR3 aa diversity increases in ccRCC rather than in peritumour tissue. **A** Circular plots representing the V-CDR3-J combination in peritumour and ccRCC tissue. **B**, **C** Comparison of CDR3 aa clonotypes for TRA and TRB in the ccRCC and peritumour groups. **D**, **E** Rank-abundance curves of TRA and TRB indicate upregulated CDR3 clonotypes in ccRCC compared with peritumour tissue. Data are mean ± SEM. Student’s t-test is used to compare CDR3aa clonotypes difference for TRA and TRB in ccRCC and peritumour groups. *P* value > 0.05; *P value < 0.05; **P value < 0.01; ***P value < 0.001; ns, not significant

**Fig. 5:**
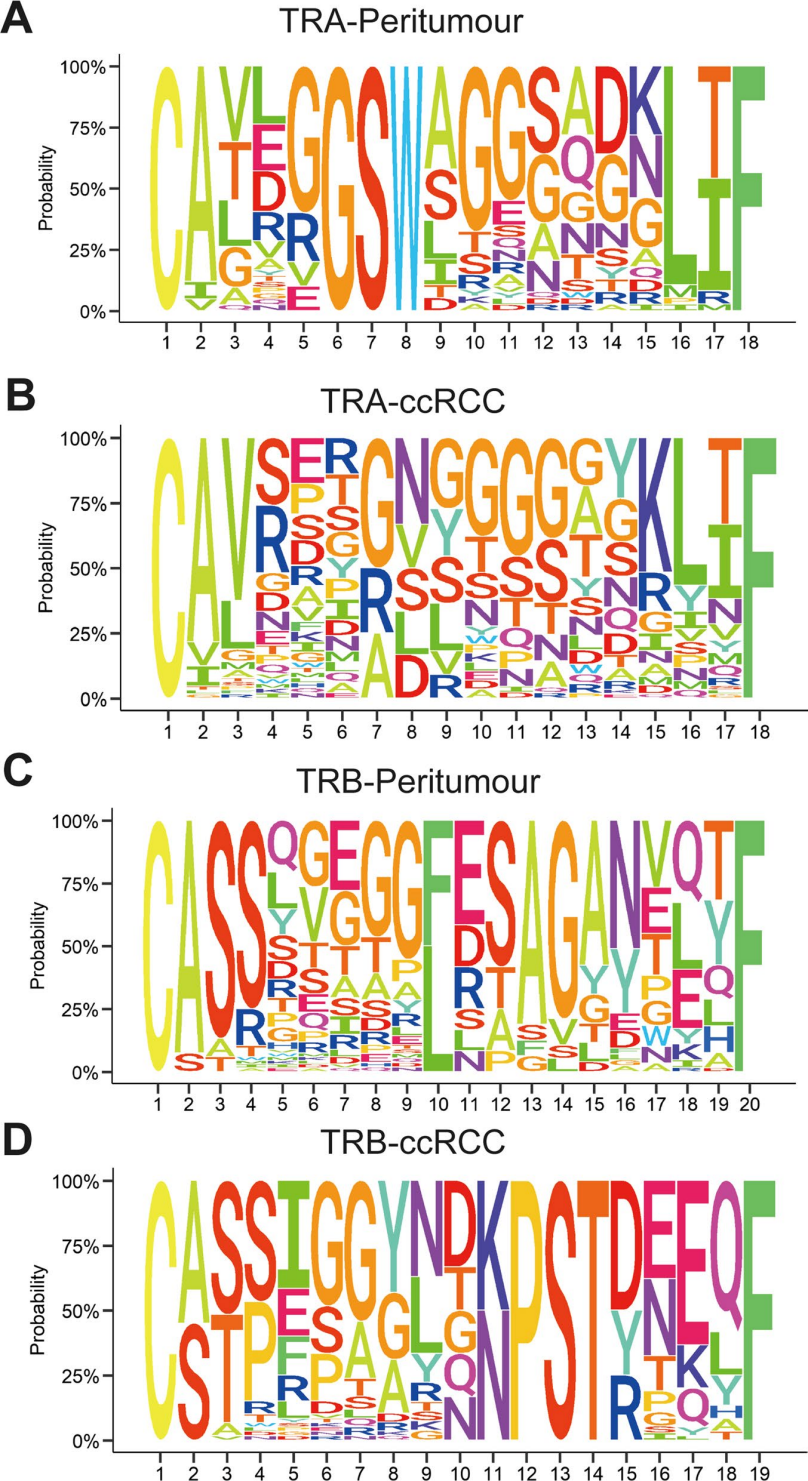
CDR3 aa motifs of TRA and TRB in ccRCC and peritumour tissues

Given CDR3 amino acid (aa) clonotypes in determining TCR diversity, we detected the CDR3 aa clones in each sample. Of note, a richer CDR3 aa of the alpha chain was observed (16,639 *vs.* 5941, *P* = 0.002) in ccRCC than in peritumour tissue, and no difference was observed in the CDR3 aa of the beta chain (19,344 *vs.* 12,623, *P* = 0.205). Supporting information is listed in Additional file 3 and Fig. 4C, D. In addition, compared to adjacent tissue, ccRCC led to diversified clonotypes of Vα-CDR3-Jα and Vβ-CDR3-Jβ combinations (Fig. 4A, B); Rank-abundance analysis also revealed an expanded TCR richness and evenness in ccRCC tissue (Fig. 4E, F). The Gini coefficient, Simpson index and Shannon diversity showed no difference in CDR3 aa diversity between ccRCC and adjacent tissues (Additional file 8: Fig. S3). Then, we determined the preferential pairings of CDR3 aa motifs. A total of 458 CDR3 aa consensus motifs (frequency > 1.00% CDR3 motifs) were identified for specific ccRCC-TCRα (126 CDR3 aa), peritumour-TCRα (98 CDR3 aa), ccRCC-TCRβ (110 CDR3 aa) and peritumour-TCRβ (124 CDR3 aa). The sequence logo of CDR3 aa in ccRCC has more varieties than it does in the peritumour group (Fig. 5A–D). Additionally, we used VDJtools to access existing information on TCR antigen in the ccRCC group. The potential antigens (> 0.01%) recognized by infiltrating TCRs in ccRCC are listed in Table 3 and Additional file 5. These antigens include the MHC class, antigen species, antigen gene, and antigen epitope in ccRCC-TCRα/β cells. Among all antigens, NLVPMVATV, KLGGALQAK, and RLRAEAQVK were the top 3 most frequent epitopes. We also listed the potential antigens (> 0.01%) recognized by infiltrating TCRs in peritumour group (Additional file 9: Table S1). Of all potential antigens between peritumour and ccRCC tissue, MHI class, CMV, SARS-Cov2, HCV, and HIV-1 was the shared antigen.

**Table 3:** Analysis of antigen in ccRCC

Antigen name	TRA		TRB	
	Antigen value	Frequency (%)	Antigen value	Frequency (%)
MHC. class	MHCI	7.74	MHCI	2.83
Antigen. species	CMV	6.01	CMV	1.93
	EBV	2.65	EBV	0.49
	InfluenzaA	0.62	SARS-CoV-2	0.33
	HomoSapiens	0.53	InfluenzaA	0.29
	HCV	0.49	HomoSapiens	0.24
	SARS-CoV-2	0.43	HIV-1	0.09
	HIV-1	0.43	HIV	0.09
	YFV	0.18		
Antigen. gene	IE1	4.24	IE1	1.85
	pp65	1.81	pp65	0.36
	EBNA4	1.64	BZLF1	0.32
	EBNA3A	1.36	ORF14	0.32
	M	0.55	M	0.29
	BZLF1	0.46	EBNA4	0.20
	Spike	0.43	SF3B1	0.18
	Gag	0.43	Nef	0.09
	BST2	0.42	Gag	0.09
	NSP3	0.36	MLANA	0.06
	NS3	0.25	LMP2A	0.03
	CORE	0.23		
	NS4B	0.18		
	PMEL	0.11		
Antigen. epitope	KLGGALQAK	4.24	KLGGALQAK	1.85
	NLVPMVATV	1.81	NLVPMVATV	0.35
	AVFDRKSDAK	1.63	LLLEWLAMA	0.32
	RLRAEAQVK	1.35	GILGFVFTL	0.29
	GILGFVFTL	0.55	GLCTLVAML	0.26
	RAKFKQLL	0.46	RLPGVLPRA	0.18
	SLFNTVATLY	0.42	AVFDRKSDAK	0.17
	LLLGIGILV	0.42	KAFSPEVIPMF	0.09
	IVTDFSVIK	0.40	HTQGYFPD	0.09
	YLQPRTFLL	0.38	ELAGIGILTV	0.06
	TTDPSFLGRY	0.36	RAKFKQLL	0.06
	ATDALMTGF	0.25	IVTDFSVIK	0.04
	GPRLGVRAT	0.23	FLYALALLL	0.03
	LLWNGPMAV	0.18		
	KTWGQYWQV	0.11		

*ccRCC* clear cell renal cell cancer

## Discussion

The infiltrated amount, phenotype, and location of T cells determine the adaptive cellular immune reaction area [18, 19]. However, the contribution of T cells to ccRCC tumour cell elimination remains unclear [20]. In our study, higher CD3^+^, CD4^+^, and CD8^+^ T-cell infiltrations were found in peritumour tissue, which indicated that peritumours are the specific locations of adaptive cellular immune reactions. Similar results have been reported in oral squamous cell carcinoma [21–23]. The mechanism by which tumour tissue resists T-cell infiltration could be associated with immunosuppressive factors in the TME. In the TME, tumour-associated macrophages, neutrophils and myeloid-derived suppressor cells (MDSCs) and cytokines such as IL-6, IL-10, and TGF-β can participate in the formation of an immunosuppressive microenvironment by altering and modifying systems with high plasticity components, leading to immune escape of tumour cells and promoting tumour progression [24]. For instance, MDSCs migrate to inflammatory and hypoxic tumour tissues and produce large amounts of immunosuppressive cytokines. MDSCs could also increase the expression of PDL1 and CTLA4 receptors and inhibit T-cell cytotoxicity. Overall, the dynamic and complex TME remains a barrier to the T-cell response and the therapeutic success of immunotherapeutic approaches. However, modulation of the TME may lead to an effective inflammatory response. For instance, Choi et al*.* [25] used IL-12 to intervene in the TME, differentiated a large number of MDSCs into antigen-presenting cells (such as Dendric cells) and restored the immune function of T cells and macrophages, which provides a new strategy for ccRCC treatment.

To further investigate the T-cell difference between ccRCC and peritumour tissue, we compared the preference biases of the TCR immune repertoire in ccRCC and peritumour tissue. The Vβ and Jβ genes with differential usage in our study were also inconsistent with this study, except for the TRAV21 gene, which was higher in tumour tissues than in peritumour tissues in both studies [26–28]. Interestingly, the TCR clonotypes have been observed to be rich in ccRCC tissue, although the count of T-cell infiltration presented the opposite result. This specific CDR3 aa is necessary to select specific TCRs but is insufficient to define specific antibody-binding properties unless combined with appropriate VL and VH germline genes. In our study, every ccRCC sample had over 20,000 V-CDR3-J sequences, and only a dozen sequences were highly frequent. When we matched these TCRs and their recognizable antigens by VDJtools package, over 80% of TCRs did not match to antigens in its databases. Among all matched antigens, the MHC I molecule is the potential recognition by TCRs in ccRCC and peritumour tissue, though the correlation of CD8^+^ T cells between adjacent and ccRCC tissue has not been found. That implied that MHC class I expression by target cells is essential for T cell recognition and killing, both in peritumour or ccRCC tissue, and that effect is not associated with T cell location. Therefore, the Table 2 result does not conflict with the Table 3 results. Besides, some known epitopes also are marked as a high frequency that can be recognized by peritumour and ccRCC TCRs, suggesting infiltrating TCR can also target the virus that is not relevant to ccRCC. Of course, these matched antigens may be a potential target for engineering T-cell therapy against cancer proliferation, invasion, and metastasis. However, whether the high-frequency TCRs based on our data have a targeting function still needs further validation in ccRCC patients through single-cell sequencing and clinical verification.

Collectively, this study highlights an important finding that ccRCC is characterized by higher TCR diversity in ccRCC, and different from other studies, we also predicted several antigens in ccRCC tissue with higher frequencies recognized by infiltrating TCR, which may provide a better understanding of the TME of ccRCC. Moreover, the high frequencies of ccRCC infiltrating TCR in our study also give guidance to researchers and clinicians to conduct novel immunotherapy for ccRCC patients, particularly individual TCR-T therapy.

Indeed, this study also has some limitations, a V and J and the constant region primer mixture were used when preparing samples by multiplex PCR, and it causes preferential amplification of high abundance gene products occurs during PCR, resulting in inaccurately reported cloning frequencies. Besides, IR-seq cannot identify the exact TCR α/β pairs that participate in killing cancer cells (only draw inferences based on clone frequency changes). However, IR-seq technology has matured, it has the ability to capture maximum clones and provide an architecture of the TCR repertoire information of ccRCC patients [29].

## Conclusions

The current study collectively demonstrates diminished T-cell infiltration and increased CDR3 aa diversity in ccRCC, which may be associated with novel immunotherapeutic targets for ccRCC patients.

## Supplementary Information

**Additional file 1.** Comparison of TRAV/J usage.

**Additional file 2.** Comparison of TRBV/J usage.

**Additional file 3.** Comparison of TRA/B CDR3 AA usage.

**Additional file 4.** Comparison of TRA/ BV-J combination usage.

**Additional file 5.** Specific antigen of ccRCC.

**Additional file 6: Figure S1.** Representative images of CD3, CD4, and CD8 immunohistochemistry results in normal renal cortex. Scale bar, 100 μm.

**Additional file 7: Figure S2.** Representative images of haematoxylin and eosin staining in peritumour (Left) and ccRCC (Right) tissue. Scale bar, 100 μm.

**Additional file 8: Figure S3.** Comparison of the peritumor and ccRCC tissue TCR repertoire diversity by Gini coefficient (A, B), Shannon diversity entropy (C, D), and Simpson index (E, F). Data are mean ± SEM. Student’s t-test is used to calculate the Gini coefficient, Shannon diversity entropy, and Simpson index difference in the ccRCC and peritumour groups. P value >0.05; *P value <0.05; **P value <0.01; ***P value <0.001; ns, not significant.

**Additional file 9: Table S1.** Analysis of antigen in Peritumour tissue.

## Data Availability

The IR-seq sequenced raw data have been deposited in the NGDC GSA database (https://bigd.big.ac.cn/gsa, accession code: HRA002339).

## Abbreviations

*ccRCC*: Clear cell renal cell cancer
*TCR*: T-cell receptor
*IHC*: Immunohistochemistry
*HE*: Haematoxylin and eosin
*IR-Seq*: Immune repertoire sequencing
*CDR3*: Complementarity-determining region 3
*AA*: Amino acid
*TIL*: Tumour-infiltrating lymphocyte
*TME*: Tumour microenvironment
*IMGT*: International Immunogenetics Information System
*MDSCs*: Myeloid-derived suppressor cells
