# Supplementary material for: The intrarenal landscape of T cell receptor repertoire in clear cell renal cell cancer
Source: J Transl Med. 2022 Dec 3;20:558. doi: 10.1186/s12967-022-03771-3 (PMC9719196; doi:10.1186/s12967-022-03771-3)
Supplement: Supplementary file 1 — Additional file 1. Comparison of TRAV/J usage. [file 12967_2022_3771_MOESM1_ESM.docx]

| Additional file 1. Comparison of TRAV/J usage | | | | | | | | | | | | |
| --- | --- | --- | --- | --- | --- | --- | --- | --- | --- | --- | --- | --- |
|  | Peritumour1 | Peritumour2 | Peritumour3 | Peritumour4 | Peritumour5 | Peritumour6 | ccRCC1 | ccRCC2 | ccRCC3 | ccRCC4 | ccRCC5 | ccRCC6 |
| TRAJ34 | 38.93 | 35.35 | 30.34 | 18.22 | 295480.48 | 594.05 | 47808.25 | 31275.96 | 53.21 | 12347.15 | 22334.54 | 10383.84 |
| TRAJ44 | 14.03 | 17955.98 | 11.64 | 6.00 | 16.59 | 27.65 | 105769.63 | 23096.02 | 28.39 | 36686.48 | 1215.57 | 15.32 |
| TRAJ54 | 14.74 | 7037.99 | 10.33 | 6.89 | 18.74 | 48.93 | 22630.87 | 29405.16 | 39.69 | 4202.65 | 164181.24 | 16.98 |
| TRAJ3 | 15.17 | 19740.93 | 10.85 | 8.33 | 22502.58 | 44910.92 | 13674.22 | 3591.45 | 6915.25 | 4772.69 | 10288.27 | 127104.33 |
| TRAJ4 | 12.02 | 24545.05 | 8.89 | 4.89 | 21591.64 | 45923.84 | 18.23 | 2038.53 | 45511.23 | 19349.15 | 7909.55 | 15870.53 |
| TRAJ22 | 7.30 | 4689.93 | 4.71 | 4.78 | 12.73 | 72478.92 | 22023.64 | 7138.46 | 12.29 | 8709.56 | 205.94 | 24117.81 |
| TRAJ5 | 54980.72 | 43405.36 | 10.72 | 6.67 | 13.87 | 29.12 | 10841.78 | 6062.58 | 65.25 | 17389.55 | 17407.48 | 47393.22 |
| TRAJ38 | 3.58 | 19688.81 | 1.70 | 1.00 | 2.86 | 5.63 | 234.87 | 8240.63 | 4.55 | 2604.66 | 3350.33 | 2.48 |
| TRAJ8 | 14.89 | 80621.02 | 6.54 | 3.89 | 10.73 | 14101.09 | 12.70 | 5001.31 | 17.08 | 10689.24 | 25782.18 | 17101.89 |
| TRAJ53 | 8.73 | 8027.51 | 8.76 | 6.67 | 12.73 | 21.53 | 15324.09 | 20155.83 | 556.76 | 32478.85 | 8951.91 | 74666.95 |
| TRAJ30 | 17.75 | 20562.79 | 15.56 | 7.89 | 21.74 | 121657.07 | 15735.88 | 23698.11 | 20224.62 | 38543.76 | 27841.20 | 28517.37 |
| TRAJ9 | 12.45 | 13722.07 | 6.67 | 6.22 | 35447.10 | 18.11 | 54202.71 | 4390.65 | 8922.35 | 8909.54 | 5649.55 | 7763.35 |
| TRAJ41 | 1.86 | 2.72 | 1.05 | 1.00 | 2.15 | 4.65 | 1693.76 | 4519.84 | 6.51 | 22182.91 | 15.42 | 2.35 |
| TRAJ17 | 11.02 | 8457.10 | 8.11 | 4.67 | 48699.55 | 38427.76 | 9.86 | 18.60 | 10943.59 | 8103.75 | 21007.09 | 12.97 |
| TRAJ56 | 231154.82 | 60.12 | 20.66 | 15.67 | 70.51 | 10309.74 | 9154.37 | 51341.43 | 2081.57 | 74.10 | 7556.27 | 53.00 |
| TRAJ16 | 3.72 | 1808.36 | 1.57 | 1.44 | 4.15 | 24754.10 | 5796.56 | 1493.57 | 9626.08 | 4435.38 | 8727.98 | 3.59 |
| TRAJ20 | 19.47 | 13437.19 | 15.56 | 9.22 | 35694.82 | 9756.31 | 10037.78 | 35613.61 | 109710.12 | 37585.23 | 15024.81 | 25.12 |
| TRAJ12 | 14568.08 | 17549.81 | 9.55 | 6.22 | 47393.28 | 9466.13 | 12988.26 | 18.60 | 676.57 | 44819.81 | 10302.32 | 12.28 |
| TRAJ7 | 0.00 | 0.00 | 0.13 | 0.11 | 0.29 | 0.73 | 0.14 | 0.30 | 0.49 | 1923.23 | 464.99 | 0.14 |
| TRAJ32 | 5.87 | 10.88 | 5.49 | 2.78 | 7.72 | 25448.95 | 34385.24 | 17083.94 | 2413.46 | 35755.88 | 19744.57 | 10.21 |
| TRAJ35 | 5.15 | 6.50 | 3.53 | 1.56 | 4.86 | 9.05 | 28893.10 | 7.53 | 9.95 | 18146.58 | 12745.63 | 4.83 |
| TRAJ21 | 22.33 | 71086.03 | 14.91 | 9.33 | 22.03 | 35.72 | 30.12 | 6331.73 | 39631.60 | 18158.96 | 4748.88 | 101464.88 |
| TRAJ10 | 5.73 | 9.52 | 5.23 | 2.67 | 30715.05 | 12.23 | 7.43 | 12812.73 | 20370.23 | 9059.71 | 5370.63 | 1478.44 |
| TRAJ11 | 5.30 | 14138.21 | 3.27 | 1.44 | 5.15 | 9.79 | 6.62 | 4225.14 | 24169.77 | 2795.43 | 11988.87 | 5.11 |
| TRAJ18 | 16.46 | 21.75 | 18.31 | 10.67 | 20.74 | 38.66 | 30.93 | 177.76 | 117710.88 | 4934.33 | 10439.55 | 180229.38 |
| TRAJ43 | 742.17 | 38846.09 | 20.53 | 12.33 | 137996.74 | 50.16 | 30273.40 | 35370.44 | 4648.87 | 17473.31 | 17695.65 | 29.12 |
| TRAJ47 | 19.75 | 52832.64 | 9.02 | 5.89 | 30946.89 | 20.31 | 10033.87 | 12712.33 | 7415.61 | 2308.39 | 21.42 | 15.87 |
| TRAJ36 | 98835.12 | 64845.89 | 17.65 | 8.22 | 30.18 | 59.70 | 30587.54 | 2482.64 | 42.52 | 36699.46 | 2198.83 | 7251.12 |
| TRAJ37 | 13.60 | 43487.68 | 6.28 | 5.67 | 9901.76 | 25501.06 | 2785.57 | 2839.94 | 35536.05 | 6020.69 | 9857.21 | 12.28 |
| TRAJ33 | 7.30 | 18496.58 | 4.45 | 3.89 | 10.15 | 15068.25 | 7282.73 | 53489.36 | 17.69 | 18114.89 | 7770.26 | 10.63 |
| TRAJ52 | 229.31 | 20030.64 | 5.10 | 2.89 | 12334.36 | 1343.46 | 1446.47 | 2644.16 | 20263.20 | 17987.96 | 13388.28 | 5.52 |
| TRAJ49 | 27.63 | 35870.27 | 19.35 | 15.78 | 41935.09 | 54.56 | 18199.21 | 85217.71 | 102057.10 | 32206.88 | 37894.07 | 29.40 |
| TRAJ24 | 40070.53 | 39.73 | 19.87 | 15.56 | 130042.86 | 63.86 | 102893.57 | 5605.32 | 42059.08 | 20284.28 | 18638.98 | 41.96 |
| TRAJ42 | 89.75 | 15322.59 | 106.04 | 999604.89 | 120.71 | 171.02 | 10486.45 | 158223.45 | 777.82 | 22415.04 | 16400.24 | 142.86 |
| TRAJ6 | 14.89 | 18.13 | 9.28 | 7.00 | 16.31 | 75963.70 | 32300.08 | 34725.53 | 7722.07 | 9799.54 | 82397.12 | 27505.75 |
| TRAJ58 | 14.31 | 19.49 | 9.02 | 7.67 | 17.31 | 26.67 | 89448.27 | 58893.44 | 30.11 | 36571.62 | 224.95 | 21.26 |
| TRAJ57 | 16595.64 | 5.74 | 2.62 | 2.44 | 6.44 | 8949.40 | 7273.28 | 18585.19 | 8.85 | 11280.57 | 14614.31 | 6.63 |
| TRAJ23 | 129427.58 | 21608.50 | 20.79 | 10.11 | 35.18 | 30168.32 | 43.89 | 13444.65 | 24779.98 | 34198.18 | 69360.58 | 31.33 |
| TRAJ48 | 25.05 | 18458.07 | 17.13 | 12.11 | 36.76 | 189935.07 | 18085.62 | 8487.93 | 4545.90 | 32940.53 | 26527.29 | 87286.74 |
| TRAJ15 | 73505.73 | 9262.19 | 15.30 | 9.11 | 73109.68 | 39.39 | 6477.79 | 12865.88 | 22910.50 | 6202.40 | 15288.32 | 26965.92 |
| TRAJ27 | 6.15 | 65.10 | 323.36 | 4.33 | 10.73 | 26093.89 | 14894.88 | 17382.03 | 35795.08 | 22989.15 | 26269.44 | 13.11 |
| TRAJ31 | 288855.89 | 67316.60 | 35.17 | 21.44 | 76.23 | 126.49 | 1165.69 | 70268.27 | 5101.44 | 62275.31 | 26700.33 | 9379.40 |
| TRAJ29 | 9.88 | 12918.49 | 8.63 | 6.44 | 12.59 | 54285.79 | 9948.78 | 13071.11 | 4975.85 | 58325.46 | 24989.61 | 13142.37 |
| TRAJ50 | 1.43 | 3.32 | 2.22 | 1.00 | 2.57 | 54151.47 | 587.50 | 3033.21 | 247.85 | 1210.57 | 2287.92 | 2.76 |
| TRAJ28 | 14.89 | 63503.97 | 5456.41 | 5.78 | 14.45 | 57517.34 | 33479.54 | 9593.79 | 2486.21 | 4462.84 | 16678.82 | 17.12 |
| TRAJ26 | 18.89 | 35445.67 | 12.03 | 8.22 | 19.59 | 4519.72 | 120044.18 | 24492.14 | 21616.59 | 6630.73 | 16011.32 | 21.53 |
| TRAJ39 | 13.17 | 23440.88 | 11.77 | 7.67 | 25383.57 | 20.80 | 17997.83 | 5515.41 | 42637.22 | 62141.14 | 3569.46 | 16.29 |
| TRAJ45 | 50456.39 | 64860.24 | 993600.19 | 48.44 | 121.57 | 12084.55 | 12433.04 | 19413.77 | 6685.22 | 14499.64 | 18818.54 | 100.62 |
| TRAJ40 | 31.63 | 33.23 | 22.75 | 16.00 | 31.75 | 13942.30 | 2619.05 | 12547.86 | 183315.37 | 12078.66 | 102577.21 | 85339.27 |
| TRAJ13 | 13.17 | 6647.37 | 10.98 | 8.89 | 14.45 | 11752.05 | 7900.76 | 21354.99 | 4622.33 | 46224.17 | 6565.13 | 106354.73 |
