# Supplementary material for: The intrarenal landscape of T cell receptor repertoire in clear cell renal cell cancer
Source: J Transl Med. 2022 Dec 3;20:558. doi: 10.1186/s12967-022-03771-3 (PMC9719196; doi:10.1186/s12967-022-03771-3)
Supplement: Supplementary file 2 — Additional file 2. Comparison of TRBV/J usage. [file 12967_2022_3771_MOESM2_ESM.docx]

| Additional file 2. Comparison of TRBV/J usage | | | | | | | | | | | | |
| --- | --- | --- | --- | --- | --- | --- | --- | --- | --- | --- | --- | --- |
|  | Peritumour1 | Peritumour2 | Peritumour3 | Peritumour4 | Peritumour5 | Peritumour6 | ccRCC1 | ccRCC2 | ccRCC3 | ccRCC4 | ccRCC5 | ccRCC6 |
| TRBV29-1 | 11904.80 | 4706.48 | 32337.57 | 6.45 | 13133.89 | 28566.77 | 2236.65 | 6243.42 | 14049.43 | 6825.75 | 2009.14 | 993.66 |
| TRBV29/OR9-2 | 3.14 | 32.72 | 6.92 | 0.00 | 20.21 | 62.14 | 9.46 | 19.43 | 61.41 | 29.02 | 16.29 | 9.11 |
| TRBV3-2 | 2.67 | 978.99 | 0.77 | 1.18 | 5.36 | 6871.16 | 585.18 | 529.70 | 455.75 | 575.27 | 172.02 | 652.22 |
| TRBV6-1 | 714.99 | 6134.39 | 1714.34 | 38.44 | 123735.62 | 1033.88 | 30721.16 | 12520.74 | 3617.87 | 10437.32 | 5911.72 | 359.07 |
| TRBV24-1 | 13.18 | 1223.74 | 12237.80 | 3.29 | 4883.65 | 531.92 | 2181.92 | 626.42 | 424.36 | 2603.09 | 518.73 | 3063.62 |
| TRBV13 | 18.51 | 10.47 | 1.08 | 0.13 | 5.50 | 322.17 | 27.03 | 10.85 | 75.27 | 79.73 | 8.25 | 7.59 |
| TRBV11-3 | 67.14 | 506.51 | 17.37 | 5.27 | 327.94 | 612.77 | 1587.95 | 1139.85 | 419.28 | 1165.67 | 460.16 | 65.01 |
| TRBV7-8 | 53461.96 | 137076.88 | 16199.14 | 1728.34 | 11734.67 | 91701.68 | 106869.29 | 191355.84 | 124356.83 | 133166.12 | 156927.10 | 25405.73 |
| TRBV24/OR9-2 | 13.80 | 1215.88 | 12179.40 | 4.74 | 4923.39 | 520.86 | 2147.45 | 645.86 | 436.36 | 2606.37 | 504.30 | 3038.41 |
| TRBV26 | 7.53 | 2.62 | 3.53 | 0.00 | 1.79 | 10.46 | 2.70 | 0.45 | 0.46 | 7.06 | 5.98 | 0.00 |
| TRBV6-9 | 19.76 | 43.19 | 178.89 | 0.00 | 64.08 | 17.30 | 14.87 | 23.05 | 54.95 | 104.46 | 11.34 | 47.39 |
| TRBV20-1 | 34.20 | 226.42 | 5.23 | 2.24 | 32.73 | 23905.36 | 72.30 | 125.65 | 93.28 | 5048.73 | 1932.42 | 21.57 |
| TRBV19 | 722.05 | 135371.50 | 317.36 | 937310.28 | 71760.24 | 54957.58 | 24101.76 | 83953.99 | 80520.35 | 104577.09 | 38608.93 | 286353.96 |
| TRBV6-3 | 571.62 | 2070.54 | 25271.06 | 20.27 | 1475.94 | 18794.67 | 1737.96 | 6670.07 | 10705.85 | 2509.98 | 4591.68 | 34593.86 |
| TRBV11-2 | 96.00 | 158.37 | 2.00 | 1.45 | 37.68 | 764.40 | 348.00 | 401.80 | 154.23 | 816.73 | 293.30 | 8.81 |
| TRBV16 | 0.00 | 0.00 | 0.00 | 0.00 | 0.00 | 0.00 | 0.00 | 23.05 | 164.85 | 0.25 | 1.24 | 0.00 |
| TRBV6-5 | 20843.44 | 50130.03 | 262058.53 | 75.57 | 10012.74 | 22004.31 | 21140.06 | 18784.95 | 23944.43 | 37496.85 | 15219.45 | 5121.12 |
| TRBV7-3 | 79.69 | 1179.24 | 44810.65 | 10.93 | 402.19 | 782.70 | 912.90 | 323.61 | 387.88 | 1250.20 | 616.71 | 80.20 |
| TRBV20/OR9-2 | 106266.35 | 138087.28 | 173.82 | 165.62 | 254664.51 | 25642.30 | 252506.26 | 87505.97 | 307014.70 | 114273.63 | 186157.55 | 179759.99 |
| TRBV4-2 | 2338.23 | 1459.32 | 2.31 | 136.13 | 81.81 | 45.65 | 991.29 | 5800.95 | 1607.84 | 1736.40 | 1464.01 | 73.82 |
| TRBV27 | 111.69 | 51362.93 | 41.34 | 36.99 | 38171.45 | 100589.72 | 13104.33 | 12725.93 | 67830.79 | 19407.21 | 15062.91 | 956.91 |
| TRBV30 | 4.39 | 569.33 | 3488.15 | 1.58 | 3704.16 | 1434.48 | 286.51 | 111.18 | 62.80 | 215.98 | 299.48 | 8.51 |
| TRBV4-3 | 727.70 | 793.14 | 7.07 | 13918.45 | 4233.82 | 795.37 | 514.90 | 465.52 | 25035.57 | 366.61 | 2669.16 | 310.77 |
| TRBV12-2 | 0.00 | 0.00 | 0.00 | 0.00 | 0.14 | 0.20 | 0.00 | 0.00 | 0.00 | 0.00 | 0.00 | 0.00 |
| TRBV5-1 | 7.53 | 36485.69 | 4.61 | 8.56 | 9.35 | 43509.48 | 11444.75 | 19151.95 | 19880.04 | 12772.71 | 9130.77 | 5989.02 |
| TRBV6-8 | 21667.14 | 48969.12 | 176399.94 | 51.21 | 7154.90 | 22455.39 | 22369.20 | 16570.33 | 23147.44 | 38000.21 | 13424.82 | 1556.87 |
| TRBV5-5 | 181.96 | 321.97 | 1.23 | 1.84 | 291.78 | 39.82 | 1048.05 | 1070.70 | 114.52 | 450.63 | 671.57 | 28.25 |
| TRBV15 | 107440.64 | 646.55 | 22.59 | 18.43 | 79742.90 | 18403.93 | 2423.15 | 898.96 | 3197.67 | 2157.51 | 38123.20 | 43567.52 |
| TRBV7-4 | 18.51 | 91.62 | 52.87 | 0.39 | 281.47 | 1635.19 | 192.58 | 210.61 | 66.03 | 306.30 | 265.66 | 31.90 |
| TRBV28 | 333209.15 | 23494.44 | 344202.22 | 182.08 | 136936.34 | 223445.39 | 30868.47 | 7428.92 | 8392.44 | 135781.32 | 148141.40 | 37290.82 |
| TRBV14 | 0.00 | 5.24 | 112.80 | 0.00 | 6.74 | 13.27 | 0.68 | 0.90 | 87.73 | 0.00 | 0.41 | 12.76 |
| TRBV7-9 | 2381.53 | 107267.43 | 2030.92 | 148.90 | 126149.46 | 114728.21 | 267603.99 | 135483.68 | 84417.12 | 133977.55 | 101828.17 | 33801.60 |
| TRBV5-7 | 0.16 | 2.62 | 0.00 | 0.00 | 25.71 | 1.61 | 1.35 | 9.49 | 0.92 | 3.78 | 30.11 | 0.00 |
| TRBV12-5 | 30.12 | 2630.71 | 20.29 | 4.87 | 17807.99 | 49.07 | 82.44 | 5700.16 | 869.95 | 61.56 | 620.42 | 45.57 |
| TRBV5-3 | 0.31 | 1.31 | 0.00 | 0.00 | 0.28 | 0.80 | 0.68 | 2.71 | 0.92 | 0.50 | 194.91 | 0.00 |
| TRBV7-6 | 2.82 | 3648.96 | 2.00 | 0.26 | 18.29 | 3528.79 | 2217.05 | 402.25 | 29.55 | 1344.56 | 2853.97 | 9.11 |
| TRBV6-2 | 564.24 | 1977.61 | 24516.94 | 22.64 | 1423.97 | 18650.48 | 1739.99 | 6703.52 | 9171.89 | 2446.91 | 4348.09 | 34589.91 |
| TRBV6-7 | 0.16 | 1.31 | 11.83 | 0.00 | 0.41 | 0.00 | 1.35 | 0.45 | 0.92 | 23.21 | 0.62 | 0.30 |
| TRBV5-8 | 529.42 | 218.57 | 0.61 | 0.92 | 8.94 | 28.76 | 164.20 | 1378.04 | 115.90 | 428.17 | 948.99 | 14.28 |
| TRBV10-2 | 0.31 | 388.72 | 0.00 | 0.00 | 0.28 | 0.20 | 168.26 | 230.95 | 0.92 | 122.37 | 0.62 | 0.00 |
| TRBV4-1 | 2712.51 | 1985.46 | 13.52 | 45716.27 | 97.35 | 4080.22 | 1404.16 | 1413.29 | 1336.33 | 1105.62 | 507.80 | 812.31 |
| TRBV7-1 | 0.00 | 0.00 | 0.00 | 0.00 | 0.00 | 0.20 | 0.68 | 0.45 | 0.00 | 0.50 | 0.00 | 0.00 |
| TRBV10-3 | 7.22 | 586.35 | 6.76 | 2.50 | 9542.35 | 1601.60 | 355.43 | 151.41 | 253.97 | 471.06 | 118.39 | 18.83 |
| TRBV7-7 | 0.00 | 95.54 | 0.00 | 0.00 | 0.14 | 4.02 | 6.76 | 46.10 | 0.46 | 40.37 | 9.28 | 0.30 |
| TRBV10-1 | 0.31 | 624.30 | 0.00 | 0.13 | 0.28 | 0.40 | 94.60 | 5.42 | 29.09 | 65.60 | 237.19 | 0.91 |
| TRBV2 | 103900.51 | 11970.37 | 23.21 | 23.43 | 118.11 | 15556.68 | 8055.32 | 8541.20 | 2886.45 | 9789.89 | 4689.24 | 1983.38 |
| TRBV6-6 | 487.54 | 2972.31 | 6307.47 | 6.32 | 484.28 | 883.05 | 1091.97 | 1217.59 | 3147.80 | 2562.97 | 1259.61 | 800.46 |
| TRBV12-3 | 186.51 | 306.26 | 15618.52 | 10.40 | 21930.98 | 304.27 | 1020.34 | 338.52 | 139.91 | 3631.25 | 2003.78 | 11703.43 |
| TRBV5-4 | 166.59 | 116.48 | 0.00 | 0.13 | 1.24 | 2.21 | 5.41 | 524.73 | 65.11 | 113.04 | 48.47 | 0.61 |
| TRBV9 | 525.97 | 223.81 | 1.84 | 0.79 | 179.58 | 215.79 | 84.47 | 87.23 | 40.63 | 79.48 | 105.81 | 5.47 |
| TRBV7-2 | 101537.65 | 201234.73 | 3309.42 | 230.66 | 12518.30 | 139082.03 | 176290.57 | 276537.82 | 121816.24 | 178629.16 | 216605.13 | 22786.84 |
| TRBV18 | 0.00 | 17.01 | 0.00 | 0.00 | 0.28 | 48.06 | 10.14 | 14.01 | 10.16 | 30.02 | 31.97 | 0.61 |
| TRBV23-1 | 0.00 | 0.00 | 0.00 | 0.00 | 0.00 | 0.00 | 0.00 | 0.00 | 0.00 | 0.00 | 0.21 | 0.00 |
| TRBV6-4 | 92519.48 | 903.08 | 27.97 | 14.35 | 780.18 | 194.87 | 880.47 | 479.53 | 301.99 | 7630.37 | 3202.96 | 155.54 |
| TRBV5-6 | 0.63 | 342.91 | 0.77 | 0.39 | 1569.99 | 29.36 | 4.73 | 351.18 | 6.93 | 74.94 | 1472.67 | 0.91 |
| TRBV11-1 | 0.47 | 24.87 | 0.00 | 1.32 | 74.39 | 17.09 | 66.22 | 34.35 | 27.71 | 37.59 | 33.00 | 6.08 |
| TRBV3-1 | 0.00 | 0.00 | 0.00 | 0.00 | 0.00 | 0.20 | 0.00 | 0.00 | 0.00 | 0.00 | 0.00 | 0.00 |
| TRBV25-1 | 89.73 | 14165.25 | 35.50 | 36.86 | 105.19 | 194.67 | 632.48 | 29921.78 | 52816.28 | 7684.11 | 5710.62 | 247980.47 |
| TRBV12-4 | 33808.03 | 4939.45 | 16221.88 | 48.97 | 39325.09 | 11327.04 | 7570.15 | 55083.48 | 6152.46 | 14877.23 | 9918.25 | 15874.63 |
