# Supplementary material for: The intrarenal landscape of T cell receptor repertoire in clear cell renal cell cancer
Source: J Transl Med. 2022 Dec 3;20:558. doi: 10.1186/s12967-022-03771-3 (PMC9719196; doi:10.1186/s12967-022-03771-3)
Supplement: Supplementary file 3 — Additional file 3. Comparison of TRA/B CDR3 AA usage. [file 12967_2022_3771_MOESM3_ESM.docx]

| Additional file 3. Comparison of TRA/B CDR3 AA usage | | | | | | |
| --- | --- | --- | --- | --- | --- | --- |
| Samples | Pear reads | Blast reads(V&J-AA) | Blast/Pear(%) | CDR3 reads | CDR3/pear(%) | CDR3 variety |
| Peritumour1 | 15254357.00 | 6288441.00 | 41.22 | 6182917.00 | 40.53 | 13435.00 |
| Peritumour2 | 9859046.00 | 688866.00 | 6.99 | 648272.00 | 6.58 | 10218.00 |
| Peritumour3 | 17102886.00 | 6619057.00 | 38.70 | 6495734.00 | 37.98 | 9545.00 |
| Peritumour4 | 22140481.00 | 803074.00 | 3.63 | 787671.00 | 3.56 | 5216.00 |
| Peritumour5 | 16156116.00 | 7399572.00 | 45.80 | 7277785.00 | 45.05 | 17040.00 |
| Peritumour6 | 11903361.00 | 4764754.00 | 40.03 | 4613837.00 | 38.76 | 20285.00 |
| ccRCC1 | 10587489.00 | 1329561.00 | 12.56 | 1235221.00 | 11.67 | 11343.00 |
| ccRCC2 | 11813141.00 | 1814217.00 | 15.36 | 1728038.00 | 14.63 | 16739.00 |
| ccRCC3 | 11888728.00 | 1882403.00 | 15.83 | 1780044.00 | 14.97 | 12992.00 |
| ccRCC4 | 12897729.00 | 3474033.00 | 26.94 | 3356367.00 | 26.02 | 35577.00 |
| ccRCC5 | 14192685.00 | 4277243.00 | 30.14 | 4101307.00 | 28.90 | 30062.00 |
| ccRCC6 | 14943961.00 | 3255082.00 | 21.78 | 3207629.00 | 21.46 | 9352.00 |
