# Supplementary material for: The intrarenal landscape of T cell receptor repertoire in clear cell renal cell cancer
Source: J Transl Med. 2022 Dec 3;20:558. doi: 10.1186/s12967-022-03771-3 (PMC9719196; doi:10.1186/s12967-022-03771-3)
Supplement: Supplementary file 4 — Additional file 4. Comparison of TRA/ BV-J combination usage. [file 12967_2022_3771_MOESM4_ESM.docx]

| Additional file 4. Comparison of TRA/ BV-J combination usage. | | | | | | | | | | | | | | | | |
| --- | --- | --- | --- | --- | --- | --- | --- | --- | --- | --- | --- | --- | --- | --- | --- | --- |
|  | Peri-  tumour1 | Peri-  tumour2 | Peri-  tumour3 | Peri-  tumour4 | Peri-  tumour5 | Peri-  tumour6 | ccRCC1 | ccRCC2 | ccRCC3 | ccRCC4 | ccRCC5 | ccRCC6 | t.test.p | p.adj.fdr | log2FC | sig |
| TRBV19_TRBJ2-5 | 2.82 | 10013.70 | 1.38 | 0.79 | 37.26 | 239.72 | 297.99 | 704.61 | 1041.72 | 5708.52 | 2293.16 | 95.99 | 0.99 | 0.99 | -0.02 | no |
| TRBV19_TRBJ2-1 | 18.51 | 22595.29 | 7.07 | 11.59 | 183.43 | 275.92 | 12717.  14 | 11593.76 | 20382.  90 | 17539.86 | 19056.  65 | 1000.04 | 0.07 | 0.59 | 1.83 | no |
| TRBV19_TRBJ1-3 | 1.57 | 28.79 | 0.77 | 1.18 | 5.91 | 8.25 | 13.51 | 12019.51 | 32.32 | 52.73 | 19.18 | 3.34 | 0.36 | 0.59 | 8.03 | no |
| TRBV19_TRBJ1-5 | 23.06 | 27486.31 | 8.45 | 13.17 | 36.30 | 54.70 | 181.77 | 12422.66 | 3424.39 | 5772.36 | 2905.95 | 64349.  72 | 0.38 | 0.59 | 1.69 | no |
| TRBV19_TRBJ1-6 | 23.84 | 602.05 | 12.76 | 12.51 | 35.61 | 66.97 | 221.64 | 3599.89 | 161.62 | 278.80 | 94.47 | 111261.85 | 0.35 | 0.59 | 7.26 | no |
| TRBV19_TRBJ2-6 | 0.63 | 62.82 | 0.77 | 297.40 | 5.50 | 46.05 | 8.78 | 25.76 | 5855.55 | 160.97 | 878.45 | 2.43 | 0.30 | 0.59 | 4.07 | no |
| TRBV19_TRBJ2-4 | 0.16 | 119.10 | 0.00 | 0.13 | 0.00 | 0.40 | 87.84 | 337.16 | 46.64 | 179.90 | 46.41 | 0.61 | 0.12 | 0.59 | 2.54 | no |
| TRBV19_TRBJ2-2 | 54.28 | 18066.81 | 25.82 | 16.19 | 70561.  77 | 32686.00 | 392.60 | 397.28 | 6325.15 | 8050.21 | 1149.05 | 150.98 | 0.19 | 0.59 | -2.88 | no |
| TRBV19_TRBJ2-3 | 12.86 | 917.48 | 5.99 | 9.08 | 73.70 | 80.04 | 206.77 | 22239.31 | 29932.  04 | 3112.75 | 178.82 | 17771.  43 | 0.07 | 0.59 | 6.06 | no |
| TRBV19_TRBJ1-2 | 536.01 | 12580.28 | 237.29 | 936928.36 | 707.03 | 1134.43 | 3385.38 | 15723.35 | 6970.69 | 18824.62 | 5240.36 | 1630.39 | 0.38 | 0.59 | -4.20 | no |
| TRBV19_TRBJ1-1 | 30.27 | 24146.23 | 11.37 | 12.38 | 65.86 | 87.08 | 6103.15 | 590.26 | 2142.09 | 13514.50 | 4848.26 | 89061.  31 | 0.34 | 0.59 | 2.26 | no |
| TRBV19_TRBJ2-7 | 17.41 | 8072.74 | 5.38 | 7.24 | 43.31 | 20274.00 | 462.20 | 4204.61 | 4194.60 | 31318.78 | 1856.51 | 457.80 | 0.70 | 0.79 | 0.58 | no |
| TRBV19_TRBJ1-4 | 0.63 | 10679.89 | 0.31 | 0.26 | 4.54 | 4.02 | 22.97 | 95.82 | 10.62 | 63.08 | 41.66 | 568.07 | 0.40 | 0.59 | -3.74 | no |
| TRBV12-2_TRBJ2-3 | 0.00 | 0.00 | 0.00 | 0.00 | 0.00 | 0.20 | 0.00 | 0.00 | 0.00 | 0.00 | 0.00 | 0.00 | 0.36 | 0.59 | #NAME? | no |
| TRBV12-2_TRBJ2-5 | 0.00 | 0.00 | 0.00 | 0.00 | 0.14 | 0.00 | 0.00 | 0.00 | 0.00 | 0.00 | 0.00 | 0.00 | 0.36 | 0.59 | #NAME? | no |
| TRBV14_TRBJ1-3 | 0.00 | 0.00 | 0.00 | 0.00 | 0.00 | 0.00 | 0.00 | 0.00 | 0.46 | 0.00 | 0.00 | 0.00 | 0.36 | 0.59 | Inf | no |
| TRBV14_TRBJ1-5 | 0.00 | 0.00 | 0.61 | 0.00 | 0.00 | 0.00 | 0.00 | 0.00 | 4.16 | 0.00 | 0.00 | 0.00 | 0.44 | 0.60 | 2.76 | no |
| TRBV14_TRBJ2-5 | 0.00 | 1.31 | 0.00 | 0.00 | 0.00 | 0.00 | 0.00 | 0.00 | 1.39 | 0.00 | 0.00 | 0.00 | 0.97 | 0.98 | 0.08 | no |
| TRBV14_TRBJ2-1 | 0.00 | 2.62 | 0.00 | 0.00 | 0.41 | 7.24 | 0.00 | 0.00 | 0.92 | 0.00 | 0.00 | 11.54 | 0.87 | 0.91 | 0.28 | no |
| TRBV14_TRBJ2-2 | 0.00 | 0.00 | 0.00 | 0.00 | 0.00 | 0.00 | 0.00 | 0.00 | 0.46 | 0.00 | 0.00 | 0.00 | 0.36 | 0.59 | Inf | no |
| TRBV14_TRBJ2-6 | 0.00 | 1.31 | 0.00 | 0.00 | 0.00 | 0.00 | 0.00 | 0.90 | 0.00 | 0.00 | 0.00 | 0.00 | 0.80 | 0.86 | -0.53 | no |
| TRBV14_TRBJ1-6 | 0.00 | 0.00 | 112.19 | 0.00 | 0.00 | 0.40 | 0.00 | 0.00 | 74.34 | 0.00 | 0.00 | 0.30 | 0.78 | 0.84 | -0.59 | no |
| TRBV14_TRBJ1-2 | 0.00 | 0.00 | 0.00 | 0.00 | 0.00 | 0.00 | 0.00 | 0.00 | 2.77 | 0.00 | 0.00 | 0.00 | 0.36 | 0.59 | Inf | no |
| TRBV14_TRBJ2-3 | 0.00 | 0.00 | 0.00 | 0.00 | 0.00 | 5.63 | 0.00 | 0.00 | 0.00 | 0.00 | 0.00 | 0.30 | 0.39 | 0.59 | -4.21 | no |
| TRBV14_TRBJ2-7 | 0.00 | 0.00 | 0.00 | 0.00 | 6.33 | 0.00 | 0.68 | 0.00 | 0.46 | 0.00 | 0.41 | 0.30 | 0.51 | 0.65 | -1.77 | no |
| TRBV14_TRBJ1-4 | 0.00 | 0.00 | 0.00 | 0.00 | 0.00 | 0.00 | 0.00 | 0.00 | 0.46 | 0.00 | 0.00 | 0.00 | 0.36 | 0.59 | Inf | no |
| TRBV14_TRBJ1-1 | 0.00 | 0.00 | 0.00 | 0.00 | 0.00 | 0.00 | 0.00 | 0.00 | 2.31 | 0.00 | 0.00 | 0.30 | 0.30 | 0.59 | Inf | no |
| TRBV7-1_TRBJ2-1 | 0.00 | 0.00 | 0.00 | 0.00 | 0.00 | 0.00 | 0.00 | 0.45 | 0.00 | 0.25 | 0.00 | 0.00 | 0.20 | 0.59 | Inf | no |
| TRBV7-1_TRBJ2-3 | 0.00 | 0.00 | 0.00 | 0.00 | 0.00 | 0.20 | 0.68 | 0.00 | 0.00 | 0.00 | 0.00 | 0.00 | 0.53 | 0.66 | 1.75 | no |
| TRBV7-1_TRBJ2-7 | 0.00 | 0.00 | 0.00 | 0.00 | 0.00 | 0.00 | 0.00 | 0.00 | 0.00 | 0.25 | 0.00 | 0.00 | 0.36 | 0.59 | Inf | no |
| TRBV11-2_TRBJ2-7 | 2.35 | 22.25 | 0.15 | 0.53 | 13.61 | 35.80 | 32.43 | 5.42 | 6.46 | 527.58 | 45.58 | 2.43 | 0.34 | 0.59 | 3.05 | no |
| TRBV11-2_TRBJ1-4 | 0.16 | 0.00 | 0.15 | 0.00 | 0.14 | 0.00 | 16.22 | 1.36 | 0.46 | 4.29 | 0.41 | 0.00 | 0.21 | 0.59 | 5.67 | no |
| TRBV11-2_TRBJ1-1 | 0.00 | 19.63 | 0.15 | 0.13 | 0.28 | 1.21 | 8.11 | 127.00 | 5.08 | 23.46 | 3.30 | 1.22 | 0.28 | 0.59 | 2.97 | no |
| TRBV11-2_TRBJ1-2 | 1.10 | 32.72 | 0.15 | 0.00 | 19.25 | 35.60 | 7.43 | 155.93 | 0.92 | 60.55 | 5.57 | 2.43 | 0.39 | 0.59 | 1.39 | no |
| TRBV11-2_TRBJ2-3 | 1.57 | 37.96 | 0.46 | 0.53 | 0.14 | 634.49 | 141.90 | 5.88 | 54.03 | 12.87 | 11.96 | 1.52 | 0.51 | 0.65 | -1.57 | no |
| TRBV11-2_TRBJ2-2 | 1.10 | 2.62 | 0.00 | 0.00 | 0.14 | 1.81 | 3.38 | 0.90 | 0.92 | 6.31 | 7.84 | 0.00 | 0.15 | 0.59 | 1.77 | no |
| TRBV11-2_TRBJ2-4 | 0.00 | 0.00 | 0.00 | 0.00 | 0.00 | 0.00 | 2.70 | 0.00 | 0.00 | 1.77 | 1.65 | 0.00 | 0.09 | 0.59 | Inf | no |
| TRBV11-2_TRBJ2-6 | 0.16 | 1.31 | 0.00 | 0.00 | 0.14 | 44.85 | 1.35 | 0.45 | 1.85 | 4.04 | 0.83 | 0.00 | 0.43 | 0.60 | -2.45 | no |
| TRBV11-2_TRBJ1-6 | 0.00 | 0.00 | 0.15 | 0.00 | 0.00 | 0.60 | 0.00 | 0.90 | 0.46 | 2.27 | 3.09 | 0.00 | 0.12 | 0.59 | 3.15 | no |
| TRBV11-2_TRBJ1-3 | 0.00 | 7.85 | 0.00 | 0.13 | 0.00 | 0.20 | 0.00 | 3.62 | 3.69 | 0.25 | 0.00 | 0.00 | 0.95 | 0.96 | -0.11 | no |
| TRBV11-2_TRBJ1-5 | 0.00 | 0.00 | 0.15 | 0.00 | 0.14 | 0.40 | 1.35 | 0.45 | 0.92 | 4.79 | 0.41 | 0.30 | 0.13 | 0.59 | 3.57 | no |
| TRBV11-2_TRBJ2-5 | 1.57 | 6.54 | 0.15 | 0.13 | 3.03 | 2.41 | 116.90 | 36.61 | 9.24 | 36.08 | 9.69 | 0.00 | 0.12 | 0.59 | 3.91 | no |
| TRBV11-2_TRBJ2-1 | 88.00 | 27.49 | 0.46 | 0.00 | 0.83 | 7.04 | 16.22 | 63.27 | 70.19 | 132.46 | 202.96 | 0.91 | 0.12 | 0.59 | 1.97 | no |
| TRBV5-6_TRBJ1-2 | 0.16 | 221.19 | 0.31 | 0.26 | 1552.26 | 27.55 | 2.03 | 21.69 | 3.23 | 1.51 | 48.88 | 0.61 | 0.31 | 0.59 | -4.53 | no |
| TRBV5-6_TRBJ2-3 | 0.00 | 65.44 | 0.00 | 0.13 | 1.38 | 0.40 | 0.00 | 6.33 | 0.00 | 28.26 | 54.25 | 0.00 | 0.81 | 0.86 | 0.40 | no |
| TRBV5-6_TRBJ1-4 | 0.00 | 0.00 | 0.00 | 0.00 | 2.20 | 0.00 | 0.00 | 0.00 | 0.00 | 0.00 | 7.22 | 0.00 | 0.53 | 0.66 | 1.71 | no |
| TRBV5-6_TRBJ2-7 | 0.00 | 0.00 | 0.00 | 0.00 | 1.93 | 0.00 | 0.00 | 4.97 | 0.46 | 10.60 | 59.61 | 0.00 | 0.25 | 0.59 | 5.30 | no |
| TRBV5-6_TRBJ1-1 | 0.00 | 1.31 | 0.00 | 0.00 | 4.26 | 0.20 | 0.00 | 3.62 | 0.00 | 0.00 | 18.36 | 0.00 | 0.42 | 0.59 | 1.93 | no |
| TRBV5-6_TRBJ1-5 | 0.00 | 0.00 | 0.00 | 0.00 | 2.34 | 0.40 | 0.00 | 21.24 | 0.92 | 0.00 | 11.34 | 0.00 | 0.22 | 0.59 | 3.61 | no |
| TRBV5-6_TRBJ1-3 | 0.00 | 1.31 | 0.15 | 0.00 | 0.83 | 0.20 | 0.00 | 230.05 | 0.00 | 0.00 | 4.13 | 0.00 | 0.36 | 0.59 | 6.56 | no |
| TRBV5-6_TRBJ2-5 | 0.16 | 5.24 | 0.00 | 0.00 | 3.44 | 0.00 | 0.00 | 43.39 | 0.00 | 7.82 | 4.95 | 0.00 | 0.31 | 0.59 | 2.67 | no |
| TRBV5-6_TRBJ2-1 | 0.00 | 0.00 | 0.00 | 0.00 | 0.96 | 0.00 | 1.35 | 2.71 | 0.00 | 16.90 | 11.96 | 0.30 | 0.12 | 0.59 | 5.11 | no |
| TRBV5-6_TRBJ2-4 | 0.00 | 0.00 | 0.00 | 0.00 | 0.00 | 0.00 | 0.00 | 0.00 | 0.00 | 8.33 | 0.00 | 0.00 | 0.36 | 0.59 | Inf | no |
| TRBV5-6_TRBJ2-6 | 0.00 | 0.00 | 0.00 | 0.00 | 0.00 | 0.00 | 0.00 | 0.00 | 0.00 | 0.25 | 0.21 | 0.00 | 0.18 | 0.59 | Inf | no |
| TRBV5-6_TRBJ2-2 | 0.31 | 44.50 | 0.00 | 0.00 | 0.00 | 0.00 | 0.00 | 0.45 | 0.00 | 0.25 | 5.16 | 0.00 | 0.42 | 0.60 | -2.93 | no |
| TRBV5-6_TRBJ1-6 | 0.00 | 3.93 | 0.31 | 0.00 | 0.41 | 0.60 | 1.35 | 16.72 | 2.31 | 1.01 | 1246.61 | 0.00 | 0.36 | 0.59 | 7.92 | no |
| TRBV12-4_TRBJ1-1 | 17.41 | 770.89 | 0.61 | 0.39 | 83.05 | 45.25 | 59.46 | 5476.44 | 300.60 | 1253.23 | 2265.31 | 45.26 | 0.16 | 0.59 | 3.36 | no |
| TRBV12-4_TRBJ2-7 | 469.97 | 162.29 | 1.84 | 0.79 | 293.02 | 30.17 | 958.18 | 1361.77 | 82.19 | 1970.80 | 2529.32 | 8806.58 | 0.11 | 0.59 | 4.04 | no |
| TRBV12-4_TRBJ1-4 | 11.92 | 20.94 | 0.00 | 0.39 | 37.68 | 10.86 | 1850.14 | 1876.55 | 872.72 | 1054.91 | 37.95 | 24.61 | 0.04 | 0.59 | 6.13 | up |
| TRBV12-4_TRBJ2-3 | 196.87 | 176.69 | 15690.91 | 3.03 | 58.99 | 47.66 | 142.58 | 535.58 | 79.88 | 183.93 | 62.91 | 2497.68 | 0.46 | 0.61 | -2.21 | no |
| TRBV12-4_TRBJ1-2 | 42.98 | 248.67 | 9.22 | 7.64 | 22425.43 | 10983.55 | 175.01 | 3678.08 | 1579.21 | 365.35 | 2335.44 | 47.39 | 0.32 | 0.59 | -2.04 | no |
| TRBV12-4_TRBJ1-6 | 0.16 | 7.85 | 1.38 | 0.13 | 3.30 | 20.71 | 4.73 | 43.84 | 1063.89 | 49.45 | 418.08 | 24.91 | 0.19 | 0.59 | 5.58 | no |
| TRBV12-4_TRBJ2-2 | 10.82 | 43.19 | 104.04 | 0.39 | 27.91 | 27.15 | 24.33 | 63.73 | 15.24 | 4537.30 | 389.82 | 5.16 | 0.33 | 0.59 | 4.56 | no |
| TRBV12-4_TRBJ2-6 | 0.00 | 2.62 | 99.28 | 1.32 | 71.23 | 13.07 | 0.00 | 34.35 | 13.39 | 71.40 | 12.17 | 0.61 | 0.67 | 0.77 | -0.51 | no |
| TRBV12-4_TRBJ2-4 | 4.08 | 2990.63 | 2.61 | 3.69 | 7.56 | 14.68 | 1054.81 | 33770.70 | 889.34 | 2939.16 | 140.25 | 13.67 | 0.33 | 0.59 | 3.68 | no |
| TRBV12-4_TRBJ2-1 | 539.30 | 214.64 | 109.27 | 26.73 | 16072.85 | 48.27 | 242.59 | 5089.56 | 125.14 | 1834.04 | 1224.34 | 970.27 | 0.67 | 0.76 | -0.84 | no |
| TRBV12-4_TRBJ2-5 | 32513.58 | 280.09 | 176.27 | 4.21 | 167.48 | 51.89 | 1858.92 | 1971.46 | 1053.27 | 555.59 | 389.41 | 128.20 | 0.44 | 0.60 | -2.48 | no |
| TRBV12-4_TRBJ1-5 | 0.94 | 20.94 | 26.43 | 0.26 | 74.66 | 16.29 | 1197.38 | 1106.86 | 74.34 | 52.99 | 98.59 | 3310.29 | 0.12 | 0.59 | 5.39 | no |
| TRBV12-4_TRBJ1-3 | 0.00 | 0.00 | 0.00 | 0.00 | 1.93 | 17.50 | 2.03 | 74.57 | 3.23 | 9.08 | 14.64 | 0.00 | 0.29 | 0.59 | 2.41 | no |
| TRBV11-3_TRBJ2-7 | 1.88 | 37.96 | 0.15 | 0.79 | 49.64 | 41.63 | 209.47 | 16.72 | 14.31 | 529.85 | 80.65 | 2.43 | 0.21 | 0.59 | 2.69 | no |
| TRBV11-3_TRBJ1-4 | 0.16 | 1.31 | 0.00 | 0.00 | 0.28 | 0.00 | 62.17 | 1.36 | 0.00 | 5.80 | 1.44 | 28.86 | 0.17 | 0.59 | 5.84 | no |
| TRBV11-3_TRBJ1-1 | 0.31 | 102.09 | 0.00 | 0.13 | 0.83 | 15.28 | 179.74 | 136.94 | 48.95 | 87.30 | 40.84 | 0.00 | 0.08 | 0.59 | 2.06 | no |
| TRBV11-3_TRBJ1-2 | 0.78 | 48.43 | 0.00 | 0.00 | 70.54 | 58.72 | 21.62 | 268.92 | 21.70 | 61.82 | 27.84 | 26.13 | 0.36 | 0.59 | 1.26 | no |
| TRBV11-3_TRBJ2-3 | 2.35 | 119.10 | 0.15 | 0.39 | 25.16 | 424.13 | 368.95 | 21.24 | 103.43 | 69.13 | 47.03 | 2.13 | 0.94 | 0.96 | 0.10 | no |
| TRBV11-3_TRBJ2-4 | 0.00 | 0.00 | 0.00 | 0.00 | 0.00 | 0.00 | 1.35 | 3.16 | 0.00 | 4.04 | 5.57 | 0.30 | 0.05 | 0.59 | Inf | up |
| TRBV11-3_TRBJ2-6 | 0.16 | 2.62 | 0.00 | 2.76 | 0.28 | 25.74 | 2.03 | 0.00 | 3.23 | 8.07 | 0.62 | 0.30 | 0.53 | 0.66 | -1.15 | no |
| TRBV11-3_TRBJ2-2 | 3.61 | 3.93 | 0.00 | 0.00 | 0.14 | 5.23 | 215.56 | 10.85 | 4.62 | 32.04 | 16.71 | 0.30 | 0.25 | 0.59 | 4.44 | no |
| TRBV11-3_TRBJ1-6 | 0.16 | 7.85 | 0.00 | 0.00 | 0.41 | 0.20 | 2.03 | 3.62 | 8.77 | 7.32 | 31.35 | 1.52 | 0.16 | 0.59 | 2.66 | no |
| TRBV11-3_TRBJ1-3 | 0.00 | 3.93 | 0.00 | 0.00 | 0.00 | 0.00 | 0.00 | 23.95 | 18.01 | 0.00 | 0.41 | 0.00 | 0.21 | 0.59 | 3.43 | no |
| TRBV11-3_TRBJ1-5 | 0.00 | 2.62 | 0.00 | 0.00 | 0.14 | 7.44 | 18.92 | 3.62 | 10.16 | 17.16 | 6.39 | 0.00 | 0.06 | 0.59 | 2.46 | no |
| TRBV11-3_TRBJ2-1 | 43.29 | 138.73 | 16.91 | 0.79 | 4.40 | 24.53 | 149.34 | 404.06 | 159.31 | 195.29 | 163.77 | 2.13 | 0.04 | 0.59 | 2.23 | up |
| TRBV11-3_TRBJ2-5 | 14.43 | 37.96 | 0.15 | 0.39 | 176.14 | 9.85 | 356.78 | 245.42 | 26.78 | 147.85 | 37.54 | 0.91 | 0.18 | 0.59 | 1.77 | no |
| TRBV5-3_TRBJ2-3 | 0.00 | 0.00 | 0.00 | 0.00 | 0.00 | 0.00 | 0.00 | 0.45 | 0.00 | 0.00 | 0.83 | 0.00 | 0.20 | 0.59 | Inf | no |
| TRBV5-3_TRBJ1-1 | 0.00 | 0.00 | 0.00 | 0.00 | 0.00 | 0.00 | 0.00 | 0.45 | 0.00 | 0.00 | 0.62 | 0.00 | 0.18 | 0.59 | Inf | no |
| TRBV5-3_TRBJ2-7 | 0.00 | 0.00 | 0.00 | 0.00 | 0.14 | 0.20 | 0.00 | 0.00 | 0.46 | 0.00 | 0.62 | 0.00 | 0.35 | 0.59 | 1.67 | no |
| TRBV5-3_TRBJ2-1 | 0.00 | 0.00 | 0.00 | 0.00 | 0.00 | 0.20 | 0.00 | 0.00 | 0.00 | 0.00 | 0.00 | 0.00 | 0.36 | 0.59 | #NAME? | no |
| TRBV5-3_TRBJ1-5 | 0.00 | 0.00 | 0.00 | 0.00 | 0.00 | 0.00 | 0.00 | 0.00 | 0.00 | 0.00 | 0.41 | 0.00 | 0.36 | 0.59 | Inf | no |
| TRBV5-3_TRBJ1-3 | 0.00 | 0.00 | 0.00 | 0.00 | 0.00 | 0.00 | 0.00 | 0.00 | 0.00 | 0.00 | 0.21 | 0.00 | 0.36 | 0.59 | Inf | no |
| TRBV5-3_TRBJ2-6 | 0.00 | 0.00 | 0.00 | 0.00 | 0.00 | 0.00 | 0.00 | 0.00 | 0.00 | 0.25 | 0.62 | 0.00 | 0.22 | 0.59 | Inf | no |
| TRBV5-3_TRBJ2-2 | 0.31 | 1.31 | 0.00 | 0.00 | 0.14 | 0.40 | 0.68 | 1.36 | 0.46 | 0.25 | 191.20 | 0.00 | 0.36 | 0.59 | 6.49 | no |
| TRBV5-3_TRBJ1-6 | 0.00 | 0.00 | 0.00 | 0.00 | 0.00 | 0.00 | 0.00 | 0.45 | 0.00 | 0.00 | 0.41 | 0.00 | 0.18 | 0.59 | Inf | no |
| TRBV6-4_TRBJ1-6 | 0.00 | 0.00 | 0.00 | 0.00 | 0.00 | 0.60 | 0.00 | 0.00 | 0.00 | 34.31 | 3.09 | 0.30 | 0.32 | 0.59 | 5.97 | no |
| TRBV6-4_TRBJ2-2 | 144.94 | 2.62 | 1.38 | 0.13 | 0.69 | 0.60 | 0.00 | 1.36 | 0.46 | 68.88 | 14.85 | 0.61 | 0.70 | 0.79 | -0.80 | no |
| TRBV6-4_TRBJ2-4 | 0.00 | 1.31 | 0.00 | 0.00 | 0.00 | 0.00 | 0.68 | 0.00 | 0.00 | 33.05 | 11.55 | 0.00 | 0.24 | 0.59 | 5.11 | no |
| TRBV6-4_TRBJ2-6 | 0.47 | 5.24 | 3.23 | 0.13 | 445.51 | 0.80 | 3.38 | 4.97 | 1.85 | 48.44 | 31.14 | 1.52 | 0.45 | 0.61 | -2.32 | no |
| TRBV6-4_TRBJ2-1 | 89217.  79 | 835.02 | 21.67 | 13.17 | 88.83 | 183.01 | 479.76 | 391.85 | 279.83 | 3807.87 | 1951.19 | 144.90 | 0.39 | 0.59 | -3.68 | no |
| TRBV6-4_TRBJ2-5 | 674.36 | 9.16 | 0.46 | 0.00 | 6.60 | 2.21 | 3.38 | 2.26 | 0.46 | 142.30 | 52.60 | 0.30 | 0.50 | 0.64 | -1.78 | no |
| TRBV6-4_TRBJ1-3 | 0.00 | 0.00 | 0.00 | 0.00 | 0.00 | 0.00 | 0.00 | 0.00 | 0.00 | 13.88 | 16.50 | 0.00 | 0.18 | 0.59 | Inf | no |
| TRBV6-4_TRBJ1-5 | 0.00 | 0.00 | 0.00 | 0.00 | 0.28 | 0.00 | 0.00 | 0.00 | 0.46 | 17.66 | 13.82 | 0.00 | 0.17 | 0.59 | 6.86 | no |
| TRBV6-4_TRBJ1-1 | 322.99 | 9.16 | 0.00 | 0.13 | 1.38 | 0.00 | 4.05 | 11.30 | 0.92 | 44.41 | 13.41 | 0.61 | 0.46 | 0.61 | -2.16 | no |
| TRBV6-4_TRBJ2-7 | 1221.51 | 20.94 | 0.31 | 0.39 | 229.35 | 3.02 | 4.05 | 53.78 | 5.54 | 184.19 | 64.35 | 3.95 | 0.38 | 0.59 | -2.22 | no |
| TRBV6-4_TRBJ1-4 | 256.32 | 1.31 | 0.31 | 0.00 | 0.69 | 0.40 | 2.70 | 4.07 | 0.46 | 3.28 | 6.60 | 0.00 | 0.39 | 0.59 | -3.92 | no |
| TRBV6-4_TRBJ2-3 | 592.95 | 18.32 | 0.61 | 0.39 | 6.60 | 3.42 | 11.49 | 9.94 | 11.54 | 3175.58 | 1014.78 | 3.34 | 0.31 | 0.59 | 2.76 | no |
| TRBV6-4_TRBJ1-2 | 88.16 | 0.00 | 0.00 | 0.00 | 0.28 | 0.80 | 370.97 | 0.00 | 0.46 | 56.52 | 9.08 | 0.00 | 0.39 | 0.59 | 2.29 | no |
| TRBV6-8_TRBJ2-5 | 17.88 | 3571.74 | 1415.73 | 0.79 | 51.15 | 325.59 | 62.84 | 3294.36 | 1322.94 | 464.76 | 1890.13 | 5.47 | 0.73 | 0.81 | 0.39 | no |
| TRBV6-8_TRBJ2-1 | 237.96 | 1150.44 | 101080.09 | 20.54 | 1598.32 | 9128.16 | 612.21 | 1931.24 | 414.66 | 7193.62 | 2268.82 | 203.23 | 0.36 | 0.59 | -3.16 | no |
| TRBV6-8_TRBJ1-5 | 4.24 | 310.19 | 976.35 | 1.32 | 280.37 | 70.19 | 2096.10 | 247.68 | 15690.52 | 729.18 | 1846.61 | 22.18 | 0.26 | 0.59 | 3.65 | no |
| TRBV6-8_TRBJ1-3 | 0.63 | 10.47 | 0.46 | 1.45 | 1.51 | 1043.74 | 9.46 | 810.37 | 41.10 | 687.29 | 88.07 | 2.73 | 0.68 | 0.78 | 0.63 | no |
| TRBV6-8_TRBJ1-6 | 1.57 | 87.69 | 8.15 | 0.39 | 4.68 | 88.29 | 333.13 | 323.15 | 42.02 | 5223.58 | 222.76 | 13.67 | 0.29 | 0.59 | 5.01 | no |
| TRBV6-8_TRBJ2-6 | 44.08 | 687.13 | 66461.70 | 13.96 | 2157.81 | 183.21 | 258.13 | 709.58 | 229.03 | 4331.66 | 190.17 | 103.89 | 0.38 | 0.59 | -3.58 | no |
| TRBV6-8_TRBJ2-4 | 0.16 | 47.12 | 0.31 | 0.00 | 0.41 | 0.00 | 8.78 | 122.93 | 7.39 | 71.15 | 230.39 | 0.30 | 0.14 | 0.59 | 3.20 | no |
| TRBV6-8_TRBJ2-2 | 9.10 | 6807.12 | 5786.64 | 2.76 | 14.44 | 7344.76 | 801.41 | 475.01 | 551.80 | 2693.67 | 1378.00 | 17.62 | 0.18 | 0.59 | -1.75 | no |
| TRBV6-8_TRBJ2-3 | 8.31 | 274.85 | 657.46 | 2.90 | 64.08 | 269.48 | 84.47 | 3191.31 | 3205.52 | 5164.79 | 1310.55 | 18.23 | 0.07 | 0.59 | 3.34 | no |
| TRBV6-8_TRBJ1-2 | 20.39 | 13601.15 | 0.77 | 0.66 | 116.19 | 69.98 | 1094.00 | 1652.83 | 109.90 | 1550.19 | 305.88 | 6.08 | 0.53 | 0.66 | -1.55 | no |
| TRBV6-8_TRBJ1-1 | 21014.27 | 12305.43 | 7.53 | 5.00 | 64.90 | 96.13 | 15778.  85 | 1728.31 | 357.40 | 6961.49 | 1113.78 | 1126.42 | 0.81 | 0.86 | -0.31 | no |
| TRBV6-8_TRBJ2-7 | 119.53 | 9984.91 | 3.53 | 0.92 | 2791.70 | 314.13 | 1154.81 | 556.37 | 1138.69 | 2872.55 | 508.83 | 27.34 | 0.51 | 0.65 | -1.08 | no |
| TRBV6-8_TRBJ1-4 | 189.02 | 130.88 | 1.23 | 0.53 | 9.35 | 3521.75 | 75.01 | 1527.18 | 36.48 | 56.27 | 2070.81 | 9.72 | 0.99 | 0.99 | -0.03 | no |
| TRBV26_TRBJ1-6 | 0.00 | 0.00 | 0.00 | 0.00 | 0.00 | 0.20 | 0.00 | 0.00 | 0.00 | 0.25 | 0.00 | 0.00 | 0.88 | 0.91 | 0.33 | no |
| TRBV26_TRBJ2-2 | 0.31 | 0.00 | 0.00 | 0.00 | 0.00 | 0.00 | 0.00 | 0.00 | 0.00 | 0.00 | 0.21 | 0.00 | 0.78 | 0.84 | -0.61 | no |
| TRBV26_TRBJ2-6 | 0.00 | 0.00 | 0.00 | 0.00 | 0.00 | 3.02 | 0.00 | 0.00 | 0.00 | 0.00 | 0.00 | 0.00 | 0.36 | 0.59 | #NAME? | no |
| TRBV26_TRBJ1-5 | 0.00 | 1.31 | 3.53 | 0.00 | 0.55 | 0.60 | 0.00 | 0.00 | 0.00 | 0.25 | 0.83 | 0.00 | 0.20 | 0.59 | -2.48 | no |
| TRBV26_TRBJ2-1 | 0.31 | 1.31 | 0.00 | 0.00 | 0.00 | 0.20 | 0.00 | 0.45 | 0.00 | 2.02 | 0.21 | 0.00 | 0.72 | 0.80 | 0.55 | no |
| TRBV26_TRBJ2-5 | 0.47 | 0.00 | 0.00 | 0.00 | 0.00 | 0.00 | 1.35 | 0.00 | 0.00 | 0.25 | 1.65 | 0.00 | 0.20 | 0.59 | 2.79 | no |
| TRBV26_TRBJ1-4 | 3.29 | 0.00 | 0.00 | 0.00 | 0.00 | 0.00 | 0.00 | 0.00 | 0.00 | 0.25 | 0.00 | 0.00 | 0.40 | 0.59 | -3.71 | no |
| TRBV26_TRBJ2-7 | 2.35 | 0.00 | 0.00 | 0.00 | 1.10 | 4.83 | 0.00 | 0.00 | 0.00 | 3.78 | 2.48 | 0.00 | 0.75 | 0.82 | -0.40 | no |
| TRBV26_TRBJ1-1 | 0.00 | 0.00 | 0.00 | 0.00 | 0.00 | 0.00 | 1.35 | 0.00 | 0.00 | 0.00 | 0.00 | 0.00 | 0.36 | 0.59 | Inf | no |
| TRBV26_TRBJ1-2 | 0.78 | 0.00 | 0.00 | 0.00 | 0.14 | 0.60 | 0.00 | 0.00 | 0.46 | 0.00 | 0.21 | 0.00 | 0.41 | 0.59 | -1.19 | no |
| TRBV26_TRBJ2-3 | 0.00 | 0.00 | 0.00 | 0.00 | 0.00 | 1.01 | 0.00 | 0.00 | 0.00 | 0.25 | 0.41 | 0.00 | 0.77 | 0.83 | -0.60 | no |
| TRBV24-1_TRBJ2-3 | 0.31 | 7.85 | 14.14 | 0.00 | 5.36 | 2.41 | 664.91 | 112.09 | 5.08 | 664.08 | 5.16 | 50.73 | 0.12 | 0.59 | 5.64 | no |
| TRBV24-1_TRBJ1-2 | 0.00 | 2.62 | 0.00 | 0.00 | 0.55 | 0.20 | 3.38 | 0.90 | 0.46 | 2.27 | 69.10 | 0.91 | 0.33 | 0.59 | 4.51 | no |
| TRBV24-1_TRBJ1-1 | 0.00 | 18.32 | 0.00 | 0.00 | 0.28 | 0.20 | 3.38 | 2.26 | 0.00 | 5.80 | 57.75 | 0.61 | 0.42 | 0.59 | 1.89 | no |
| TRBV24-1_TRBJ1-4 | 0.00 | 688.43 | 0.15 | 0.00 | 0.00 | 0.40 | 0.68 | 111.63 | 0.46 | 0.76 | 0.83 | 0.00 | 0.45 | 0.60 | -2.59 | no |
| TRBV24-1_TRBJ2-7 | 0.00 | 1.31 | 0.15 | 0.13 | 19.39 | 3.62 | 35.14 | 10.40 | 6.00 | 317.66 | 5.78 | 54.07 | 0.23 | 0.59 | 4.12 | no |
| TRBV24-1_TRBJ2-1 | 12.39 | 149.20 | 12116.85 | 2.90 | 4829.75 | 517.04 | 996.69 | 63.27 | 402.65 | 689.82 | 82.50 | 2936.34 | 0.35 | 0.59 | -1.77 | no |
| TRBV24-1_TRBJ2-5 | 0.16 | 5.24 | 88.06 | 0.26 | 25.71 | 5.63 | 457.47 | 165.87 | 8.31 | 413.79 | 139.64 | 17.92 | 0.07 | 0.59 | 3.27 | no |
| TRBV24-1_TRBJ1-5 | 0.16 | 9.16 | 2.92 | 0.00 | 1.10 | 0.60 | 0.68 | 150.50 | 0.46 | 142.81 | 67.24 | 0.61 | 0.10 | 0.59 | 4.70 | no |
| TRBV24-1_TRBJ1-3 | 0.00 | 1.31 | 0.00 | 0.00 | 0.00 | 0.20 | 0.00 | 1.36 | 0.00 | 0.50 | 42.08 | 0.00 | 0.36 | 0.59 | 4.86 | no |
| TRBV24-1_TRBJ1-6 | 0.00 | 0.00 | 0.31 | 0.00 | 0.00 | 0.20 | 0.00 | 0.00 | 0.00 | 2.78 | 0.83 | 0.91 | 0.19 | 0.59 | 3.15 | no |
| TRBV24-1_TRBJ2-6 | 0.00 | 1.31 | 3.07 | 0.00 | 0.83 | 1.21 | 0.00 | 0.00 | 0.92 | 4.54 | 0.00 | 0.00 | 0.86 | 0.90 | -0.23 | no |
| TRBV24-1_TRBJ2-4 | 0.00 | 0.00 | 0.00 | 0.00 | 0.00 | 0.00 | 12.84 | 5.88 | 0.00 | 127.42 | 1.03 | 0.00 | 0.29 | 0.59 | Inf | no |
| TRBV24-1_TRBJ2-2 | 0.16 | 338.98 | 12.14 | 0.00 | 0.69 | 0.20 | 6.76 | 2.26 | 0.00 | 230.86 | 46.82 | 1.52 | 0.88 | 0.91 | -0.29 | no |
| TRBV7-6_TRBJ1-3 | 0.00 | 1.31 | 0.00 | 0.00 | 0.00 | 2.82 | 0.00 | 2.26 | 0.46 | 0.00 | 1.65 | 0.00 | 0.95 | 0.96 | 0.08 | no |
| TRBV7-6_TRBJ1-5 | 0.47 | 10.47 | 0.15 | 0.00 | 0.55 | 21.12 | 580.45 | 4.07 | 1.85 | 6.31 | 650.33 | 1.22 | 0.18 | 0.59 | 5.25 | no |
| TRBV7-6_TRBJ2-5 | 0.00 | 47.12 | 0.15 | 0.00 | 4.95 | 11.26 | 36.49 | 10.40 | 1.85 | 329.26 | 34.65 | 0.30 | 0.32 | 0.59 | 2.70 | no |
| TRBV7-6_TRBJ2-1 | 0.63 | 36.65 | 0.92 | 0.00 | 0.96 | 25.14 | 825.74 | 164.51 | 5.08 | 548.27 | 1190.72 | 1.52 | 0.07 | 0.59 | 5.41 | no |
| TRBV7-6_TRBJ2-6 | 0.00 | 5.24 | 0.00 | 0.00 | 0.00 | 9.05 | 0.00 | 0.45 | 1.39 | 3.53 | 3.71 | 0.00 | 0.63 | 0.74 | -0.65 | no |
| TRBV7-6_TRBJ2-4 | 0.00 | 7.85 | 0.00 | 0.00 | 0.00 | 0.00 | 8.11 | 3.16 | 0.00 | 2.02 | 7.01 | 0.00 | 0.31 | 0.59 | 1.37 | no |
| TRBV7-6_TRBJ2-2 | 0.31 | 496.04 | 0.00 | 0.00 | 0.00 | 9.05 | 9.46 | 4.97 | 0.00 | 5.80 | 6.81 | 0.30 | 0.38 | 0.59 | -4.21 | no |
| TRBV7-6_TRBJ1-6 | 0.16 | 7.85 | 0.00 | 0.00 | 0.00 | 3.42 | 0.68 | 0.90 | 0.46 | 3.03 | 7.84 | 0.00 | 0.89 | 0.92 | 0.18 | no |
| TRBV7-6_TRBJ1-2 | 0.78 | 153.13 | 0.61 | 0.13 | 5.09 | 3399.28 | 21.62 | 10.85 | 9.24 | 186.96 | 9.28 | 2.73 | 0.37 | 0.59 | -3.89 | no |
| TRBV7-6_TRBJ2-3 | 0.00 | 1156.99 | 0.00 | 0.00 | 2.20 | 16.69 | 689.24 | 189.82 | 2.31 | 14.89 | 14.44 | 0.30 | 0.85 | 0.89 | -0.37 | no |
| TRBV7-6_TRBJ2-7 | 0.31 | 450.23 | 0.15 | 0.00 | 4.40 | 9.45 | 23.65 | 7.68 | 4.16 | 238.69 | 911.03 | 0.00 | 0.49 | 0.63 | 1.35 | no |
| TRBV7-6_TRBJ1-4 | 0.16 | 5.24 | 0.00 | 0.00 | 0.00 | 2.01 | 5.41 | 0.00 | 0.00 | 0.00 | 6.39 | 2.73 | 0.44 | 0.60 | 0.97 | no |
| TRBV7-6_TRBJ1-1 | 0.00 | 1270.85 | 0.00 | 0.13 | 0.14 | 19.51 | 16.22 | 3.16 | 2.77 | 5.80 | 10.11 | 0.00 | 0.37 | 0.59 | -5.08 | no |
| TRBV7-9_TRBJ1-6 | 13.18 | 13907.41 | 4.46 | 6.32 | 47.16 | 159.48 | 143.93 | 4601.89 | 5657.91 | 6940.81 | 22556.  82 | 311.37 | 0.32 | 0.59 | 1.51 | no |
| TRBV7-9_TRBJ2-2 | 817.74 | 3181.72 | 31.20 | 4.08 | 30.80 | 8614.33 | 10918.  36 | 11819.29 | 187.94 | 9696.03 | 7185.97 | 34.63 | 0.11 | 0.59 | 1.65 | no |
| TRBV7-9_TRBJ2-6 | 3.29 | 353.38 | 2.61 | 30.41 | 38.23 | 715.33 | 25.68 | 59.66 | 7850.34 | 4179.52 | 84.36 | 7.29 | 0.23 | 0.59 | 3.42 | no |
| TRBV7-9_TRBJ2-4 | 2.20 | 140.04 | 0.61 | 0.13 | 3.30 | 5.03 | 781.14 | 776.47 | 48.48 | 425.14 | 3163.77 | 2.43 | 0.14 | 0.59 | 5.10 | no |
| TRBV7-9_TRBJ2-5 | 114.51 | 32557.95 | 51.33 | 17.25 | 40400.  21 | 12633.22 | 24856.  54 | 23451.47 | 1371.42 | 14608.26 | 25106.  56 | 136.40 | 0.94 | 0.96 | 0.06 | no |
| TRBV7-9_TRBJ2-1 | 1204.25 | 4671.14 | 1862.03 | 17.51 | 1175.50 | 19440.82 | 82779.  69 | 37804.47 | 17687.  62 | 25865.59 | 5258.51 | 204.44 | 0.11 | 0.59 | 2.58 | no |
| TRBV7-9_TRBJ1-5 | 21.33 | 571.95 | 7.84 | 6.06 | 145.48 | 16662.36 | 22707.  06 | 1129.00 | 10340.  60 | 15124.74 | 5555.72 | 164.34 | 0.20 | 0.59 | 1.66 | no |
| TRBV7-9_TRBJ1-3 | 5.80 | 117.79 | 0.31 | 4.61 | 56.65 | 105.38 | 52.71 | 28671.20 | 274.28 | 105.47 | 86.42 | 12.15 | 0.36 | 0.59 | 6.65 | no |
| TRBV7-9_TRBJ1-1 | 31.84 | 25237.78 | 10.76 | 5.00 | 85.66 | 29850.42 | 32540.  21 | 2880.81 | 11340.  30 | 13916.93 | 4181.02 | 404.33 | 0.83 | 0.88 | 0.24 | no |
| TRBV7-9_TRBJ2-7 | 53.96 | 1942.27 | 21.82 | 21.99 | 44388.  86 | 986.42 | 3237.40 | 1442.21 | 6731.04 | 6269.41 | 24424.  88 | 135.79 | 0.92 | 0.94 | -0.17 | no |
| TRBV7-9_TRBJ1-4 | 10.67 | 387.41 | 3.69 | 2.50 | 41.94 | 82.86 | 650.05 | 203.38 | 102.05 | 211.94 | 268.96 | 31518.  69 | 0.35 | 0.59 | 5.96 | no |
| TRBV7-9_TRBJ2-3 | 46.59 | 14779.08 | 20.90 | 14.35 | 21258.  18 | 2139.56 | 65156.  11 | 8714.30 | 11513.  00 | 23414.40 | 2504.78 | 187.74 | 0.29 | 0.59 | 1.54 | no |
| TRBV7-9_TRBJ1-2 | 56.16 | 9419.50 | 13.37 | 18.69 | 18477.  48 | 23333.01 | 23755.  11 | 13929.51 | 11312.  13 | 13219.30 | 1450.40 | 681.99 | 0.70 | 0.79 | 0.33 | no |
| TRBV11-1_TRBJ1-3 | 0.00 | 0.00 | 0.00 | 0.00 | 0.28 | 0.20 | 1.35 | 5.42 | 0.00 | 0.00 | 0.00 | 0.00 | 0.29 | 0.59 | 3.83 | no |
| TRBV11-1_TRBJ1-5 | 0.00 | 0.00 | 0.00 | 0.13 | 0.14 | 2.21 | 2.70 | 0.45 | 1.39 | 0.76 | 1.86 | 0.00 | 0.18 | 0.59 | 1.53 | no |
| TRBV11-1_TRBJ2-5 | 0.16 | 1.31 | 0.00 | 0.39 | 62.29 | 2.82 | 14.19 | 10.85 | 1.85 | 12.87 | 3.30 | 0.00 | 0.72 | 0.80 | -0.64 | no |
| TRBV11-1_TRBJ2-1 | 0.00 | 2.62 | 0.00 | 0.00 | 0.96 | 0.80 | 10.81 | 8.59 | 2.31 | 7.57 | 4.33 | 0.00 | 0.03 | 0.59 | 2.94 | up |
| TRBV11-1_TRBJ2-2 | 0.00 | 0.00 | 0.00 | 0.00 | 0.00 | 0.20 | 2.03 | 2.71 | 0.00 | 1.77 | 3.92 | 0.00 | 0.04 | 0.59 | 5.70 | up |
| TRBV11-1_TRBJ2-4 | 0.00 | 0.00 | 0.00 | 0.13 | 0.00 | 0.00 | 0.00 | 0.00 | 0.00 | 0.00 | 0.41 | 0.00 | 0.54 | 0.66 | 1.65 | no |
| TRBV11-1_TRBJ2-6 | 0.00 | 1.31 | 0.00 | 0.00 | 0.00 | 0.00 | 0.00 | 0.00 | 0.00 | 0.50 | 0.00 | 0.00 | 0.59 | 0.70 | -1.37 | no |
| TRBV11-1_TRBJ1-6 | 0.16 | 3.93 | 0.00 | 0.13 | 0.28 | 0.40 | 2.03 | 2.26 | 3.69 | 2.27 | 11.76 | 0.30 | 0.15 | 0.59 | 2.19 | no |
| TRBV11-1_TRBJ1-2 | 0.16 | 3.93 | 0.00 | 0.00 | 3.99 | 6.03 | 2.03 | 0.90 | 10.16 | 1.26 | 0.41 | 0.30 | 0.93 | 0.96 | 0.10 | no |
| TRBV11-1_TRBJ2-3 | 0.00 | 5.24 | 0.00 | 0.26 | 3.44 | 1.61 | 17.57 | 0.45 | 4.16 | 5.80 | 0.83 | 0.00 | 0.33 | 0.59 | 1.45 | no |
| TRBV11-1_TRBJ2-7 | 0.00 | 1.31 | 0.00 | 0.00 | 2.89 | 0.40 | 3.38 | 1.36 | 0.46 | 1.26 | 3.30 | 0.00 | 0.28 | 0.59 | 1.09 | no |
| TRBV11-1_TRBJ1-4 | 0.00 | 1.31 | 0.00 | 0.13 | 0.00 | 0.20 | 0.68 | 0.45 | 0.00 | 0.25 | 0.21 | 5.47 | 0.35 | 0.59 | 2.10 | no |
| TRBV11-1_TRBJ1-1 | 0.00 | 3.93 | 0.00 | 0.13 | 0.14 | 2.21 | 9.46 | 0.90 | 3.69 | 3.28 | 2.68 | 0.00 | 0.18 | 0.59 | 1.64 | no |
| TRBV2_TRBJ1-5 | 0.94 | 39.26 | 0.00 | 0.79 | 0.69 | 5.83 | 1050.75 | 567.21 | 434.05 | 276.78 | 259.26 | 1747.65 | 0.03 | 0.59 | 6.51 | up |
| TRBV2_TRBJ1-3 | 0.00 | 1.31 | 0.00 | 0.00 | 0.14 | 2.41 | 0.00 | 14.91 | 1.39 | 6.06 | 124.37 | 0.00 | 0.29 | 0.59 | 5.25 | no |
| TRBV2_TRBJ2-1 | 19688.13 | 4054.69 | 6.45 | 5.00 | 22.28 | 6718.32 | 4127.33 | 1911.35 | 1037.11 | 2877.09 | 351.87 | 56.50 | 0.34 | 0.59 | -1.56 | no |
| TRBV2_TRBJ2-5 | 156.39 | 2680.44 | 0.15 | 0.13 | 1.10 | 59.73 | 89.20 | 736.70 | 473.30 | 449.62 | 179.03 | 1.82 | 0.74 | 0.81 | -0.59 | no |
| TRBV2_TRBJ2-4 | 0.16 | 17.01 | 0.15 | 0.00 | 0.41 | 0.20 | 34.46 | 695.57 | 5.54 | 367.36 | 8.46 | 0.30 | 0.18 | 0.59 | 5.95 | no |
| TRBV2_TRBJ2-6 | 0.00 | 2.62 | 0.00 | 0.00 | 0.14 | 18.50 | 0.00 | 4.97 | 9.24 | 28.51 | 7.22 | 0.00 | 0.39 | 0.59 | 1.23 | no |
| TRBV2_TRBJ2-2 | 45.65 | 137.43 | 0.15 | 0.53 | 1.93 | 3228.34 | 32.43 | 18.53 | 15.70 | 1039.01 | 13.82 | 1.82 | 0.52 | 0.65 | -1.61 | no |
| TRBV2_TRBJ1-6 | 0.94 | 1418.75 | 0.31 | 0.26 | 0.83 | 9.45 | 863.58 | 655.35 | 3.23 | 1016.05 | 184.60 | 11.85 | 0.48 | 0.63 | 0.93 | no |
| TRBV2_TRBJ1-2 | 51904.77 | 544.46 | 9.68 | 9.61 | 54.59 | 90.70 | 320.29 | 854.21 | 149.15 | 607.82 | 154.07 | 78.98 | 0.37 | 0.59 | -4.60 | no |
| TRBV2_TRBJ2-3 | 31598.90 | 2761.59 | 5.99 | 6.06 | 32.59 | 5342.36 | 219.61 | 287.90 | 150.53 | 998.90 | 301.75 | 50.12 | 0.27 | 0.59 | -4.31 | no |
| TRBV2_TRBJ1-4 | 133.65 | 10.47 | 0.00 | 0.13 | 0.14 | 2.41 | 19.60 | 9.94 | 0.00 | 314.38 | 8.87 | 5.47 | 0.55 | 0.67 | 1.29 | no |
| TRBV2_TRBJ2-7 | 223.06 | 226.42 | 0.31 | 0.92 | 2.34 | 71.19 | 1244.01 | 2246.26 | 594.74 | 1304.95 | 2948.85 | 18.23 | 0.03 | 0.59 | 3.99 | up |
| TRBV2_TRBJ1-1 | 147.92 | 75.91 | 0.00 | 0.00 | 0.96 | 7.24 | 54.06 | 538.29 | 12.47 | 503.36 | 147.06 | 10.63 | 0.15 | 0.59 | 2.45 | no |
| TRBV7-3_TRBJ2-4 | 0.00 | 0.00 | 0.00 | 0.00 | 0.00 | 0.00 | 5.41 | 1.81 | 0.92 | 4.29 | 2.68 | 0.00 | 0.03 | 0.59 | Inf | up |
| TRBV7-3_TRBJ2-6 | 0.16 | 3.93 | 32.73 | 3.55 | 0.28 | 21.52 | 0.00 | 0.90 | 1.85 | 5.55 | 1.65 | 0.00 | 0.18 | 0.59 | -2.64 | no |
| TRBV7-3_TRBJ2-2 | 1.41 | 5.24 | 212.70 | 0.00 | 1.65 | 18.90 | 183.80 | 2.26 | 1.39 | 173.59 | 68.68 | 0.00 | 0.54 | 0.66 | 0.84 | no |
| TRBV7-3_TRBJ1-6 | 0.00 | 5.24 | 16.60 | 0.00 | 0.00 | 1.81 | 0.00 | 1.36 | 1.39 | 7.57 | 7.63 | 0.91 | 0.80 | 0.86 | -0.33 | no |
| TRBV7-3_TRBJ2-5 | 6.12 | 158.37 | 589.53 | 0.39 | 14.03 | 349.32 | 29.73 | 24.41 | 21.24 | 45.42 | 122.72 | 0.61 | 0.20 | 0.59 | -2.19 | no |
| TRBV7-3_TRBJ2-1 | 68.86 | 315.42 | 43743.63 | 6.45 | 34.38 | 126.90 | 416.92 | 197.51 | 173.62 | 512.44 | 254.93 | 43.44 | 0.37 | 0.59 | -4.79 | no |
| TRBV7-3_TRBJ1-5 | 0.16 | 382.17 | 96.97 | 0.13 | 0.69 | 18.70 | 68.92 | 10.85 | 68.34 | 154.92 | 6.60 | 0.30 | 0.65 | 0.75 | -0.69 | no |
| TRBV7-3_TRBJ1-3 | 0.00 | 0.00 | 0.15 | 0.00 | 0.14 | 0.00 | 0.00 | 6.33 | 4.62 | 0.76 | 0.21 | 0.00 | 0.15 | 0.59 | 5.35 | no |
| TRBV7-3_TRBJ1-1 | 0.31 | 26.18 | 0.31 | 0.00 | 3.30 | 161.69 | 16.89 | 13.56 | 6.93 | 96.13 | 50.53 | 1.22 | 0.97 | 0.98 | -0.05 | no |
| TRBV7-3_TRBJ1-4 | 0.16 | 11.78 | 0.00 | 0.00 | 0.14 | 0.80 | 16.89 | 0.00 | 0.92 | 6.56 | 2.06 | 17.62 | 0.21 | 0.59 | 1.77 | no |
| TRBV7-3_TRBJ2-7 | 0.94 | 14.40 | 0.15 | 0.00 | 71.09 | 32.98 | 35.81 | 7.23 | 14.78 | 148.11 | 47.23 | 1.82 | 0.40 | 0.59 | 1.09 | no |
| TRBV7-3_TRBJ2-3 | 0.78 | 240.82 | 117.88 | 0.13 | 274.87 | 39.42 | 129.06 | 8.14 | 88.20 | 26.49 | 15.68 | 2.73 | 0.25 | 0.59 | -1.32 | no |
| TRBV7-3_TRBJ1-2 | 0.78 | 15.71 | 0.00 | 0.26 | 1.65 | 10.66 | 9.46 | 49.26 | 3.69 | 68.38 | 36.09 | 11.54 | 0.06 | 0.59 | 2.62 | no |
| TRBV25-1_TRBJ2-1 | 10.67 | 361.23 | 2.92 | 3.55 | 12.38 | 23.13 | 74.33 | 100.34 | 50477.02 | 93.86 | 68.27 | 337.50 | 0.36 | 0.59 | 6.95 | no |
| TRBV25-1_TRBJ2-5 | 0.31 | 141.35 | 0.00 | 0.13 | 0.14 | 0.40 | 2.03 | 20.34 | 655.70 | 14.63 | 52.18 | 60.15 | 0.35 | 0.59 | 2.50 | no |
| TRBV25-1_TRBJ1-3 | 0.78 | 11.78 | 0.31 | 0.92 | 1.79 | 3.82 | 4.73 | 153.22 | 7.39 | 3397.36 | 2.89 | 2.43 | 0.34 | 0.59 | 7.52 | no |
| TRBV25-1_TRBJ1-5 | 0.78 | 30.10 | 0.31 | 0.13 | 0.28 | 1.41 | 4.05 | 102.60 | 28.17 | 65.60 | 8.46 | 1611.25 | 0.31 | 0.59 | 5.79 | no |
| TRBV25-1_TRBJ2-6 | 0.16 | 22.25 | 0.00 | 0.00 | 0.00 | 0.00 | 0.00 | 0.00 | 79.42 | 12.62 | 6.81 | 0.00 | 0.38 | 0.59 | 2.14 | no |
| TRBV25-1_TRBJ2-4 | 0.00 | 9.16 | 0.00 | 0.00 | 0.00 | 0.00 | 0.00 | 14.01 | 28.63 | 4.29 | 16.71 | 0.00 | 0.11 | 0.59 | 2.80 | no |
| TRBV25-1_TRBJ2-2 | 1.41 | 12037.12 | 1.38 | 0.39 | 1.93 | 4.63 | 10.14 | 20.79 | 49.87 | 3563.13 | 15.68 | 51.04 | 0.53 | 0.66 | -1.70 | no |
| TRBV25-1_TRBJ1-6 | 0.00 | 17.01 | 0.15 | 7.50 | 0.14 | 0.40 | 2.70 | 98.53 | 7.39 | 36.08 | 6.39 | 722.39 | 0.28 | 0.59 | 5.11 | no |
| TRBV25-1_TRBJ2-3 | 1.73 | 318.04 | 0.31 | 0.13 | 1.38 | 3.82 | 10.14 | 42.94 | 357.86 | 35.83 | 3622.48 | 584.47 | 0.27 | 0.59 | 3.84 | no |
| TRBV25-1_TRBJ1-2 | 0.00 | 17.01 | 0.15 | 0.13 | 0.14 | 0.20 | 1.35 | 226.43 | 18.93 | 43.65 | 4.74 | 99.64 | 0.14 | 0.59 | 4.48 | no |
| TRBV25-1_TRBJ1-1 | 5.80 | 133.50 | 2.31 | 1.45 | 6.74 | 13.88 | 39.19 | 28693.79 | 45.25 | 116.32 | 21.86 | 1557.18 | 0.33 | 0.59 | 7.54 | no |
| TRBV25-1_TRBJ1-4 | 67.30 | 865.12 | 27.36 | 22.12 | 79.20 | 140.17 | 478.41 | 419.42 | 344.93 | 270.73 | 148.92 | 242394.87 | 0.36 | 0.59 | 7.67 | no |
| TRBV25-1_TRBJ2-7 | 0.78 | 201.56 | 0.31 | 0.39 | 1.10 | 2.82 | 5.41 | 29.38 | 715.72 | 30.02 | 1735.24 | 559.56 | 0.14 | 0.59 | 3.89 | no |
| TRBV10-1_TRBJ2-2 | 0.00 | 7.85 | 0.00 | 0.00 | 0.00 | 0.00 | 0.00 | 0.45 | 0.00 | 0.25 | 68.48 | 0.00 | 0.41 | 0.59 | 3.14 | no |
| TRBV10-1_TRBJ2-4 | 0.00 | 1.31 | 0.00 | 0.00 | 0.00 | 0.00 | 0.68 | 0.00 | 0.00 | 0.00 | 0.41 | 0.00 | 0.89 | 0.92 | -0.27 | no |
| TRBV10-1_TRBJ2-6 | 0.00 | 1.31 | 0.00 | 0.00 | 0.00 | 0.00 | 0.00 | 0.00 | 0.00 | 0.25 | 0.00 | 0.00 | 0.46 | 0.61 | -2.37 | no |
| TRBV10-1_TRBJ1-6 | 0.00 | 0.00 | 0.00 | 0.00 | 0.00 | 0.00 | 0.00 | 0.00 | 0.00 | 0.25 | 0.00 | 0.00 | 0.36 | 0.59 | Inf | no |
| TRBV10-1_TRBJ1-5 | 0.00 | 0.00 | 0.00 | 0.13 | 0.00 | 0.00 | 0.68 | 0.00 | 0.00 | 33.30 | 0.21 | 0.00 | 0.35 | 0.59 | 8.02 | no |
| TRBV10-1_TRBJ1-3 | 0.00 | 0.00 | 0.00 | 0.00 | 0.00 | 0.00 | 0.00 | 0.00 | 0.00 | 0.25 | 0.21 | 0.00 | 0.18 | 0.59 | Inf | no |
| TRBV10-1_TRBJ2-5 | 0.16 | 7.85 | 0.00 | 0.00 | 0.00 | 0.00 | 1.35 | 4.52 | 26.78 | 15.90 | 1.24 | 0.00 | 0.18 | 0.59 | 2.64 | no |
| TRBV10-1_TRBJ2-1 | 0.00 | 587.66 | 0.00 | 0.00 | 0.14 | 0.40 | 50.68 | 0.45 | 1.85 | 1.01 | 118.60 | 0.30 | 0.52 | 0.65 | -1.77 | no |
| TRBV10-1_TRBJ1-4 | 0.00 | 0.00 | 0.00 | 0.00 | 0.00 | 0.00 | 0.00 | 0.00 | 0.00 | 0.25 | 8.46 | 0.00 | 0.35 | 0.59 | Inf | no |
| TRBV10-1_TRBJ2-7 | 0.00 | 9.16 | 0.00 | 0.00 | 0.00 | 0.00 | 41.22 | 0.00 | 0.00 | 0.50 | 0.83 | 0.00 | 0.46 | 0.61 | 2.22 | no |
| TRBV10-1_TRBJ1-1 | 0.16 | 1.31 | 0.00 | 0.00 | 0.14 | 0.00 | 0.00 | 0.00 | 0.00 | 0.76 | 37.54 | 0.30 | 0.37 | 0.59 | 4.59 | no |
| TRBV10-1_TRBJ1-2 | 0.00 | 0.00 | 0.00 | 0.00 | 0.00 | 0.00 | 0.00 | 0.00 | 0.00 | 0.00 | 0.83 | 0.00 | 0.36 | 0.59 | Inf | no |
| TRBV10-1_TRBJ2-3 | 0.00 | 7.85 | 0.00 | 0.00 | 0.00 | 0.00 | 0.00 | 0.00 | 0.46 | 12.87 | 0.41 | 0.30 | 0.69 | 0.78 | 0.84 | no |
| TRBV16_TRBJ1-3 | 0.00 | 0.00 | 0.00 | 0.00 | 0.00 | 0.00 | 0.00 | 0.00 | 0.46 | 0.00 | 0.00 | 0.00 | 0.36 | 0.59 | Inf | no |
| TRBV16_TRBJ1-5 | 0.00 | 0.00 | 0.00 | 0.00 | 0.00 | 0.00 | 0.00 | 0.45 | 163.46 | 0.25 | 0.00 | 0.00 | 0.36 | 0.59 | Inf | no |
| TRBV16_TRBJ1-4 | 0.00 | 0.00 | 0.00 | 0.00 | 0.00 | 0.00 | 0.00 | 0.45 | 0.00 | 0.00 | 0.00 | 0.00 | 0.36 | 0.59 | Inf | no |
| TRBV16_TRBJ1-1 | 0.00 | 0.00 | 0.00 | 0.00 | 0.00 | 0.00 | 0.00 | 22.15 | 0.92 | 0.00 | 1.24 | 0.00 | 0.31 | 0.59 | Inf | no |
| TRBV20/OR9-2_TRBJ1-1 | 21.80 | 33442.71 | 6.45 | 10.53 | 262.08 | 51.68 | 15528.  84 | 20047.28 | 472.84 | 7571.08 | 31213.  20 | 224.19 | 0.38 | 0.59 | 1.15 | no |
| TRBV20/OR9-2_TRBJ1-4 | 60.24 | 11720.39 | 2.00 | 0.66 | 166.93 | 7.44 | 218.93 | 90.39 | 1823.94 | 2575.33 | 4256.51 | 293.45 | 0.83 | 0.88 | -0.37 | no |
| TRBV20/OR9-2_TRBJ2-7 | 103211.25 | 18680.64 | 37.35 | 33.70 | 832.44 | 518.45 | 30597.  50 | 11760.99 | 3924.48 | 19341.86 | 75030.  31 | 448.68 | 0.89 | 0.92 | 0.19 | no |
| TRBV20/OR9-2_TRBJ2-3 | 931.62 | 990.77 | 16.44 | 8.82 | 43732.  15 | 184.82 | 1235.90 | 6416.07 | 9181.59 | 8535.66 | 3977.24 | 28666.  81 | 0.81 | 0.86 | 0.34 | no |
| TRBV20/OR9-2_TRBJ1-2 | 27.61 | 32865.52 | 5.53 | 6.06 | 138.88 | 42.84 | 73061.  41 | 10319.68 | 138.99 | 7026.84 | 12656.  10 | 68.96 | 0.38 | 0.59 | 1.64 | no |
| TRBV20/OR9-2_TRBJ1-6 | 5.33 | 17701.65 | 1.54 | 1.32 | 19.94 | 14.28 | 61.49 | 5765.70 | 36.48 | 8189.74 | 1836.09 | 218.72 | 0.94 | 0.96 | -0.14 | no |
| TRBV20/OR9-2_TRBJ2-6 | 2.20 | 44.50 | 0.92 | 2.24 | 17.46 | 41.43 | 10.81 | 26.21 | 302.91 | 156.68 | 4713.78 | 2.73 | 0.32 | 0.59 | 5.58 | no |
| TRBV20/OR9-2_TRBJ2-2 | 12.39 | 132.19 | 2.15 | 0.53 | 30.11 | 21.32 | 234.48 | 260.33 | 10206.  23 | 12259.25 | 2250.05 | 21.26 | 0.12 | 0.59 | 6.99 | no |
| TRBV20/OR9-2_TRBJ2-4 | 5.65 | 138.73 | 2.15 | 0.39 | 5.91 | 8.85 | 24249.  07 | 7046.56 | 175.01 | 2651.78 | 254.11 | 10.02 | 0.20 | 0.59 | 7.73 | no |
| TRBV20/OR9-2_TRBJ2-5 | 738.36 | 18962.04 | 26.89 | 36.20 | 79559.  61 | 23643.92 | 9536.51 | 4901.54 | 14513.04 | 18682.32 | 14655.  34 | 251.23 | 0.47 | 0.61 | -0.98 | no |
| TRBV20/OR9-2_TRBJ2-1 | 1137.59 | 1688.36 | 21.98 | 25.28 | 1237.52 | 831.37 | 54343.  83 | 1065.73 | 265572.89 | 19136.98 | 19740.39 | 779.20 | 0.22 | 0.59 | 6.19 | no |
| TRBV20/OR9-2_TRBJ1-5 | 45.65 | 871.67 | 20.29 | 14.61 | 143.41 | 116.04 | 43009.  90 | 8467.08 | 297.37 | 6751.32 | 14117.  43 | 148604.62 | 0.17 | 0.59 | 7.51 | no |
| TRBV20/OR9-2_TRBJ1-3 | 66.67 | 848.11 | 30.12 | 25.28 | 128518.07 | 159.88 | 417.60 | 11338.40 | 368.94 | 1394.77 | 1457.00 | 170.12 | 0.41 | 0.59 | -3.10 | no |
| TRBV5-7_TRBJ1-2 | 0.00 | 1.31 | 0.00 | 0.00 | 24.61 | 1.41 | 0.00 | 0.00 | 0.00 | 0.25 | 1.44 | 0.00 | 0.34 | 0.59 | -4.01 | no |
| TRBV5-7_TRBJ2-3 | 0.00 | 0.00 | 0.00 | 0.00 | 0.00 | 0.00 | 0.00 | 0.90 | 0.00 | 1.26 | 3.09 | 0.00 | 0.14 | 0.59 | Inf | no |
| TRBV5-7_TRBJ2-7 | 0.00 | 0.00 | 0.00 | 0.00 | 0.00 | 0.00 | 0.00 | 0.00 | 0.00 | 0.76 | 3.09 | 0.00 | 0.26 | 0.59 | Inf | no |
| TRBV5-7_TRBJ1-1 | 0.00 | 0.00 | 0.00 | 0.00 | 0.28 | 0.00 | 0.68 | 0.00 | 0.00 | 0.00 | 0.83 | 0.00 | 0.27 | 0.59 | 2.45 | no |
| TRBV5-7_TRBJ1-5 | 0.00 | 0.00 | 0.00 | 0.00 | 0.00 | 0.20 | 0.00 | 1.81 | 0.00 | 0.00 | 0.00 | 0.00 | 0.42 | 0.59 | 3.17 | no |
| TRBV5-7_TRBJ1-3 | 0.00 | 0.00 | 0.00 | 0.00 | 0.00 | 0.00 | 0.00 | 4.07 | 0.00 | 0.00 | 0.00 | 0.00 | 0.36 | 0.59 | Inf | no |
| TRBV5-7_TRBJ2-1 | 0.00 | 0.00 | 0.00 | 0.00 | 0.41 | 0.00 | 0.00 | 0.00 | 0.00 | 0.76 | 0.83 | 0.00 | 0.32 | 0.59 | 1.94 | no |
| TRBV5-7_TRBJ2-5 | 0.00 | 1.31 | 0.00 | 0.00 | 0.28 | 0.00 | 0.00 | 1.81 | 0.00 | 0.00 | 0.21 | 0.00 | 0.85 | 0.89 | 0.35 | no |
| TRBV5-7_TRBJ1-6 | 0.00 | 0.00 | 0.00 | 0.00 | 0.14 | 0.00 | 0.68 | 0.90 | 0.46 | 0.00 | 20.21 | 0.00 | 0.32 | 0.59 | 7.34 | no |
| TRBV5-7_TRBJ2-4 | 0.00 | 0.00 | 0.00 | 0.00 | 0.00 | 0.00 | 0.00 | 0.00 | 0.00 | 0.76 | 0.00 | 0.00 | 0.36 | 0.59 | Inf | no |
| TRBV5-7_TRBJ2-2 | 0.16 | 0.00 | 0.00 | 0.00 | 0.00 | 0.00 | 0.00 | 0.00 | 0.46 | 0.00 | 0.41 | 0.00 | 0.26 | 0.59 | 2.48 | no |
| TRBV29-1_TRBJ1-2 | 4.71 | 516.98 | 0.00 | 0.00 | 2.89 | 4.63 | 307.45 | 639.53 | 7.85 | 622.20 | 220.90 | 0.61 | 0.17 | 0.59 | 1.76 | no |
| TRBV29-1_TRBJ2-3 | 11872.80 | 533.99 | 15.83 | 0.92 | 16.36 | 2841.21 | 59.46 | 54.69 | 55.41 | 1087.46 | 96.94 | 20.96 | 0.28 | 0.59 | -3.47 | no |
| TRBV29-1_TRBJ1-4 | 0.47 | 3.93 | 0.00 | 0.00 | 0.00 | 4.02 | 2.03 | 0.45 | 2.31 | 128.68 | 37.95 | 0.00 | 0.25 | 0.59 | 4.35 | no |
| TRBV29-1_TRBJ2-7 | 4.86 | 976.37 | 1.08 | 0.39 | 42.49 | 6449.64 | 37.84 | 340.33 | 609.52 | 269.72 | 546.99 | 40.71 | 0.41 | 0.59 | -2.02 | no |
| TRBV29-1_TRBJ1-1 | 1.10 | 238.20 | 0.00 | 0.26 | 2.34 | 10.26 | 4.73 | 11.30 | 8830.19 | 1585.77 | 425.71 | 3.04 | 0.27 | 0.59 | 5.43 | no |
| TRBV29-1_TRBJ1-5 | 0.00 | 417.51 | 0.31 | 0.00 | 0.96 | 1597.38 | 6.76 | 2.71 | 7.39 | 5.55 | 66.62 | 2.43 | 0.27 | 0.59 | -4.46 | no |
| TRBV29-1_TRBJ1-3 | 1.10 | 7.85 | 0.00 | 0.26 | 3.71 | 4159.86 | 0.00 | 4.97 | 6.46 | 3.03 | 1.65 | 1.52 | 0.36 | 0.59 | -7.89 | no |
| TRBV29-1_TRBJ2-5 | 10.82 | 166.22 | 32154.22 | 3.29 | 5786.35 | 8180.15 | 746.68 | 46.10 | 769.75 | 306.05 | 35.06 | 27.95 | 0.21 | 0.59 | -4.58 | no |
| TRBV29-1_TRBJ2-1 | 4.08 | 1808.78 | 163.06 | 0.79 | 7274.39 | 58.12 | 193.26 | 385.98 | 611.83 | 313.62 | 460.78 | 892.51 | 0.41 | 0.59 | -1.70 | no |
| TRBV29-1_TRBJ1-6 | 0.00 | 1.31 | 0.00 | 0.00 | 0.00 | 4.83 | 0.00 | 1.81 | 6.93 | 7.82 | 1.65 | 0.30 | 0.23 | 0.59 | 1.59 | no |
| TRBV29-1_TRBJ2-4 | 0.16 | 5.24 | 0.00 | 0.00 | 0.00 | 0.00 | 506.12 | 8.14 | 0.46 | 115.56 | 55.90 | 0.00 | 0.22 | 0.59 | 6.99 | no |
| TRBV29-1_TRBJ2-2 | 4.08 | 22.25 | 1.69 | 0.26 | 2.89 | 2313.11 | 370.97 | 4745.16 | 3133.02 | 2377.77 | 57.34 | 2.73 | 0.16 | 0.59 | 2.19 | no |
| TRBV29-1_TRBJ2-6 | 0.63 | 7.85 | 1.38 | 0.26 | 1.51 | 2943.57 | 1.35 | 2.26 | 8.31 | 2.52 | 1.65 | 0.91 | 0.36 | 0.59 | -7.44 | no |
| TRBV10-3_TRBJ1-3 | 0.00 | 0.00 | 0.00 | 0.00 | 2.75 | 2.41 | 30.41 | 0.90 | 0.00 | 96.89 | 1.03 | 0.00 | 0.25 | 0.59 | 4.65 | no |
| TRBV10-3_TRBJ1-5 | 0.94 | 22.25 | 0.77 | 0.66 | 2511.74 | 2.41 | 17.57 | 10.85 | 51.26 | 65.60 | 5.16 | 5.47 | 0.39 | 0.59 | -4.03 | no |
| TRBV10-3_TRBJ2-1 | 1.73 | 174.07 | 1.84 | 0.92 | 2647.87 | 8.04 | 25.00 | 15.82 | 11.54 | 107.48 | 29.08 | 6.08 | 0.36 | 0.59 | -3.86 | no |
| TRBV10-3_TRBJ2-5 | 0.00 | 9.16 | 0.00 | 0.00 | 21.73 | 4.63 | 4.73 | 9.04 | 22.63 | 43.90 | 2.06 | 0.00 | 0.34 | 0.59 | 1.21 | no |
| TRBV10-3_TRBJ2-6 | 0.16 | 0.00 | 2.92 | 0.00 | 6.19 | 1.21 | 0.00 | 0.00 | 0.46 | 4.04 | 0.21 | 0.30 | 0.46 | 0.61 | -1.06 | no |
| TRBV10-3_TRBJ2-4 | 0.00 | 1.31 | 0.00 | 0.00 | 0.00 | 0.00 | 1.35 | 0.00 | 0.00 | 2.52 | 0.21 | 0.00 | 0.37 | 0.59 | 1.64 | no |
| TRBV10-3_TRBJ2-2 | 0.00 | 15.71 | 0.00 | 0.00 | 0.55 | 2.41 | 2.03 | 0.00 | 1.85 | 5.30 | 0.83 | 0.00 | 0.61 | 0.72 | -0.90 | no |
| TRBV10-3_TRBJ1-6 | 0.00 | 1.31 | 0.00 | 0.00 | 0.96 | 0.00 | 0.00 | 3.62 | 0.00 | 6.56 | 0.21 | 0.00 | 0.29 | 0.59 | 2.19 | no |
| TRBV10-3_TRBJ1-2 | 0.00 | 30.10 | 0.00 | 0.00 | 5.78 | 0.00 | 23.65 | 6.33 | 0.00 | 4.04 | 28.46 | 0.00 | 0.54 | 0.67 | 0.80 | no |
| TRBV10-3_TRBJ2-3 | 1.73 | 31.41 | 0.31 | 0.66 | 2031.45 | 1576.46 | 16.89 | 22.15 | 154.69 | 57.53 | 6.19 | 4.86 | 0.20 | 0.59 | -3.80 | no |
| TRBV10-3_TRBJ1-4 | 1.10 | 23.56 | 0.92 | 0.26 | 2282.67 | 3.82 | 7.43 | 11.30 | 5.54 | 5.30 | 2.89 | 1.82 | 0.36 | 0.59 | -6.08 | no |
| TRBV10-3_TRBJ2-7 | 0.16 | 24.87 | 0.00 | 0.00 | 22.83 | 0.20 | 215.56 | 6.33 | 5.54 | 58.54 | 30.32 | 0.30 | 0.24 | 0.59 | 2.72 | no |
| TRBV10-3_TRBJ1-1 | 1.41 | 252.60 | 0.00 | 0.00 | 7.84 | 0.00 | 10.81 | 65.08 | 0.46 | 13.37 | 11.76 | 0.00 | 0.56 | 0.67 | -1.37 | no |
| TRBV29/OR9-2_TRBJ2-6 | 0.00 | 2.62 | 0.00 | 0.00 | 0.00 | 1.21 | 0.00 | 0.90 | 3.23 | 2.52 | 0.62 | 0.00 | 0.44 | 0.60 | 0.93 | no |
| TRBV29/OR9-2_TRBJ2-4 | 0.00 | 1.31 | 0.00 | 0.00 | 0.00 | 0.00 | 0.00 | 0.00 | 0.00 | 0.00 | 0.21 | 0.00 | 0.44 | 0.60 | -2.67 | no |
| TRBV29/OR9-2_TRBJ2-2 | 0.63 | 1.31 | 0.00 | 0.00 | 0.00 | 0.80 | 1.35 | 2.26 | 0.46 | 6.56 | 0.62 | 0.30 | 0.20 | 0.59 | 2.08 | no |
| TRBV29/OR9-2_TRBJ1-6 | 0.00 | 0.00 | 0.00 | 0.00 | 0.00 | 0.00 | 0.00 | 0.00 | 1.39 | 0.00 | 0.00 | 0.30 | 0.27 | 0.59 | Inf | no |
| TRBV29/OR9-2_TRBJ1-3 | 0.00 | 0.00 | 0.00 | 0.00 | 0.00 | 0.40 | 0.00 | 0.00 | 0.92 | 0.50 | 0.21 | 0.00 | 0.26 | 0.59 | 2.02 | no |
| TRBV29/OR9-2_TRBJ1-5 | 0.00 | 0.00 | 0.00 | 0.00 | 0.00 | 0.20 | 0.00 | 0.00 | 22.16 | 2.27 | 0.21 | 0.00 | 0.31 | 0.59 | 6.94 | no |
| TRBV29/OR9-2_TRBJ2-1 | 0.78 | 7.85 | 5.38 | 0.00 | 13.75 | 6.64 | 2.70 | 5.88 | 1.85 | 3.78 | 4.74 | 4.25 | 0.42 | 0.59 | -0.57 | no |
| TRBV29/OR9-2_TRBJ2-5 | 0.31 | 5.24 | 1.08 | 0.00 | 1.38 | 16.29 | 3.38 | 1.81 | 1.85 | 2.52 | 0.41 | 0.61 | 0.42 | 0.59 | -1.20 | no |
| TRBV29/OR9-2_TRBJ2-7 | 0.16 | 7.85 | 0.00 | 0.00 | 2.34 | 26.34 | 1.35 | 4.52 | 1.39 | 1.51 | 3.09 | 1.52 | 0.40 | 0.59 | -1.46 | no |
| TRBV29/OR9-2_TRBJ1-4 | 0.00 | 0.00 | 0.00 | 0.00 | 0.00 | 0.60 | 0.00 | 0.00 | 1.85 | 0.00 | 0.00 | 0.00 | 0.55 | 0.67 | 1.61 | no |
| TRBV29/OR9-2_TRBJ1-1 | 0.00 | 5.24 | 0.00 | 0.00 | 0.14 | 3.82 | 0.00 | 0.90 | 22.16 | 4.04 | 2.68 | 0.00 | 0.38 | 0.59 | 1.70 | no |
| TRBV29/OR9-2_TRBJ1-2 | 0.16 | 1.31 | 0.00 | 0.00 | 0.41 | 1.01 | 0.68 | 2.26 | 1.39 | 1.77 | 1.44 | 0.00 | 0.08 | 0.59 | 1.38 | no |
| TRBV29/OR9-2_TRBJ2-3 | 1.10 | 0.00 | 0.46 | 0.00 | 2.20 | 4.83 | 0.00 | 0.90 | 2.77 | 3.53 | 2.06 | 2.13 | 0.62 | 0.73 | 0.41 | no |
| TRBV6-3_TRBJ1-1 | 508.24 | 163.60 | 0.00 | 1.05 | 1.51 | 15.08 | 188.53 | 997.93 | 73.42 | 129.69 | 233.28 | 66.53 | 0.35 | 0.59 | 1.29 | no |
| TRBV6-3_TRBJ1-4 | 0.47 | 2.62 | 0.15 | 0.00 | 10.04 | 157.26 | 8.78 | 54.69 | 2.31 | 4.54 | 31.14 | 4.86 | 0.71 | 0.79 | -0.68 | no |
| TRBV6-3_TRBJ2-7 | 1.57 | 209.41 | 0.77 | 2.76 | 243.93 | 83.26 | 54.06 | 1046.75 | 1436.07 | 211.94 | 426.54 | 436.84 | 0.06 | 0.59 | 2.74 | no |
| TRBV6-3_TRBJ2-3 | 2.51 | 36.65 | 121.26 | 0.53 | 30.66 | 3942.66 | 348.00 | 231.86 | 270.59 | 82.51 | 169.75 | 234.21 | 0.51 | 0.64 | -1.63 | no |
| TRBV6-3_TRBJ1-2 | 3.14 | 327.20 | 0.31 | 0.00 | 2.89 | 23.53 | 142.58 | 61.47 | 6.46 | 58.28 | 633.41 | 1.52 | 0.44 | 0.60 | 1.34 | no |
| TRBV6-3_TRBJ1-6 | 0.16 | 5.24 | 2.00 | 0.00 | 0.28 | 9.05 | 5.41 | 12.20 | 2.77 | 87.55 | 12.58 | 3.34 | 0.24 | 0.59 | 2.89 | no |
| TRBV6-3_TRBJ2-4 | 0.00 | 353.38 | 0.00 | 0.00 | 0.00 | 0.00 | 0.00 | 102.14 | 8.77 | 4.29 | 10.11 | 0.00 | 0.56 | 0.67 | -1.50 | no |
| TRBV6-3_TRBJ2-2 | 31.69 | 564.10 | 21616.62 | 9.08 | 35.89 | 5748.39 | 202.04 | 168.58 | 158.84 | 177.88 | 199.24 | 69.87 | 0.26 | 0.59 | -4.84 | no |
| TRBV6-3_TRBJ2-6 | 2.04 | 34.03 | 2448.17 | 0.66 | 145.20 | 21.32 | 5.41 | 18.98 | 45.71 | 264.42 | 16.09 | 5.47 | 0.39 | 0.59 | -2.90 | no |
| TRBV6-3_TRBJ2-1 | 16.78 | 248.67 | 893.36 | 4.61 | 968.42 | 3815.16 | 109.47 | 774.67 | 423.89 | 342.39 | 1778.34 | 33636.  95 | 0.39 | 0.59 | 2.64 | no |
| TRBV6-3_TRBJ2-5 | 4.24 | 107.32 | 155.68 | 0.66 | 21.04 | 4749.50 | 31.08 | 3158.32 | 7857.27 | 38.35 | 431.28 | 116.35 | 0.48 | 0.63 | 1.21 | no |
| TRBV6-3_TRBJ1-5 | 0.63 | 11.78 | 32.58 | 0.66 | 15.54 | 7.44 | 641.26 | 17.63 | 416.97 | 951.46 | 619.59 | 17.92 | 0.04 | 0.59 | 5.28 | up |
| TRBV6-3_TRBJ1-3 | 0.16 | 6.54 | 0.15 | 0.26 | 0.55 | 222.02 | 1.35 | 24.86 | 2.77 | 156.68 | 30.32 | 0.00 | 0.96 | 0.97 | -0.09 | no |
| TRBV15_TRBJ1-3 | 0.00 | 0.00 | 0.00 | 0.00 | 11.69 | 0.20 | 0.00 | 0.00 | 0.00 | 3.28 | 16.50 | 0.00 | 0.70 | 0.79 | 0.73 | no |
| TRBV15_TRBJ1-5 | 0.00 | 1.31 | 0.15 | 0.00 | 46.89 | 5.43 | 0.68 | 0.45 | 6.46 | 980.98 | 62.50 | 5.77 | 0.35 | 0.59 | 4.30 | no |
| TRBV15_TRBJ2-1 | 13.49 | 0.00 | 0.00 | 0.00 | 3.85 | 138.16 | 71.63 | 4.97 | 62.80 | 32.30 | 63.53 | 187.74 | 0.22 | 0.59 | 1.44 | no |
| TRBV15_TRBJ2-5 | 5.96 | 7.85 | 0.15 | 0.00 | 13.34 | 97.54 | 1930.55 | 5.88 | 31.86 | 893.43 | 4517.63 | 79.29 | 0.15 | 0.59 | 5.90 | no |
| TRBV15_TRBJ1-6 | 0.00 | 1.31 | 0.00 | 0.13 | 20.49 | 7.84 | 0.68 | 0.00 | 2.31 | 7.06 | 44.76 | 0.30 | 0.61 | 0.72 | 0.89 | no |
| TRBV15_TRBJ2-4 | 0.00 | 0.00 | 0.00 | 0.00 | 0.00 | 0.00 | 14.87 | 1.36 | 0.92 | 3.53 | 10.11 | 0.00 | 0.09 | 0.59 | Inf | no |
| TRBV15_TRBJ2-2 | 3.61 | 1.31 | 0.15 | 0.00 | 1.51 | 6.64 | 2.70 | 4.52 | 6.93 | 3.78 | 2094.74 | 3.65 | 0.36 | 0.59 | 7.32 | no |
| TRBV15_TRBJ2-6 | 0.00 | 0.00 | 0.00 | 0.13 | 0.69 | 6.23 | 0.00 | 0.00 | 2.77 | 3.78 | 8.04 | 0.00 | 0.47 | 0.61 | 1.05 | no |
| TRBV15_TRBJ1-2 | 435.46 | 2.62 | 0.15 | 0.13 | 70.68 | 4.02 | 3.38 | 3.16 | 6.46 | 2.52 | 2236.03 | 0.61 | 0.48 | 0.62 | 2.13 | no |
| TRBV15_TRBJ2-3 | 18.98 | 24.87 | 1.54 | 1.84 | 12.24 | 16.69 | 38.52 | 569.02 | 27.71 | 22.46 | 8716.40 | 42966.  34 | 0.27 | 0.59 | 9.42 | no |
| TRBV15_TRBJ1-4 | 45661.  22 | 89.00 | 4.15 | 2.90 | 92.26 | 15.69 | 58.11 | 55.59 | 32.32 | 24.73 | 64.35 | 24.30 | 0.36 | 0.59 | -7.47 | no |
| TRBV15_TRBJ2-7 | 64.00 | 14.40 | 0.46 | 0.66 | 22.14 | 18010.97 | 43.92 | 9.04 | 1849.34 | 27.25 | 537.09 | 190.77 | 0.43 | 0.60 | -2.77 | no |
| TRBV15_TRBJ1-1 | 61237.  92 | 503.89 | 15.83 | 12.64 | 79447.  13 | 94.52 | 258.13 | 244.96 | 1167.78 | 152.40 | 19751.  53 | 108.75 | 0.25 | 0.59 | -2.70 | no |
| TRBV24/OR9-2_TRBJ1-1 | 0.00 | 14.40 | 0.00 | 0.00 | 0.69 | 0.40 | 2.70 | 5.42 | 0.46 | 3.78 | 58.37 | 0.91 | 0.37 | 0.59 | 2.21 | no |
| TRBV24/OR9-2_TRBJ1-4 | 0.00 | 688.43 | 0.00 | 0.13 | 0.00 | 0.20 | 2.03 | 121.58 | 0.00 | 0.76 | 1.24 | 0.61 | 0.46 | 0.61 | -2.45 | no |
| TRBV24/OR9-2_TRBJ2-7 | 0.31 | 1.31 | 0.00 | 0.13 | 23.51 | 5.63 | 29.73 | 7.68 | 6.00 | 319.17 | 3.09 | 49.52 | 0.26 | 0.59 | 3.75 | no |
| TRBV24/OR9-2_TRBJ2-3 | 0.31 | 17.01 | 16.44 | 0.13 | 5.50 | 0.80 | 656.13 | 99.43 | 6.93 | 684.77 | 4.13 | 56.81 | 0.13 | 0.59 | 5.23 | no |
| TRBV24/OR9-2_TRBJ1-2 | 0.16 | 9.16 | 0.00 | 0.00 | 1.10 | 0.40 | 0.68 | 1.81 | 0.00 | 1.77 | 69.10 | 0.30 | 0.40 | 0.59 | 2.77 | no |
| TRBV24/OR9-2_TRBJ2-6 | 0.00 | 1.31 | 1.08 | 0.00 | 0.96 | 0.60 | 0.00 | 0.00 | 0.00 | 6.06 | 0.21 | 0.00 | 0.72 | 0.80 | 0.66 | no |
| TRBV24/OR9-2_TRBJ2-4 | 0.16 | 0.00 | 0.00 | 0.13 | 0.00 | 0.00 | 9.46 | 6.78 | 0.46 | 132.21 | 0.83 | 0.00 | 0.30 | 0.59 | 9.02 | no |
| TRBV24/OR9-2_TRBJ2-2 | 0.16 | 331.13 | 12.29 | 0.00 | 0.00 | 0.00 | 8.78 | 0.90 | 1.85 | 207.90 | 45.38 | 2.13 | 0.85 | 0.89 | -0.36 | no |
| TRBV24/OR9-2_TRBJ1-6 | 0.00 | 1.31 | 0.31 | 0.00 | 0.00 | 0.00 | 0.00 | 0.45 | 0.00 | 1.51 | 0.62 | 0.61 | 0.42 | 0.59 | 0.98 | no |
| TRBV24/OR9-2_TRBJ2-5 | 0.47 | 6.54 | 81.45 | 0.00 | 22.83 | 6.44 | 499.36 | 174.91 | 6.46 | 424.39 | 137.99 | 13.97 | 0.08 | 0.59 | 3.42 | no |
| TRBV24/OR9-2_TRBJ2-1 | 11.92 | 141.35 | 12065.  83 | 4.21 | 4867.56 | 506.18 | 935.88 | 68.70 | 412.81 | 692.34 | 82.71 | 2912.64 | 0.35 | 0.59 | -1.79 | no |
| TRBV24/OR9-2_TRBJ1-3 | 0.00 | 0.00 | 0.00 | 0.00 | 0.14 | 0.00 | 0.00 | 2.26 | 0.46 | 0.25 | 38.98 | 0.00 | 0.33 | 0.59 | 8.25 | no |
| TRBV24/OR9-2_TRBJ1-5 | 0.31 | 3.93 | 2.00 | 0.00 | 1.10 | 0.20 | 2.70 | 155.93 | 0.92 | 131.45 | 61.67 | 0.91 | 0.10 | 0.59 | 5.55 | no |
| TRBV3-1_TRBJ2-1 | 0.00 | 0.00 | 0.00 | 0.00 | 0.00 | 0.20 | 0.00 | 0.00 | 0.00 | 0.00 | 0.00 | 0.00 | 0.36 | 0.59 | #NAME? | no |
| TRBV7-2_TRBJ2-6 | 11.29 | 751.26 | 6.92 | 51.74 | 17.46 | 26824.80 | 55.41 | 118.41 | 759.13 | 756.68 | 257.00 | 20.66 | 0.38 | 0.59 | -3.81 | no |
| TRBV7-2_TRBJ2-2 | 1283.94 | 870.36 | 78.99 | 1.97 | 13.48 | 578.58 | 23228.  05 | 1015.11 | 596.13 | 5610.12 | 6416.84 | 22.48 | 0.17 | 0.59 | 3.71 | no |
| TRBV7-2_TRBJ2-4 | 0.47 | 175.38 | 0.61 | 0.26 | 1.38 | 2.41 | 1698.10 | 1450.80 | 60.03 | 529.85 | 1864.15 | 1.22 | 0.05 | 0.59 | 4.96 | up |
| TRBV7-2_TRBJ1-6 | 2.51 | 2745.88 | 2.46 | 1.32 | 11.41 | 55.71 | 52.71 | 571.28 | 435.90 | 885.36 | 2416.50 | 4004.43 | 0.26 | 0.59 | 1.57 | no |
| TRBV7-2_TRBJ2-1 | 95199.68 | 41545.55 | 3067.52 | 62.27 | 365.20 | 2929.70 | 11596.  79 | 83457.74 | 35226.  55 | 31935.93 | 145687.98 | 490.60 | 0.34 | 0.59 | 1.11 | no |
| TRBV7-2_TRBJ2-5 | 2856.98 | 5044.15 | 67.62 | 12.77 | 7927.53 | 1704.77 | 27624.  99 | 57668.71 | 10692.  46 | 26690.14 | 7284.98 | 81.72 | 0.08 | 0.59 | 2.88 | no |
| TRBV7-2_TRBJ1-3 | 1.25 | 666.18 | 0.61 | 2.24 | 11.41 | 14.08 | 16.22 | 2034.29 | 6318.23 | 60.81 | 62.08 | 3.04 | 0.27 | 0.59 | 3.61 | no |
| TRBV7-2_TRBJ1-5 | 2.04 | 591.58 | 16.29 | 16.46 | 9.08 | 767.01 | 1585.93 | 980.31 | 988.16 | 1346.08 | 955.38 | 177.10 | 0.01 | 0.59 | 2.10 | up |
| TRBV7-2_TRBJ1-1 | 53.80 | 35577.37 | 13.06 | 11.06 | 51.29 | 1339.16 | 29217.00 | 35612.90 | 14805.  79 | 24884.11 | 14196.  42 | 307.12 | 0.11 | 0.59 | 1.68 | no |
| TRBV7-2_TRBJ2-7 | 1438.61 | 8433.97 | 11.83 | 16.06 | 1773.50 | 20327.50 | 31271.  88 | 3169.17 | 3018.97 | 68378.37 | 15637.  95 | 224.19 | 0.23 | 0.59 | 1.93 | no |
| TRBV7-2_TRBJ1-4 | 90.98 | 373.01 | 1.23 | 1.45 | 4.26 | 15.08 | 20552.  18 | 170.39 | 90.04 | 1131.61 | 588.86 | 1278.91 | 0.29 | 0.59 | 5.61 | no |
| TRBV7-2_TRBJ2-3 | 419.14 | 43800.63 | 32.89 | 12.51 | 995.51 | 81436.28 | 26018.  11 | 4171.17 | 45618.  41 | 10421.68 | 12574.  63 | 194.42 | 0.78 | 0.84 | -0.36 | no |
| TRBV7-2_TRBJ1-2 | 176.94 | 60659.40 | 9.37 | 40.55 | 1336.79 | 3086.96 | 3373.22 | 86117.54 | 3206.44 | 5998.43 | 8662.36 | 15980.  95 | 0.57 | 0.69 | 0.92 | no |
| TRBV12-3_TRBJ1-3 | 0.00 | 0.00 | 0.00 | 0.00 | 1.51 | 0.20 | 0.00 | 1.36 | 0.00 | 0.00 | 0.62 | 0.00 | 0.90 | 0.93 | 0.20 | no |
| TRBV12-3_TRBJ1-5 | 0.16 | 11.78 | 0.61 | 0.39 | 3.16 | 1.81 | 10.14 | 5.88 | 1.85 | 4.29 | 5.36 | 3200.02 | 0.36 | 0.59 | 7.49 | no |
| TRBV12-3_TRBJ2-5 | 159.22 | 6.54 | 145.23 | 0.00 | 15.81 | 0.40 | 29.73 | 22.15 | 2.77 | 33.81 | 10.52 | 48.60 | 0.39 | 0.59 | -1.15 | no |
| TRBV12-3_TRBJ2-1 | 2.35 | 3.93 | 12.76 | 0.13 | 50.88 | 1.61 | 59.46 | 38.42 | 2.31 | 231.12 | 114.06 | 237.56 | 0.05 | 0.59 | 3.25 | no |
| TRBV12-3_TRBJ2-2 | 0.00 | 1.31 | 2.46 | 0.00 | 3.16 | 0.20 | 1.35 | 3.16 | 0.46 | 27.25 | 2.48 | 0.00 | 0.34 | 0.59 | 2.28 | no |
| TRBV12-3_TRBJ2-6 | 0.00 | 0.00 | 0.61 | 0.00 | 1.24 | 0.40 | 0.00 | 0.45 | 0.00 | 8.07 | 1.03 | 0.00 | 0.40 | 0.59 | 2.08 | no |
| TRBV12-3_TRBJ2-4 | 0.63 | 13.09 | 0.15 | 0.39 | 0.83 | 1.41 | 16.22 | 55.59 | 3.69 | 2879.62 | 3.92 | 1.22 | 0.35 | 0.59 | 7.49 | no |
| TRBV12-3_TRBJ1-6 | 0.00 | 0.00 | 0.00 | 0.00 | 0.14 | 0.60 | 1.35 | 0.45 | 3.69 | 1.26 | 192.44 | 4.86 | 0.33 | 0.59 | 8.11 | no |
| TRBV12-3_TRBJ1-2 | 12.55 | 138.73 | 5.53 | 6.06 | 21813.  28 | 270.08 | 87.17 | 98.98 | 69.73 | 59.29 | 1214.03 | 36.45 | 0.39 | 0.59 | -3.83 | no |
| TRBV12-3_TRBJ2-3 | 7.53 | 89.00 | 15449.93 | 2.50 | 14.85 | 21.32 | 70.28 | 73.22 | 37.86 | 47.94 | 20.42 | 77.77 | 0.37 | 0.59 | -5.57 | no |
| TRBV12-3_TRBJ1-4 | 0.00 | 0.00 | 0.00 | 0.00 | 1.65 | 0.00 | 5.41 | 4.52 | 2.31 | 4.04 | 2.89 | 12.45 | 0.02 | 0.59 | 4.26 | up |
| TRBV12-3_TRBJ2-7 | 4.08 | 30.10 | 1.23 | 0.92 | 22.55 | 5.03 | 736.54 | 13.11 | 13.85 | 203.36 | 425.09 | 8081.16 | 0.28 | 0.59 | 7.21 | no |
| TRBV12-3_TRBJ1-1 | 0.00 | 11.78 | 0.00 | 0.00 | 1.93 | 1.21 | 2.70 | 21.24 | 1.39 | 131.20 | 10.93 | 3.34 | 0.27 | 0.59 | 3.52 | no |
| TRBV27_TRBJ2-2 | 1.88 | 284.01 | 0.46 | 1.05 | 4.13 | 2708.08 | 37.84 | 31.64 | 39.71 | 1619.58 | 103.75 | 4.25 | 0.72 | 0.80 | -0.71 | no |
| TRBV27_TRBJ2-4 | 0.00 | 119.10 | 0.00 | 0.00 | 0.55 | 0.60 | 25.00 | 344.40 | 5.08 | 61.56 | 12.99 | 0.30 | 0.38 | 0.59 | 1.90 | no |
| TRBV27_TRBJ2-6 | 0.16 | 48.43 | 0.15 | 0.00 | 26.54 | 90.90 | 0.00 | 4.07 | 12.93 | 43.15 | 9.08 | 0.00 | 0.36 | 0.59 | -1.26 | no |
| TRBV27_TRBJ1-6 | 15.06 | 264.38 | 4.92 | 4.08 | 37.26 | 31457.25 | 115.55 | 94.01 | 186.55 | 118.59 | 1549.19 | 35.85 | 0.39 | 0.59 | -3.92 | no |
| TRBV27_TRBJ2-5 | 1.73 | 11467.79 | 0.15 | 0.39 | 16.50 | 37.00 | 79.74 | 933.30 | 142.68 | 618.16 | 1434.51 | 10.02 | 0.50 | 0.64 | -1.84 | no |
| TRBV27_TRBJ2-1 | 9.41 | 24622.64 | 3.38 | 2.11 | 37.26 | 125.29 | 3016.44 | 5929.31 | 148.22 | 8185.96 | 1657.48 | 650.09 | 0.85 | 0.89 | -0.34 | no |
| TRBV27_TRBJ1-3 | 0.16 | 5.24 | 0.00 | 0.00 | 7.70 | 138.16 | 2.70 | 13.56 | 47.10 | 8.33 | 18.36 | 0.61 | 0.68 | 0.78 | -0.74 | no |
| TRBV27_TRBJ1-5 | 2.82 | 1164.84 | 0.61 | 0.26 | 38.64 | 110.41 | 4181.39 | 713.65 | 234.11 | 4451.26 | 1706.98 | 6.08 | 0.10 | 0.59 | 3.10 | no |
| TRBV27_TRBJ1-1 | 40.47 | 651.79 | 16.60 | 13.96 | 20726.  88 | 26848.53 | 4748.32 | 284.28 | 60834.  24 | 251.81 | 2599.04 | 110.27 | 0.77 | 0.83 | 0.51 | no |
| TRBV27_TRBJ1-4 | 0.78 | 37.96 | 0.77 | 0.53 | 22.14 | 78.23 | 40.54 | 20.34 | 86.81 | 19.18 | 3625.58 | 5.47 | 0.36 | 0.59 | 4.76 | no |
| TRBV27_TRBJ2-7 | 1.88 | 11516.22 | 0.77 | 0.53 | 30.39 | 106.79 | 488.55 | 135.59 | 129.29 | 1575.93 | 1438.43 | 30.38 | 0.53 | 0.66 | -1.62 | no |
| TRBV27_TRBJ2-3 | 3.29 | 659.64 | 1.23 | 3.42 | 16.64 | 131.92 | 64.87 | 2341.17 | 5579.42 | 2261.96 | 258.23 | 22.78 | 0.13 | 0.59 | 3.69 | no |
| TRBV27_TRBJ1-2 | 34.04 | 520.91 | 12.29 | 10.66 | 17206.83 | 38756.56 | 303.40 | 1880.62 | 384.64 | 191.76 | 649.30 | 80.81 | 0.23 | 0.59 | -4.02 | no |
| TRBV10-2_TRBJ2-3 | 0.00 | 44.50 | 0.00 | 0.00 | 0.00 | 0.20 | 0.00 | 137.40 | 0.46 | 1.01 | 0.21 | 0.00 | 0.54 | 0.66 | 1.64 | no |
| TRBV10-2_TRBJ1-2 | 0.00 | 0.00 | 0.00 | 0.00 | 0.00 | 0.00 | 0.00 | 0.90 | 0.00 | 1.01 | 0.00 | 0.00 | 0.18 | 0.59 | Inf | no |
| TRBV10-2_TRBJ1-1 | 0.00 | 0.00 | 0.00 | 0.00 | 0.00 | 0.00 | 3.38 | 56.95 | 0.00 | 51.47 | 0.00 | 0.00 | 0.16 | 0.59 | Inf | no |
| TRBV10-2_TRBJ2-7 | 0.00 | 130.88 | 0.00 | 0.00 | 0.00 | 0.00 | 0.68 | 7.23 | 0.00 | 1.51 | 0.00 | 0.00 | 0.40 | 0.59 | -3.80 | no |
| TRBV10-2_TRBJ1-4 | 0.00 | 0.00 | 0.00 | 0.00 | 0.00 | 0.00 | 1.35 | 0.45 | 0.00 | 0.00 | 0.00 | 0.00 | 0.24 | 0.59 | Inf | no |
| TRBV10-2_TRBJ2-1 | 0.31 | 197.63 | 0.00 | 0.00 | 0.28 | 0.00 | 0.00 | 0.90 | 0.00 | 36.33 | 0.41 | 0.00 | 0.46 | 0.61 | -2.40 | no |
| TRBV10-2_TRBJ2-5 | 0.00 | 11.78 | 0.00 | 0.00 | 0.00 | 0.00 | 0.00 | 1.36 | 0.46 | 0.76 | 0.00 | 0.00 | 0.47 | 0.61 | -2.19 | no |
| TRBV10-2_TRBJ1-3 | 0.00 | 0.00 | 0.00 | 0.00 | 0.00 | 0.00 | 0.00 | 0.00 | 0.00 | 0.50 | 0.00 | 0.00 | 0.36 | 0.59 | Inf | no |
| TRBV10-2_TRBJ1-5 | 0.00 | 0.00 | 0.00 | 0.00 | 0.00 | 0.00 | 2.03 | 0.00 | 0.00 | 0.50 | 0.00 | 0.00 | 0.26 | 0.59 | Inf | no |
| TRBV10-2_TRBJ1-6 | 0.00 | 0.00 | 0.00 | 0.00 | 0.00 | 0.00 | 160.82 | 0.45 | 0.00 | 0.50 | 0.00 | 0.00 | 0.36 | 0.59 | Inf | no |
| TRBV10-2_TRBJ2-6 | 0.00 | 0.00 | 0.00 | 0.00 | 0.00 | 0.00 | 0.00 | 0.00 | 0.00 | 0.25 | 0.00 | 0.00 | 0.36 | 0.59 | Inf | no |
| TRBV10-2_TRBJ2-2 | 0.00 | 3.93 | 0.00 | 0.00 | 0.00 | 0.00 | 0.00 | 24.41 | 0.00 | 28.01 | 0.00 | 0.00 | 0.21 | 0.59 | 3.74 | no |
| TRBV10-2_TRBJ2-4 | 0.00 | 0.00 | 0.00 | 0.00 | 0.00 | 0.00 | 0.00 | 0.90 | 0.00 | 0.50 | 0.00 | 0.00 | 0.20 | 0.59 | Inf | no |
| TRBV6-7_TRBJ2-1 | 0.16 | 1.31 | 2.46 | 0.00 | 0.28 | 0.00 | 0.68 | 0.00 | 0.46 | 0.00 | 0.41 | 0.00 | 0.34 | 0.59 | -1.44 | no |
| TRBV6-7_TRBJ2-2 | 0.00 | 0.00 | 9.37 | 0.00 | 0.00 | 0.00 | 0.00 | 0.45 | 0.46 | 0.25 | 0.00 | 0.30 | 0.44 | 0.60 | -2.67 | no |
| TRBV6-7_TRBJ2-4 | 0.00 | 0.00 | 0.00 | 0.00 | 0.00 | 0.00 | 0.00 | 0.00 | 0.00 | 0.00 | 0.21 | 0.00 | 0.36 | 0.59 | Inf | no |
| TRBV6-7_TRBJ1-1 | 0.00 | 0.00 | 0.00 | 0.00 | 0.00 | 0.00 | 0.68 | 0.00 | 0.00 | 0.25 | 0.00 | 0.00 | 0.23 | 0.59 | Inf | no |
| TRBV6-7_TRBJ1-4 | 0.00 | 0.00 | 0.00 | 0.00 | 0.14 | 0.00 | 0.00 | 0.00 | 0.00 | 22.71 | 0.00 | 0.00 | 0.37 | 0.59 | 7.37 | no |
| TRBV5-8_TRBJ1-6 | 0.16 | 40.57 | 0.00 | 0.00 | 0.00 | 0.00 | 0.00 | 1.36 | 1.39 | 0.50 | 10.73 | 0.30 | 0.55 | 0.67 | -1.51 | no |
| TRBV5-8_TRBJ2-6 | 0.00 | 71.98 | 0.00 | 0.66 | 0.00 | 5.43 | 0.00 | 0.00 | 0.92 | 15.64 | 0.41 | 0.00 | 0.44 | 0.60 | -2.20 | no |
| TRBV5-8_TRBJ2-4 | 0.00 | 0.00 | 0.00 | 0.00 | 0.00 | 0.00 | 0.00 | 0.45 | 0.00 | 1.01 | 1.24 | 0.00 | 0.10 | 0.59 | Inf | no |
| TRBV5-8_TRBJ2-2 | 508.87 | 1.31 | 0.15 | 0.13 | 0.41 | 1.21 | 7.43 | 5.88 | 8.77 | 4.29 | 27.84 | 0.91 | 0.41 | 0.59 | -3.22 | no |
| TRBV5-8_TRBJ1-5 | 0.00 | 0.00 | 0.00 | 0.00 | 0.00 | 0.20 | 1.35 | 0.00 | 0.00 | 0.00 | 3.51 | 0.30 | 0.21 | 0.59 | 4.68 | no |
| TRBV5-8_TRBJ1-3 | 0.00 | 1.31 | 0.00 | 0.00 | 0.00 | 0.00 | 0.00 | 5.88 | 0.46 | 0.25 | 0.21 | 0.00 | 0.39 | 0.59 | 2.38 | no |
| TRBV5-8_TRBJ2-1 | 17.57 | 30.10 | 0.46 | 0.00 | 0.28 | 2.41 | 92.57 | 78.19 | 11.08 | 71.40 | 287.93 | 0.61 | 0.11 | 0.59 | 3.41 | no |
| TRBV5-8_TRBJ2-5 | 1.25 | 53.66 | 0.00 | 0.00 | 2.89 | 1.21 | 2.70 | 169.49 | 16.16 | 156.43 | 115.30 | 0.30 | 0.10 | 0.59 | 2.96 | no |
| TRBV5-8_TRBJ2-7 | 0.47 | 1.31 | 0.00 | 0.00 | 0.55 | 3.02 | 2.70 | 34.35 | 8.77 | 70.14 | 77.76 | 0.30 | 0.08 | 0.59 | 5.18 | no |
| TRBV5-8_TRBJ1-4 | 0.16 | 0.00 | 0.00 | 0.00 | 0.00 | 0.00 | 4.73 | 0.00 | 5.08 | 9.34 | 26.40 | 0.61 | 0.11 | 0.59 | 8.20 | no |
| TRBV5-8_TRBJ1-1 | 0.16 | 7.85 | 0.00 | 0.13 | 0.41 | 2.01 | 10.81 | 672.52 | 5.54 | 5.55 | 382.40 | 0.61 | 0.19 | 0.59 | 6.67 | no |
| TRBV5-8_TRBJ1-2 | 0.47 | 5.24 | 0.00 | 0.00 | 2.48 | 2.41 | 38.52 | 245.42 | 6.00 | 2.52 | 11.14 | 10.33 | 0.25 | 0.59 | 4.89 | no |
| TRBV5-8_TRBJ2-3 | 0.31 | 5.24 | 0.00 | 0.00 | 1.93 | 10.86 | 3.38 | 164.51 | 51.72 | 91.08 | 4.13 | 0.00 | 0.12 | 0.59 | 4.10 | no |
| TRBV12-5_TRBJ1-5 | 0.63 | 11.78 | 0.15 | 0.13 | 1.10 | 2.21 | 6.76 | 5180.86 | 3.23 | 2.02 | 2.48 | 6.08 | 0.36 | 0.59 | 8.34 | no |
| TRBV12-5_TRBJ1-3 | 0.00 | 0.00 | 0.00 | 0.00 | 0.41 | 0.00 | 0.00 | 33.45 | 0.00 | 0.25 | 0.00 | 0.00 | 0.36 | 0.59 | 6.35 | no |
| TRBV12-5_TRBJ2-5 | 19.29 | 102.09 | 0.31 | 0.00 | 4.54 | 0.60 | 0.68 | 2.71 | 12.47 | 1.77 | 5.36 | 0.30 | 0.34 | 0.59 | -2.45 | no |
| TRBV12-5_TRBJ2-1 | 0.47 | 70.68 | 0.00 | 0.13 | 25.58 | 1.81 | 2.03 | 8.14 | 783.14 | 1.26 | 223.38 | 2.13 | 0.28 | 0.59 | 3.37 | no |
| TRBV12-5_TRBJ2-2 | 9.41 | 120.41 | 6.61 | 4.61 | 17737.45 | 20.51 | 65.55 | 65.53 | 50.79 | 46.93 | 371.47 | 24.61 | 0.37 | 0.59 | -4.84 | no |
| TRBV12-5_TRBJ2-4 | 0.00 | 11.78 | 0.00 | 0.00 | 0.00 | 0.00 | 2.03 | 20.34 | 0.46 | 4.29 | 1.03 | 0.00 | 0.49 | 0.63 | 1.26 | no |
| TRBV12-5_TRBJ2-6 | 0.00 | 1.31 | 0.00 | 0.00 | 0.28 | 0.00 | 0.00 | 0.00 | 1.85 | 0.00 | 0.83 | 0.00 | 0.64 | 0.75 | 0.75 | no |
| TRBV12-5_TRBJ1-6 | 0.00 | 1.31 | 0.00 | 0.00 | 2.06 | 0.00 | 0.00 | 8.59 | 0.00 | 0.00 | 0.21 | 0.30 | 0.54 | 0.66 | 1.43 | no |
| TRBV12-5_TRBJ1-2 | 0.00 | 1.31 | 0.00 | 0.00 | 25.99 | 22.52 | 0.68 | 15.37 | 1.85 | 0.50 | 1.86 | 0.00 | 0.41 | 0.59 | -1.30 | no |
| TRBV12-5_TRBJ2-3 | 0.00 | 20.94 | 13.22 | 0.00 | 4.95 | 0.60 | 0.68 | 306.43 | 4.16 | 0.76 | 2.89 | 6.38 | 0.40 | 0.59 | 3.02 | no |
| TRBV12-5_TRBJ2-7 | 0.31 | 2282.56 | 0.00 | 0.00 | 2.61 | 0.60 | 3.38 | 6.33 | 11.54 | 1.77 | 8.87 | 5.16 | 0.37 | 0.59 | -5.95 | no |
| TRBV12-5_TRBJ1-4 | 0.00 | 2.62 | 0.00 | 0.00 | 0.55 | 0.00 | 0.68 | 11.75 | 0.00 | 0.25 | 1.44 | 0.30 | 0.37 | 0.59 | 2.19 | no |
| TRBV12-5_TRBJ1-1 | 0.00 | 3.93 | 0.00 | 0.00 | 2.48 | 0.20 | 0.00 | 40.68 | 0.46 | 1.77 | 0.62 | 0.30 | 0.40 | 0.59 | 2.73 | no |
| TRBV30_TRBJ1-2 | 0.00 | 344.22 | 0.00 | 0.00 | 1.93 | 1.61 | 0.68 | 21.69 | 0.00 | 0.25 | 13.61 | 0.00 | 0.41 | 0.59 | -3.26 | no |
| TRBV30_TRBJ2-3 | 0.47 | 5.24 | 3.38 | 0.26 | 697.55 | 1.21 | 3.38 | 31.64 | 1.39 | 37.59 | 39.39 | 0.91 | 0.43 | 0.60 | -2.63 | no |
| TRBV30_TRBJ2-7 | 0.00 | 9.16 | 0.00 | 0.00 | 3.30 | 1.41 | 187.18 | 0.90 | 0.00 | 0.76 | 2.89 | 0.00 | 0.38 | 0.59 | 3.79 | no |
| TRBV30_TRBJ1-4 | 0.00 | 1.31 | 0.00 | 0.00 | 0.41 | 0.00 | 0.00 | 0.00 | 0.46 | 20.44 | 0.21 | 0.00 | 0.38 | 0.59 | 3.62 | no |
| TRBV30_TRBJ1-1 | 1.73 | 22.25 | 0.77 | 0.13 | 1444.46 | 1092.20 | 76.36 | 5.88 | 45.71 | 33.81 | 54.04 | 2.73 | 0.21 | 0.59 | -3.55 | no |
| TRBV30_TRBJ1-3 | 0.00 | 0.00 | 0.00 | 0.00 | 0.28 | 1.01 | 0.00 | 0.45 | 0.00 | 0.00 | 0.00 | 0.00 | 0.47 | 0.61 | -1.50 | no |
| TRBV30_TRBJ1-5 | 0.00 | 0.00 | 0.00 | 0.00 | 0.55 | 1.01 | 0.00 | 0.00 | 0.46 | 0.76 | 0.62 | 0.00 | 0.84 | 0.88 | 0.24 | no |
| TRBV30_TRBJ2-1 | 0.78 | 9.16 | 2.46 | 0.39 | 1547.58 | 3.22 | 10.81 | 39.32 | 4.62 | 90.07 | 162.32 | 0.91 | 0.45 | 0.61 | -2.34 | no |
| TRBV30_TRBJ2-5 | 0.00 | 87.69 | 4.92 | 0.00 | 4.81 | 0.20 | 1.35 | 2.71 | 0.00 | 2.02 | 21.04 | 0.00 | 0.46 | 0.61 | -1.85 | no |
| TRBV30_TRBJ1-6 | 0.00 | 0.00 | 0.00 | 0.00 | 0.14 | 0.20 | 0.00 | 0.00 | 0.46 | 0.25 | 0.21 | 0.00 | 0.29 | 0.59 | 1.44 | no |
| TRBV30_TRBJ2-4 | 0.00 | 70.68 | 0.00 | 0.00 | 0.00 | 0.00 | 0.00 | 0.90 | 0.00 | 0.25 | 0.41 | 0.00 | 0.37 | 0.59 | -5.49 | no |
| TRBV30_TRBJ2-6 | 0.00 | 1.31 | 0.46 | 0.00 | 0.55 | 327.00 | 0.00 | 0.45 | 0.00 | 0.25 | 1.03 | 0.00 | 0.36 | 0.59 | -7.57 | no |
| TRBV30_TRBJ2-2 | 1.41 | 18.32 | 3476.16 | 0.79 | 2.61 | 5.43 | 6.76 | 7.23 | 9.70 | 29.52 | 3.71 | 3.95 | 0.37 | 0.59 | -5.85 | no |
| TRBV3-2_TRBJ2-2 | 0.00 | 145.28 | 0.00 | 0.00 | 0.28 | 7.84 | 225.69 | 54.69 | 1.39 | 43.40 | 15.68 | 152.80 | 0.22 | 0.59 | 1.69 | no |
| TRBV3-2_TRBJ2-6 | 0.00 | 1.31 | 0.00 | 0.00 | 0.00 | 6.23 | 0.00 | 0.00 | 0.00 | 1.01 | 0.21 | 0.00 | 0.35 | 0.59 | -2.63 | no |
| TRBV3-2_TRBJ2-4 | 0.00 | 3.93 | 0.00 | 0.00 | 0.14 | 0.20 | 2.03 | 6.33 | 0.46 | 53.99 | 0.00 | 0.30 | 0.31 | 0.59 | 3.89 | no |
| TRBV3-2_TRBJ1-6 | 0.00 | 0.00 | 0.00 | 0.00 | 0.00 | 2.41 | 0.00 | 0.00 | 0.00 | 2.78 | 0.21 | 0.30 | 0.81 | 0.86 | 0.45 | no |
| TRBV3-2_TRBJ2-1 | 0.63 | 11.78 | 0.15 | 0.13 | 0.83 | 2057.71 | 20.27 | 58.30 | 102.51 | 16.40 | 22.89 | 23.69 | 0.41 | 0.59 | -3.09 | no |
| TRBV3-2_TRBJ2-5 | 0.47 | 235.59 | 0.00 | 0.26 | 0.83 | 1095.22 | 6.76 | 16.27 | 60.49 | 185.20 | 50.53 | 3.34 | 0.39 | 0.59 | -2.05 | no |
| TRBV3-2_TRBJ1-5 | 0.00 | 5.24 | 0.00 | 0.00 | 0.00 | 3.22 | 0.00 | 66.44 | 5.54 | 0.50 | 0.62 | 0.00 | 0.37 | 0.59 | 3.11 | no |
| TRBV3-2_TRBJ1-3 | 0.00 | 0.00 | 0.00 | 0.00 | 0.00 | 0.60 | 0.00 | 1.81 | 0.46 | 0.76 | 0.00 | 0.00 | 0.24 | 0.59 | 2.33 | no |
| TRBV3-2_TRBJ1-1 | 0.16 | 187.16 | 0.00 | 0.13 | 0.14 | 5.23 | 1.35 | 1.36 | 181.93 | 111.02 | 26.81 | 0.91 | 0.63 | 0.74 | 0.75 | no |
| TRBV3-2_TRBJ1-4 | 0.00 | 3.93 | 0.00 | 0.00 | 0.00 | 1.61 | 0.68 | 64.18 | 53.10 | 0.00 | 0.41 | 0.00 | 0.19 | 0.59 | 4.42 | no |
| TRBV3-2_TRBJ2-7 | 0.78 | 231.66 | 0.00 | 0.00 | 1.38 | 1222.32 | 316.24 | 244.51 | 23.09 | 88.06 | 31.35 | 16.40 | 0.57 | 0.69 | -1.02 | no |
| TRBV3-2_TRBJ2-3 | 0.63 | 145.28 | 0.46 | 0.00 | 0.55 | 1056.81 | 10.14 | 10.40 | 3.69 | 7.57 | 21.66 | 452.94 | 0.56 | 0.67 | -1.25 | no |
| TRBV3-2_TRBJ1-2 | 0.00 | 7.85 | 0.15 | 0.66 | 1.24 | 1411.76 | 2.03 | 5.42 | 23.09 | 64.59 | 1.65 | 1.52 | 0.39 | 0.59 | -3.85 | no |
| TRBV4-1_TRBJ2-7 | 2637.06 | 180.62 | 0.46 | 0.66 | 55.14 | 4.83 | 32.43 | 20.34 | 914.74 | 226.32 | 190.99 | 17.32 | 0.61 | 0.72 | -1.04 | no |
| TRBV4-1_TRBJ1-4 | 0.47 | 151.82 | 0.00 | 0.00 | 0.14 | 2.61 | 0.68 | 0.90 | 0.46 | 63.08 | 37.13 | 0.61 | 0.76 | 0.83 | -0.59 | no |
| TRBV4-1_TRBJ1-1 | 2.51 | 31.41 | 1.08 | 0.92 | 2.61 | 3987.71 | 343.27 | 366.54 | 12.47 | 200.33 | 47.44 | 6.38 | 0.48 | 0.62 | -2.04 | no |
| TRBV4-1_TRBJ1-2 | 0.63 | 2.62 | 0.00 | 5.27 | 0.41 | 4.22 | 259.48 | 255.36 | 0.92 | 182.17 | 51.15 | 0.30 | 0.06 | 0.59 | 5.83 | no |
| TRBV4-1_TRBJ2-3 | 7.37 | 234.28 | 0.00 | 0.00 | 0.14 | 4.83 | 1.35 | 6.33 | 6.00 | 6.06 | 1.65 | 10.33 | 0.40 | 0.59 | -2.96 | no |
| TRBV4-1_TRBJ2-2 | 1.25 | 128.26 | 0.00 | 0.26 | 0.00 | 0.60 | 1.35 | 1.81 | 1.85 | 0.50 | 19.39 | 0.00 | 0.45 | 0.61 | -2.39 | no |
| TRBV4-1_TRBJ2-4 | 0.00 | 2.62 | 0.00 | 0.26 | 0.00 | 0.00 | 2.70 | 9.04 | 116.36 | 0.76 | 0.41 | 0.00 | 0.32 | 0.59 | 5.49 | no |
| TRBV4-1_TRBJ2-6 | 29.65 | 450.23 | 11.53 | 45707.84 | 36.71 | 64.35 | 203.39 | 173.55 | 186.55 | 196.55 | 78.17 | 81.11 | 0.37 | 0.59 | -5.65 | no |
| TRBV4-1_TRBJ1-6 | 0.00 | 0.00 | 0.00 | 0.00 | 0.00 | 1.61 | 0.00 | 1.81 | 0.00 | 4.29 | 1.44 | 0.00 | 0.23 | 0.59 | 2.23 | no |
| TRBV4-1_TRBJ1-3 | 0.00 | 0.00 | 0.00 | 0.00 | 0.00 | 3.22 | 0.00 | 3.16 | 0.00 | 0.50 | 0.41 | 0.00 | 0.85 | 0.89 | 0.34 | no |
| TRBV4-1_TRBJ1-5 | 0.00 | 87.69 | 0.00 | 0.00 | 0.00 | 2.82 | 4.73 | 2.26 | 36.48 | 32.80 | 52.80 | 0.00 | 0.72 | 0.80 | 0.51 | no |
| TRBV4-1_TRBJ2-5 | 13.65 | 527.45 | 0.31 | 0.39 | 0.96 | 2.01 | 9.46 | 555.91 | 28.17 | 74.68 | 4.33 | 3.95 | 0.86 | 0.90 | 0.31 | no |
| TRBV4-1_TRBJ2-1 | 19.92 | 188.47 | 0.15 | 0.66 | 1.24 | 1.41 | 545.31 | 16.27 | 32.32 | 117.58 | 22.48 | 692.31 | 0.16 | 0.59 | 2.75 | no |
| TRBV13_TRBJ1-2 | 4.39 | 0.00 | 0.00 | 0.00 | 3.99 | 2.01 | 0.00 | 0.45 | 1.39 | 0.25 | 0.21 | 0.61 | 0.20 | 0.59 | -1.84 | no |
| TRBV13_TRBJ2-3 | 3.76 | 0.00 | 0.77 | 0.00 | 0.14 | 1.41 | 1.35 | 1.36 | 68.80 | 7.57 | 0.21 | 0.00 | 0.33 | 0.59 | 3.71 | no |
| TRBV13_TRBJ2-7 | 0.31 | 2.62 | 0.00 | 0.00 | 0.28 | 2.21 | 0.00 | 0.00 | 1.39 | 31.79 | 3.71 | 1.52 | 0.33 | 0.59 | 2.83 | no |
| TRBV13_TRBJ1-4 | 0.00 | 0.00 | 0.00 | 0.00 | 0.00 | 0.00 | 0.00 | 2.26 | 0.00 | 0.00 | 0.00 | 0.00 | 0.36 | 0.59 | Inf | no |
| TRBV13_TRBJ1-1 | 0.00 | 0.00 | 0.00 | 0.00 | 0.69 | 3.82 | 2.03 | 1.36 | 0.92 | 0.25 | 2.06 | 0.00 | 0.64 | 0.74 | 0.55 | no |
| TRBV13_TRBJ1-3 | 0.00 | 0.00 | 0.00 | 0.00 | 0.00 | 0.80 | 0.00 | 0.00 | 0.00 | 0.00 | 0.00 | 0.00 | 0.36 | 0.59 | #NAME? | no |
| TRBV13_TRBJ1-5 | 0.00 | 0.00 | 0.00 | 0.00 | 0.00 | 0.00 | 0.00 | 0.00 | 0.92 | 0.50 | 0.41 | 0.00 | 0.10 | 0.59 | Inf | no |
| TRBV13_TRBJ2-1 | 4.39 | 7.85 | 0.15 | 0.13 | 0.41 | 310.91 | 23.65 | 0.45 | 0.92 | 36.08 | 1.65 | 5.47 | 0.45 | 0.60 | -2.25 | no |
| TRBV13_TRBJ2-5 | 5.65 | 0.00 | 0.00 | 0.00 | 0.00 | 0.40 | 0.00 | 1.36 | 0.92 | 1.77 | 0.00 | 0.00 | 0.75 | 0.82 | -0.58 | no |
| TRBV13_TRBJ2-2 | 0.00 | 0.00 | 0.15 | 0.00 | 0.00 | 0.60 | 0.00 | 0.00 | 0.00 | 0.76 | 0.00 | 0.00 | 1.00 | 1.00 | 0.00 | no |
| TRBV13_TRBJ2-6 | 0.00 | 0.00 | 0.00 | 0.00 | 0.00 | 0.00 | 0.00 | 0.00 | 0.00 | 0.25 | 0.00 | 0.00 | 0.36 | 0.59 | Inf | no |
| TRBV13_TRBJ2-4 | 0.00 | 0.00 | 0.00 | 0.00 | 0.00 | 0.00 | 0.00 | 3.62 | 0.00 | 0.50 | 0.00 | 0.00 | 0.30 | 0.59 | Inf | no |
| TRBV20-1_TRBJ1-6 | 0.00 | 6.54 | 0.00 | 0.00 | 0.00 | 0.20 | 0.00 | 1.81 | 0.00 | 2.52 | 0.62 | 0.00 | 0.81 | 0.86 | -0.45 | no |
| TRBV20-1_TRBJ2-6 | 0.16 | 0.00 | 0.00 | 0.00 | 0.00 | 6.44 | 0.00 | 0.00 | 0.00 | 2.02 | 8.04 | 0.00 | 0.74 | 0.81 | 0.61 | no |
| TRBV20-1_TRBJ2-4 | 0.00 | 0.00 | 0.00 | 0.00 | 0.00 | 0.00 | 4.73 | 0.00 | 0.00 | 2.52 | 0.21 | 0.00 | 0.18 | 0.59 | Inf | no |
| TRBV20-1_TRBJ2-2 | 0.94 | 15.71 | 0.31 | 0.13 | 4.95 | 5.43 | 8.11 | 8.14 | 9.70 | 4915.51 | 3.92 | 2.43 | 0.36 | 0.59 | 7.49 | no |
| TRBV20-1_TRBJ2-5 | 0.16 | 2.62 | 1.69 | 0.00 | 6.88 | 273.10 | 0.00 | 0.45 | 3.23 | 8.83 | 4.95 | 0.30 | 0.37 | 0.59 | -4.00 | no |
| TRBV20-1_TRBJ2-1 | 7.84 | 108.63 | 2.77 | 1.71 | 10.04 | 23466.34 | 42.57 | 43.39 | 57.26 | 57.27 | 25.99 | 15.19 | 0.36 | 0.59 | -6.61 | no |
| TRBV20-1_TRBJ1-5 | 0.00 | 0.00 | 0.00 | 0.00 | 0.14 | 1.01 | 0.68 | 44.74 | 0.46 | 1.51 | 8.46 | 2.13 | 0.24 | 0.59 | 5.66 | no |
| TRBV20-1_TRBJ1-3 | 0.00 | 0.00 | 0.00 | 0.00 | 3.85 | 2.41 | 0.00 | 0.45 | 0.00 | 1.01 | 1.24 | 0.00 | 0.44 | 0.60 | -1.21 | no |
| TRBV20-1_TRBJ1-1 | 0.00 | 9.16 | 0.00 | 0.00 | 0.00 | 0.60 | 0.68 | 4.97 | 0.46 | 8.07 | 13.82 | 0.30 | 0.28 | 0.59 | 1.54 | no |
| TRBV20-1_TRBJ1-4 | 0.63 | 31.41 | 0.15 | 0.13 | 0.41 | 0.80 | 0.00 | 3.62 | 1.85 | 9.34 | 1166.17 | 0.00 | 0.37 | 0.59 | 5.14 | no |
| TRBV20-1_TRBJ2-7 | 23.84 | 45.81 | 0.00 | 0.00 | 0.28 | 131.52 | 10.14 | 5.88 | 0.92 | 17.41 | 96.73 | 0.30 | 0.66 | 0.76 | -0.62 | no |
| TRBV20-1_TRBJ2-3 | 0.31 | 0.00 | 0.00 | 0.00 | 6.05 | 15.69 | 0.68 | 9.94 | 18.01 | 18.42 | 3.51 | 0.91 | 0.27 | 0.59 | 1.22 | no |
| TRBV20-1_TRBJ1-2 | 0.31 | 6.54 | 0.31 | 0.26 | 0.14 | 1.81 | 4.73 | 2.26 | 1.39 | 4.29 | 598.76 | 0.00 | 0.36 | 0.59 | 6.03 | no |
| TRBV7-4_TRBJ2-1 | 2.67 | 2.62 | 49.79 | 0.13 | 31.21 | 147.41 | 14.19 | 64.18 | 19.39 | 59.04 | 146.24 | 1.82 | 0.72 | 0.80 | 0.38 | no |
| TRBV7-4_TRBJ2-5 | 14.27 | 14.40 | 0.61 | 0.00 | 135.16 | 224.23 | 36.49 | 52.88 | 12.47 | 43.90 | 21.04 | 0.00 | 0.38 | 0.59 | -1.22 | no |
| TRBV7-4_TRBJ1-3 | 0.00 | 0.00 | 0.00 | 0.00 | 0.00 | 0.20 | 0.00 | 5.88 | 0.46 | 0.00 | 0.83 | 0.00 | 0.27 | 0.59 | 5.15 | no |
| TRBV7-4_TRBJ1-5 | 0.00 | 6.54 | 0.00 | 0.00 | 0.41 | 69.38 | 9.46 | 2.71 | 0.46 | 9.59 | 6.39 | 3.04 | 0.54 | 0.67 | -1.27 | no |
| TRBV7-4_TRBJ1-6 | 0.00 | 1.31 | 0.00 | 0.00 | 5.91 | 1.41 | 0.00 | 2.26 | 0.92 | 4.54 | 7.63 | 3.04 | 0.29 | 0.59 | 1.09 | no |
| TRBV7-4_TRBJ2-4 | 0.00 | 0.00 | 0.00 | 0.00 | 0.00 | 0.00 | 0.00 | 4.52 | 0.92 | 6.31 | 4.13 | 0.00 | 0.06 | 0.59 | Inf | no |
| TRBV7-4_TRBJ2-2 | 0.63 | 1.31 | 0.46 | 0.00 | 3.16 | 24.33 | 25.00 | 5.42 | 0.00 | 17.91 | 12.79 | 0.30 | 0.38 | 0.59 | 1.04 | no |
| TRBV7-4_TRBJ2-6 | 0.00 | 2.62 | 0.00 | 0.13 | 0.28 | 185.62 | 0.00 | 0.90 | 2.31 | 3.03 | 0.62 | 0.30 | 0.37 | 0.59 | -4.72 | no |
| TRBV7-4_TRBJ2-3 | 0.31 | 3.93 | 1.84 | 0.13 | 5.23 | 580.39 | 62.84 | 6.78 | 12.01 | 22.96 | 3.51 | 1.22 | 0.44 | 0.60 | -2.44 | no |
| TRBV7-4_TRBJ1-2 | 0.00 | 31.41 | 0.15 | 0.00 | 38.23 | 214.38 | 2.03 | 34.35 | 5.08 | 19.43 | 6.39 | 0.91 | 0.34 | 0.59 | -2.06 | no |
| TRBV7-4_TRBJ1-1 | 0.16 | 18.32 | 0.00 | 0.00 | 2.20 | 160.28 | 37.84 | 26.67 | 4.16 | 23.97 | 10.93 | 0.30 | 0.65 | 0.75 | -0.80 | no |
| TRBV7-4_TRBJ1-4 | 0.00 | 1.31 | 0.00 | 0.00 | 0.14 | 0.80 | 1.35 | 0.90 | 1.39 | 2.27 | 0.21 | 12.76 | 0.21 | 0.59 | 3.07 | no |
| TRBV7-4_TRBJ2-7 | 0.47 | 7.85 | 0.00 | 0.00 | 59.54 | 26.75 | 3.38 | 3.16 | 6.46 | 93.35 | 44.96 | 8.20 | 0.56 | 0.67 | 0.75 | no |
| TRBV7-8_TRBJ1-3 | 0.94 | 503.89 | 0.61 | 1.32 | 10.73 | 21.12 | 29.06 | 3829.49 | 3828.43 | 84.02 | 335.17 | 2.43 | 0.17 | 0.59 | 3.91 | no |
| TRBV7-8_TRBJ1-5 | 2.98 | 543.16 | 761.65 | 5.00 | 126.64 | 1067.67 | 2384.63 | 1208.10 | 1818.86 | 2018.74 | 1962.74 | 469.64 | 0.01 | 0.59 | 1.98 | up |
| TRBV7-8_TRBJ2-1 | 37267.  22 | 30469.09 | 14651.  24 | 39.36 | 244.75 | 6645.72 | 11954.  25 | 60318.12 | 29346.  53 | 25089.24 | 94181.  63 | 906.78 | 0.20 | 0.59 | 1.31 | no |
| TRBV7-8_TRBJ2-5 | 10650.  35 | 8255.97 | 342.56 | 11.59 | 5506.26 | 4102.14 | 17133.  68 | 37933.73 | 29186.  30 | 18065.42 | 6673.63 | 101.77 | 0.07 | 0.59 | 1.92 | no |
| TRBV7-8_TRBJ2-2 | 717.34 | 2455.33 | 74.84 | 3.16 | 10.86 | 693.41 | 13677.  35 | 2303.21 | 2083.45 | 4757.82 | 4330.77 | 446.86 | 0.10 | 0.59 | 2.80 | no |
| TRBV7-8_TRBJ2-4 | 0.94 | 244.75 | 0.46 | 0.53 | 1.51 | 1.61 | 879.12 | 2040.16 | 90.50 | 699.91 | 1829.29 | 0.91 | 0.05 | 0.59 | 4.47 | no |
| TRBV7-8_TRBJ2-6 | 5.80 | 870.36 | 26.43 | 1176.45 | 12.79 | 14712.64 | 47.30 | 183.95 | 787.30 | 984.01 | 230.59 | 16.10 | 0.36 | 0.59 | -2.90 | no |
| TRBV7-8_TRBJ1-6 | 2.82 | 3730.11 | 92.21 | 0.66 | 263.59 | 253.79 | 202.04 | 793.20 | 767.44 | 1791.66 | 3866.28 | 2690.58 | 0.27 | 0.59 | 1.22 | no |
| TRBV7-8_TRBJ1-2 | 1348.10 | 31951.97 | 9.68 | 463.68 | 1182.10 | 3688.87 | 2427.88 | 51009.48 | 3110.40 | 5172.61 | 8561.09 | 11653.  61 | 0.45 | 0.61 | 1.08 | no |
| TRBV7-8_TRBJ2-3 | 658.52 | 24999.57 | 223.92 | 7.77 | 1449.96 | 47322.23 | 15699.  79 | 3685.76 | 37889.  05 | 8960.80 | 10627.  57 | 1818.73 | 0.95 | 0.96 | 0.08 | no |
| TRBV7-8_TRBJ2-7 | 1834.85 | 7944.48 | 9.37 | 11.06 | 2873.10 | 11277.37 | 14622.  01 | 4384.04 | 5125.97 | 46547.22 | 15269.  58 | 3597.97 | 0.17 | 0.59 | 1.90 | no |
| TRBV7-8_TRBJ1-4 | 929.74 | 181.92 | 1.23 | 1.05 | 7.15 | 22.72 | 8891.87 | 739.41 | 178.24 | 767.53 | 2953.80 | 3574.58 | 0.10 | 0.59 | 3.90 | no |
| TRBV7-8_TRBJ1-1 | 42.35 | 24926.28 | 4.92 | 6.71 | 45.24 | 1892.40 | 18920.  30 | 22927.19 | 10144.  35 | 18227.15 | 6104.98 | 125.76 | 0.16 | 0.59 | 1.51 | no |
| TRBV6-9_TRBJ1-4 | 0.00 | 0.00 | 0.00 | 0.00 | 3.30 | 3.42 | 0.00 | 0.90 | 0.00 | 0.25 | 0.41 | 0.00 | 0.28 | 0.59 | -2.10 | no |
| TRBV6-9_TRBJ2-7 | 0.00 | 11.78 | 0.00 | 0.00 | 7.01 | 0.00 | 0.68 | 3.62 | 2.31 | 2.27 | 0.41 | 0.61 | 0.52 | 0.65 | -0.93 | no |
| TRBV6-9_TRBJ1-1 | 16.00 | 1.31 | 0.00 | 0.00 | 0.00 | 0.20 | 1.35 | 1.36 | 1.39 | 5.80 | 0.62 | 0.91 | 0.72 | 0.80 | -0.62 | no |
| TRBV6-9_TRBJ1-2 | 0.31 | 14.40 | 0.00 | 0.00 | 0.28 | 0.20 | 10.14 | 1.36 | 0.46 | 76.70 | 0.62 | 0.00 | 0.37 | 0.59 | 2.56 | no |
| TRBV6-9_TRBJ2-3 | 0.00 | 0.00 | 0.46 | 0.00 | 0.83 | 0.20 | 0.00 | 1.81 | 4.62 | 3.03 | 0.00 | 0.00 | 0.16 | 0.59 | 2.67 | no |
| TRBV6-9_TRBJ1-6 | 0.00 | 0.00 | 0.00 | 0.00 | 0.00 | 0.00 | 0.00 | 0.00 | 0.00 | 2.78 | 0.00 | 0.00 | 0.36 | 0.59 | Inf | no |
| TRBV6-9_TRBJ2-2 | 0.31 | 7.85 | 104.66 | 0.00 | 0.41 | 4.02 | 0.68 | 0.90 | 0.46 | 2.52 | 1.03 | 0.00 | 0.33 | 0.59 | -4.39 | no |
| TRBV6-9_TRBJ2-4 | 0.00 | 0.00 | 0.00 | 0.00 | 0.00 | 0.00 | 0.00 | 0.00 | 0.00 | 0.00 | 0.21 | 0.00 | 0.36 | 0.59 | Inf | no |
| TRBV6-9_TRBJ2-6 | 0.31 | 3.93 | 57.17 | 0.00 | 42.35 | 0.00 | 0.00 | 0.00 | 2.31 | 2.02 | 1.44 | 0.30 | 0.18 | 0.59 | -4.09 | no |
| TRBV6-9_TRBJ1-3 | 0.00 | 0.00 | 0.00 | 0.00 | 0.00 | 1.81 | 0.00 | 0.45 | 0.00 | 2.27 | 0.21 | 0.00 | 0.70 | 0.79 | 0.69 | no |
| TRBV6-9_TRBJ1-5 | 0.00 | 1.31 | 0.00 | 0.00 | 0.69 | 0.00 | 1.35 | 0.45 | 39.71 | 1.51 | 1.24 | 0.30 | 0.32 | 0.59 | 4.48 | no |
| TRBV6-9_TRBJ2-1 | 2.67 | 2.62 | 15.68 | 0.00 | 8.94 | 7.44 | 0.68 | 2.71 | 0.92 | 5.05 | 2.68 | 45.26 | 0.67 | 0.77 | 0.62 | no |
| TRBV6-9_TRBJ2-5 | 0.16 | 0.00 | 0.92 | 0.00 | 0.28 | 0.00 | 0.00 | 9.49 | 2.77 | 0.25 | 2.48 | 0.00 | 0.19 | 0.59 | 3.47 | no |
| TRBV23-1_TRBJ1-1 | 0.00 | 0.00 | 0.00 | 0.00 | 0.00 | 0.00 | 0.00 | 0.00 | 0.00 | 0.00 | 0.21 | 0.00 | 0.36 | 0.59 | Inf | no |
| TRBV6-6_TRBJ1-3 | 0.31 | 6.54 | 0.00 | 0.13 | 0.28 | 184.01 | 2.70 | 68.25 | 2.31 | 35.32 | 10.11 | 0.30 | 0.72 | 0.80 | -0.68 | no |
| TRBV6-6_TRBJ1-5 | 0.00 | 5.24 | 19.67 | 0.00 | 8.66 | 3.02 | 124.33 | 17.17 | 882.88 | 78.72 | 207.29 | 2.13 | 0.18 | 0.59 | 5.16 | no |
| TRBV6-6_TRBJ2-5 | 8.00 | 185.85 | 171.51 | 0.26 | 7.56 | 44.24 | 10.14 | 248.13 | 1672.49 | 49.20 | 124.99 | 5.47 | 0.34 | 0.59 | 2.34 | no |
| TRBV6-6_TRBJ2-1 | 20.39 | 188.47 | 3280.98 | 3.42 | 168.30 | 308.70 | 83.11 | 179.88 | 76.65 | 662.82 | 341.77 | 636.72 | 0.56 | 0.67 | -1.00 | no |
| TRBV6-6_TRBJ2-4 | 0.00 | 13.09 | 0.00 | 0.00 | 0.14 | 0.00 | 0.68 | 14.01 | 0.92 | 9.84 | 9.69 | 0.00 | 0.29 | 0.59 | 1.41 | no |
| TRBV6-6_TRBJ2-6 | 2.20 | 61.51 | 1401.90 | 1.32 | 60.78 | 8.85 | 18.24 | 51.07 | 13.39 | 264.42 | 10.11 | 5.77 | 0.44 | 0.60 | -2.08 | no |
| TRBV6-6_TRBJ2-2 | 5.18 | 558.86 | 1385.30 | 0.13 | 3.44 | 213.77 | 41.22 | 25.76 | 42.94 | 168.04 | 55.48 | 3.65 | 0.23 | 0.59 | -2.68 | no |
| TRBV6-6_TRBJ1-6 | 0.16 | 13.09 | 0.00 | 0.00 | 0.55 | 2.21 | 17.57 | 21.69 | 3.23 | 326.49 | 14.85 | 0.61 | 0.30 | 0.59 | 4.59 | no |
| TRBV6-6_TRBJ1-2 | 0.94 | 713.30 | 0.00 | 0.13 | 8.11 | 4.02 | 29.06 | 112.09 | 2.77 | 115.56 | 36.30 | 1.22 | 0.58 | 0.69 | -1.29 | no |
| TRBV6-6_TRBJ2-3 | 1.57 | 20.94 | 47.33 | 0.13 | 3.85 | 31.57 | 11.49 | 247.22 | 229.03 | 290.16 | 107.67 | 4.56 | 0.05 | 0.59 | 3.08 | up |
| TRBV6-6_TRBJ1-4 | 2.04 | 1.31 | 0.00 | 0.00 | 0.83 | 67.97 | 8.11 | 97.62 | 2.31 | 3.28 | 94.05 | 0.91 | 0.35 | 0.59 | 1.52 | no |
| TRBV6-6_TRBJ2-7 | 9.10 | 535.30 | 0.61 | 0.26 | 220.28 | 8.45 | 95.28 | 38.42 | 116.82 | 213.71 | 156.75 | 7.29 | 0.80 | 0.86 | -0.30 | no |
| TRBV6-6_TRBJ1-1 | 437.65 | 668.80 | 0.15 | 0.53 | 1.51 | 6.23 | 650.05 | 96.27 | 102.05 | 345.41 | 90.55 | 131.84 | 0.75 | 0.82 | 0.35 | no |
| TRBV18_TRBJ1-2 | 0.00 | 0.00 | 0.00 | 0.00 | 0.00 | 7.84 | 0.00 | 0.00 | 0.00 | 0.76 | 0.21 | 0.00 | 0.42 | 0.60 | -3.03 | no |
| TRBV18_TRBJ2-3 | 0.00 | 1.31 | 0.00 | 0.00 | 0.00 | 17.90 | 0.00 | 7.23 | 0.00 | 4.29 | 1.65 | 0.00 | 0.76 | 0.83 | -0.54 | no |
| TRBV18_TRBJ1-4 | 0.00 | 0.00 | 0.00 | 0.00 | 0.00 | 22.12 | 0.00 | 0.00 | 0.46 | 3.53 | 1.86 | 0.30 | 0.51 | 0.64 | -1.85 | no |
| TRBV18_TRBJ2-7 | 0.00 | 0.00 | 0.00 | 0.00 | 0.00 | 0.00 | 0.00 | 0.00 | 0.00 | 4.29 | 0.83 | 0.00 | 0.28 | 0.59 | Inf | no |
| TRBV18_TRBJ1-1 | 0.00 | 0.00 | 0.00 | 0.00 | 0.00 | 0.00 | 0.00 | 0.00 | 0.00 | 7.57 | 0.21 | 0.00 | 0.35 | 0.59 | Inf | no |
| TRBV18_TRBJ1-5 | 0.00 | 0.00 | 0.00 | 0.00 | 0.00 | 0.00 | 0.00 | 0.00 | 1.85 | 0.00 | 1.24 | 0.00 | 0.18 | 0.59 | Inf | no |
| TRBV18_TRBJ1-3 | 0.00 | 2.62 | 0.00 | 0.00 | 0.00 | 0.00 | 0.00 | 0.00 | 0.00 | 0.00 | 0.41 | 0.00 | 0.44 | 0.60 | -2.67 | no |
| TRBV18_TRBJ2-5 | 0.00 | 0.00 | 0.00 | 0.00 | 0.00 | 0.20 | 0.00 | 0.00 | 7.39 | 2.52 | 0.00 | 0.00 | 0.24 | 0.59 | 5.62 | no |
| TRBV18_TRBJ2-1 | 0.00 | 13.09 | 0.00 | 0.00 | 0.28 | 0.00 | 10.14 | 0.00 | 0.46 | 3.53 | 25.58 | 0.30 | 0.37 | 0.59 | 1.58 | no |
| TRBV18_TRBJ1-6 | 0.00 | 0.00 | 0.00 | 0.00 | 0.00 | 0.00 | 0.00 | 0.00 | 0.00 | 0.25 | 0.00 | 0.00 | 0.36 | 0.59 | Inf | no |
| TRBV18_TRBJ2-4 | 0.00 | 0.00 | 0.00 | 0.00 | 0.00 | 0.00 | 0.00 | 6.78 | 0.00 | 0.76 | 0.00 | 0.00 | 0.31 | 0.59 | Inf | no |
| TRBV18_TRBJ2-2 | 0.00 | 0.00 | 0.00 | 0.00 | 0.00 | 0.00 | 0.00 | 0.00 | 0.00 | 1.51 | 0.00 | 0.00 | 0.36 | 0.59 | Inf | no |
| TRBV18_TRBJ2-6 | 0.00 | 0.00 | 0.00 | 0.00 | 0.00 | 0.00 | 0.00 | 0.00 | 0.00 | 1.01 | 0.00 | 0.00 | 0.36 | 0.59 | Inf | no |
| TRBV5-1_TRBJ1-5 | 0.00 | 74.60 | 0.00 | 0.00 | 0.00 | 81.45 | 43.25 | 25.31 | 28.63 | 728.17 | 703.13 | 4.56 | 0.18 | 0.59 | 3.30 | no |
| TRBV5-1_TRBJ1-3 | 0.00 | 0.00 | 0.00 | 0.00 | 0.14 | 2.82 | 968.31 | 56.50 | 6.93 | 6.81 | 242.35 | 0.00 | 0.23 | 0.59 | 8.76 | no |
| TRBV5-1_TRBJ2-1 | 2.51 | 2081.01 | 1.69 | 0.66 | 3.30 | 19913.42 | 4379.37 | 3450.29 | 7111.07 | 3677.42 | 466.76 | 204.14 | 0.90 | 0.93 | -0.19 | no |
| TRBV5-1_TRBJ2-5 | 2.67 | 14785.62 | 1.38 | 1.05 | 2.75 | 22234.78 | 172.31 | 3416.84 | 5046.09 | 2026.81 | 89.72 | 5322.23 | 0.43 | 0.60 | -1.20 | no |
| TRBV5-1_TRBJ2-4 | 0.00 | 41.88 | 0.00 | 0.00 | 0.00 | 0.00 | 45.95 | 100.79 | 12.93 | 36.84 | 9.90 | 0.00 | 0.14 | 0.59 | 2.30 | no |
| TRBV5-1_TRBJ2-6 | 0.16 | 2556.11 | 0.00 | 0.13 | 0.00 | 124.69 | 0.00 | 5.42 | 772.52 | 36.08 | 6.60 | 0.30 | 0.51 | 0.65 | -1.71 | no |
| TRBV5-1_TRBJ2-2 | 0.00 | 140.04 | 0.00 | 0.13 | 0.41 | 83.66 | 39.87 | 1976.44 | 705.10 | 67.62 | 1505.26 | 4.56 | 0.11 | 0.59 | 4.26 | no |
| TRBV5-1_TRBJ1-6 | 0.31 | 2948.75 | 0.15 | 0.00 | 0.14 | 44.85 | 2.03 | 27.12 | 9.70 | 517.74 | 27.64 | 1.22 | 0.45 | 0.61 | -2.35 | no |
| TRBV5-1_TRBJ1-2 | 0.63 | 6589.86 | 0.00 | 0.13 | 0.41 | 61.94 | 43.25 | 64.63 | 25.40 | 25.23 | 19.80 | 0.61 | 0.37 | 0.59 | -5.22 | no |
| TRBV5-1_TRBJ2-3 | 0.94 | 2960.53 | 0.00 | 0.92 | 0.41 | 384.51 | 1538.63 | 1537.58 | 232.73 | 2377.02 | 2211.69 | 266.72 | 0.22 | 0.59 | 1.29 | no |
| TRBV5-1_TRBJ1-4 | 0.16 | 44.50 | 0.00 | 0.00 | 0.00 | 53.09 | 1805.54 | 18.08 | 4.62 | 5.05 | 13.61 | 3.65 | 0.37 | 0.59 | 4.24 | no |
| TRBV5-1_TRBJ2-7 | 0.00 | 352.07 | 0.77 | 3.55 | 0.96 | 421.32 | 2333.95 | 86.32 | 4049.61 | 3231.34 | 2576.35 | 179.23 | 0.03 | 0.59 | 4.00 | up |
| TRBV5-1_TRBJ1-1 | 0.16 | 3910.72 | 0.61 | 1.97 | 0.83 | 102.97 | 72.30 | 8386.63 | 1874.74 | 36.58 | 1257.96 | 1.82 | 0.42 | 0.59 | 1.53 | no |
| TRBV6-2_TRBJ2-7 | 1.41 | 160.98 | 0.46 | 2.50 | 236.50 | 77.43 | 52.71 | 1023.70 | 1390.35 | 209.42 | 375.59 | 433.80 | 0.06 | 0.59 | 2.86 | no |
| TRBV6-2_TRBJ1-4 | 1.10 | 1.31 | 0.15 | 0.00 | 9.35 | 157.26 | 6.76 | 50.17 | 4.16 | 5.55 | 23.10 | 4.86 | 0.66 | 0.76 | -0.84 | no |
| TRBV6-2_TRBJ1-1 | 508.40 | 147.90 | 0.15 | 1.05 | 0.83 | 17.50 | 172.99 | 992.06 | 83.58 | 125.90 | 238.43 | 68.05 | 0.34 | 0.59 | 1.31 | no |
| TRBV6-2_TRBJ1-2 | 2.35 | 321.97 | 0.46 | 0.00 | 3.85 | 21.72 | 147.31 | 60.56 | 6.93 | 59.04 | 638.57 | 3.04 | 0.43 | 0.60 | 1.39 | no |
| TRBV6-2_TRBJ2-3 | 2.67 | 47.12 | 123.72 | 0.39 | 29.84 | 3906.67 | 383.14 | 251.74 | 267.82 | 79.98 | 178.62 | 246.97 | 0.52 | 0.65 | -1.55 | no |
| TRBV6-2_TRBJ1-6 | 0.00 | 1.31 | 1.69 | 0.00 | 0.28 | 7.64 | 5.41 | 15.37 | 2.77 | 92.85 | 12.79 | 3.65 | 0.22 | 0.59 | 3.60 | no |
| TRBV6-2_TRBJ2-2 | 28.39 | 537.92 | 20984.68 | 10.40 | 34.10 | 5696.90 | 185.15 | 180.33 | 174.54 | 171.32 | 181.51 | 67.44 | 0.25 | 0.59 | -4.83 | no |
| TRBV6-2_TRBJ2-4 | 0.00 | 358.61 | 0.00 | 0.13 | 0.14 | 0.00 | 2.03 | 112.09 | 8.77 | 3.03 | 10.52 | 0.00 | 0.57 | 0.69 | -1.40 | no |
| TRBV6-2_TRBJ2-6 | 1.57 | 26.18 | 2414.36 | 0.92 | 150.98 | 23.53 | 10.81 | 23.05 | 43.41 | 250.04 | 19.59 | 4.86 | 0.39 | 0.59 | -2.90 | no |
| TRBV6-2_TRBJ1-5 | 0.47 | 13.09 | 30.74 | 1.97 | 15.54 | 9.85 | 653.43 | 17.17 | 407.73 | 913.11 | 641.04 | 11.54 | 0.04 | 0.59 | 5.21 | up |
| TRBV6-2_TRBJ1-3 | 0.00 | 2.62 | 0.00 | 0.26 | 0.28 | 220.21 | 2.03 | 23.50 | 2.77 | 160.72 | 25.37 | 0.30 | 0.97 | 0.98 | -0.06 | no |
| TRBV6-2_TRBJ2-1 | 14.90 | 227.73 | 815.75 | 4.61 | 920.44 | 3789.62 | 83.79 | 775.12 | 416.04 | 340.87 | 1568.99 | 33623.89 | 0.39 | 0.59 | 2.67 | no |
| TRBV6-2_TRBJ2-5 | 2.98 | 130.88 | 144.77 | 0.39 | 21.86 | 4722.15 | 34.46 | 3178.66 | 6363.02 | 35.07 | 433.96 | 121.51 | 0.53 | 0.66 | 1.02 | no |
| TRBV5-5_TRBJ2-7 | 0.47 | 2.62 | 0.00 | 0.00 | 21.04 | 1.21 | 6.08 | 32.99 | 7.85 | 48.19 | 28.67 | 0.00 | 0.09 | 0.59 | 2.29 | no |
| TRBV5-5_TRBJ1-4 | 0.00 | 0.00 | 0.00 | 0.13 | 0.00 | 0.20 | 2.70 | 0.00 | 10.16 | 13.37 | 23.31 | 19.75 | 0.03 | 0.59 | 7.70 | up |
| TRBV5-5_TRBJ1-1 | 0.16 | 14.40 | 0.00 | 0.13 | 1.51 | 8.04 | 17.57 | 440.66 | 7.39 | 5.55 | 85.18 | 1.22 | 0.26 | 0.59 | 4.52 | no |
| TRBV5-5_TRBJ1-2 | 0.31 | 20.94 | 0.00 | 0.00 | 219.87 | 8.65 | 293.94 | 77.74 | 20.78 | 2.78 | 13.41 | 4.56 | 0.65 | 0.76 | 0.73 | no |
| TRBV5-5_TRBJ2-3 | 0.16 | 2.62 | 0.00 | 0.00 | 12.79 | 1.41 | 22.97 | 219.20 | 36.48 | 119.85 | 7.01 | 0.61 | 0.12 | 0.59 | 4.58 | no |
| TRBV5-5_TRBJ1-6 | 0.31 | 56.28 | 0.00 | 0.00 | 1.51 | 0.20 | 0.00 | 3.62 | 0.92 | 2.02 | 122.31 | 0.91 | 0.61 | 0.72 | 1.15 | no |
| TRBV5-5_TRBJ2-4 | 0.00 | 1.31 | 0.00 | 0.00 | 0.00 | 0.00 | 1.35 | 2.26 | 0.00 | 0.50 | 2.27 | 0.00 | 0.12 | 0.59 | 2.29 | no |
| TRBV5-5_TRBJ2-6 | 0.00 | 86.38 | 0.00 | 1.32 | 0.00 | 1.21 | 0.00 | 0.45 | 0.46 | 52.48 | 1.24 | 0.30 | 0.74 | 0.82 | -0.69 | no |
| TRBV5-5_TRBJ2-2 | 177.57 | 13.09 | 0.00 | 0.00 | 0.14 | 3.42 | 8.11 | 4.97 | 4.16 | 4.79 | 8.87 | 0.30 | 0.39 | 0.59 | -2.64 | no |
| TRBV5-5_TRBJ1-5 | 0.16 | 1.31 | 0.00 | 0.00 | 0.14 | 6.23 | 5.41 | 0.45 | 4.16 | 5.30 | 7.01 | 0.00 | 0.15 | 0.59 | 1.51 | no |
| TRBV5-5_TRBJ1-3 | 0.16 | 0.00 | 0.00 | 0.00 | 0.28 | 0.00 | 0.00 | 18.98 | 0.00 | 0.00 | 0.41 | 0.00 | 0.36 | 0.59 | 5.49 | no |
| TRBV5-5_TRBJ2-5 | 0.31 | 90.31 | 0.15 | 0.13 | 33.69 | 2.41 | 18.92 | 138.75 | 12.01 | 108.75 | 296.39 | 0.30 | 0.18 | 0.59 | 2.18 | no |
| TRBV5-5_TRBJ2-1 | 2.35 | 32.72 | 1.08 | 0.13 | 0.83 | 6.84 | 670.99 | 130.62 | 10.16 | 87.05 | 75.49 | 0.30 | 0.19 | 0.59 | 4.47 | no |
| TRBV5-4_TRBJ1-6 | 0.00 | 31.41 | 0.00 | 0.00 | 0.00 | 0.00 | 0.00 | 1.81 | 0.00 | 0.25 | 0.62 | 0.00 | 0.40 | 0.59 | -3.55 | no |
| TRBV5-4_TRBJ2-6 | 0.00 | 47.12 | 0.00 | 0.00 | 0.00 | 0.60 | 0.00 | 0.00 | 0.00 | 0.50 | 0.00 | 0.00 | 0.36 | 0.59 | -6.56 | no |
| TRBV5-4_TRBJ2-4 | 0.00 | 0.00 | 0.00 | 0.00 | 0.00 | 0.00 | 0.00 | 2.26 | 0.00 | 0.00 | 0.41 | 0.00 | 0.28 | 0.59 | Inf | no |
| TRBV5-4_TRBJ2-2 | 164.71 | 2.62 | 0.00 | 0.13 | 0.00 | 0.20 | 2.70 | 1.81 | 7.85 | 0.76 | 0.21 | 0.00 | 0.39 | 0.59 | -3.65 | no |
| TRBV5-4_TRBJ2-5 | 0.00 | 30.10 | 0.00 | 0.00 | 0.28 | 0.00 | 0.68 | 64.18 | 6.93 | 21.70 | 10.93 | 0.30 | 0.30 | 0.59 | 1.79 | no |
| TRBV5-4_TRBJ2-1 | 0.47 | 2.62 | 0.00 | 0.00 | 0.00 | 0.00 | 0.00 | 13.56 | 1.39 | 19.68 | 11.96 | 0.00 | 0.09 | 0.59 | 3.92 | no |
| TRBV5-4_TRBJ1-3 | 0.00 | 0.00 | 0.00 | 0.00 | 0.00 | 0.00 | 0.00 | 4.07 | 0.46 | 0.00 | 0.00 | 0.00 | 0.31 | 0.59 | Inf | no |
| TRBV5-4_TRBJ1-5 | 0.00 | 0.00 | 0.00 | 0.00 | 0.00 | 0.80 | 0.00 | 1.36 | 0.00 | 0.76 | 0.21 | 0.00 | 0.37 | 0.59 | 1.53 | no |
| TRBV5-4_TRBJ1-1 | 0.16 | 0.00 | 0.00 | 0.00 | 0.00 | 0.20 | 1.35 | 287.00 | 0.92 | 0.76 | 2.27 | 0.00 | 0.35 | 0.59 | 9.67 | no |
| TRBV5-4_TRBJ1-4 | 0.31 | 1.31 | 0.00 | 0.00 | 0.00 | 0.00 | 0.00 | 2.71 | 6.00 | 5.55 | 19.39 | 0.00 | 0.13 | 0.59 | 4.37 | no |
| TRBV5-4_TRBJ2-7 | 0.63 | 0.00 | 0.00 | 0.00 | 0.83 | 0.00 | 0.00 | 6.78 | 3.23 | 25.74 | 1.03 | 0.00 | 0.21 | 0.59 | 4.66 | no |
| TRBV5-4_TRBJ2-3 | 0.31 | 0.00 | 0.00 | 0.00 | 0.00 | 0.40 | 0.68 | 51.98 | 30.94 | 37.34 | 0.00 | 0.00 | 0.08 | 0.59 | 7.40 | no |
| TRBV5-4_TRBJ1-2 | 0.00 | 1.31 | 0.00 | 0.00 | 0.14 | 0.00 | 0.00 | 87.23 | 7.39 | 0.00 | 1.44 | 0.30 | 0.32 | 0.59 | 6.06 | no |
| TRBV4-2_TRBJ1-6 | 0.00 | 260.45 | 0.00 | 0.00 | 0.28 | 0.00 | 0.68 | 17.17 | 7.39 | 6.06 | 45.17 | 3.34 | 0.52 | 0.65 | -1.71 | no |
| TRBV4-2_TRBJ2-2 | 2304.98 | 54.97 | 1.08 | 0.00 | 2.75 | 5.03 | 14.87 | 21.24 | 56.33 | 368.88 | 38.36 | 4.25 | 0.46 | 0.61 | -2.23 | no |
| TRBV4-2_TRBJ2-4 | 0.00 | 7.85 | 0.00 | 0.00 | 0.00 | 0.00 | 6.76 | 28.93 | 1.85 | 6.56 | 5.16 | 0.00 | 0.18 | 0.59 | 2.65 | no |
| TRBV4-2_TRBJ2-6 | 0.31 | 439.76 | 0.15 | 133.36 | 0.28 | 0.40 | 3.38 | 4.07 | 35.56 | 47.94 | 2.27 | 0.30 | 0.32 | 0.59 | -2.62 | no |
| TRBV4-2_TRBJ2-5 | 1.41 | 399.19 | 0.15 | 0.13 | 10.04 | 2.21 | 14.87 | 706.42 | 184.70 | 287.38 | 200.48 | 0.30 | 0.22 | 0.59 | 1.75 | no |
| TRBV4-2_TRBJ2-1 | 8.94 | 187.16 | 0.61 | 0.26 | 10.45 | 10.66 | 577.07 | 250.84 | 158.84 | 215.22 | 550.29 | 5.16 | 0.04 | 0.59 | 3.01 | up |
| TRBV4-2_TRBJ1-3 | 0.00 | 0.00 | 0.00 | 0.00 | 0.14 | 0.40 | 0.00 | 47.00 | 8.31 | 1.26 | 1.65 | 0.00 | 0.26 | 0.59 | 6.75 | no |
| TRBV4-2_TRBJ1-5 | 0.00 | 2.62 | 0.15 | 0.00 | 0.28 | 3.42 | 12.16 | 27.57 | 266.90 | 9.84 | 12.79 | 0.00 | 0.26 | 0.59 | 5.67 | no |
| TRBV4-2_TRBJ1-1 | 0.94 | 34.03 | 0.00 | 0.79 | 0.28 | 12.87 | 25.68 | 2832.00 | 269.20 | 18.17 | 298.66 | 3.34 | 0.27 | 0.59 | 6.14 | no |
| TRBV4-2_TRBJ1-4 | 0.94 | 0.00 | 0.00 | 0.13 | 0.41 | 0.40 | 2.03 | 16.27 | 57.26 | 36.58 | 200.27 | 25.52 | 0.12 | 0.59 | 7.48 | no |
| TRBV4-2_TRBJ2-7 | 8.31 | 30.10 | 0.00 | 0.13 | 27.64 | 0.60 | 27.03 | 199.77 | 198.56 | 151.39 | 79.20 | 1.82 | 0.04 | 0.59 | 3.30 | up |
| TRBV4-2_TRBJ2-3 | 11.45 | 18.32 | 0.15 | 0.53 | 7.43 | 4.22 | 27.70 | 760.65 | 295.06 | 575.77 | 14.44 | 1.82 | 0.10 | 0.59 | 5.31 | no |
| TRBV4-2_TRBJ1-2 | 0.94 | 24.87 | 0.00 | 0.79 | 21.86 | 5.43 | 279.07 | 889.01 | 67.88 | 11.35 | 15.26 | 27.95 | 0.20 | 0.59 | 4.58 | no |
| TRBV28_TRBJ1-6 | 11.92 | 163.60 | 170.90 | 2.76 | 55.83 | 21941.56 | 68.25 | 56.95 | 70.19 | 6516.92 | 1468.96 | 30.68 | 0.56 | 0.67 | -1.44 | no |
| TRBV28_TRBJ2-6 | 9.10 | 113.87 | 70.23 | 2.37 | 42.76 | 20853.18 | 54.73 | 46.10 | 48.02 | 1691.99 | 1590.44 | 16.71 | 0.44 | 0.60 | -2.61 | no |
| TRBV28_TRBJ2-4 | 0.31 | 14.40 | 0.00 | 0.00 | 0.28 | 0.20 | 98.66 | 50.17 | 1.39 | 313.37 | 166.86 | 0.30 | 0.09 | 0.59 | 5.38 | no |
| TRBV28_TRBJ2-2 | 216.00 | 7352.89 | 203.17 | 0.39 | 34.93 | 87.68 | 55.41 | 25.31 | 12.93 | 4279.44 | 612.79 | 19.44 | 0.74 | 0.81 | -0.66 | no |
| TRBV28_TRBJ2-5 | 850.99 | 98.16 | 15.06 | 0.92 | 412.92 | 808.84 | 277.72 | 110.73 | 19.39 | 3422.84 | 2353.59 | 143.99 | 0.31 | 0.59 | 1.53 | no |
| TRBV28_TRBJ2-1 | 1454.92 | 303.64 | 226.99 | 5.92 | 527.04 | 18376.58 | 589.23 | 866.87 | 118.67 | 38504.58 | 3691.37 | 1006.42 | 0.58 | 0.69 | 1.10 | no |
| TRBV28_TRBJ1-5 | 257.89 | 10517.59 | 343287.04 | 78.07 | 98938.97 | 47624.69 | 7807.33 | 1379.84 | 1253.67 | 12219.14 | 29814.16 | 626.39 | 0.23 | 0.59 | -3.24 | no |
| TRBV28_TRBJ1-3 | 0.00 | 1.31 | 0.15 | 0.13 | 19.94 | 78.23 | 2.70 | 1.36 | 9.70 | 67.11 | 46.20 | 0.61 | 0.79 | 0.85 | 0.36 | no |
| TRBV28_TRBJ1-1 | 268.55 | 134.81 | 2.31 | 0.92 | 90.48 | 201.71 | 125.01 | 31.64 | 98.35 | 9786.11 | 2902.23 | 14.28 | 0.26 | 0.59 | 4.21 | no |
| TRBV28_TRBJ2-7 | 151399.41 | 2700.07 | 74.54 | 54.11 | 36304.17 | 70395.21 | 13425.98 | 1120.87 | 975.69 | 47287.50 | 92978.54 | 34693.19 | 0.69 | 0.78 | -0.45 | no |
| TRBV28_TRBJ1-4 | 178021.13 | 1633.39 | 41.49 | 29.62 | 223.58 | 318.15 | 881.15 | 676.59 | 478.84 | 492.51 | 1774.63 | 256.09 | 0.37 | 0.59 | -5.31 | no |
| TRBV28_TRBJ2-3 | 386.04 | 214.64 | 105.12 | 1.71 | 78.38 | 13508.43 | 189.20 | 2978.44 | 67.88 | 3279.53 | 9486.15 | 443.82 | 0.90 | 0.93 | 0.20 | no |
| TRBV28_TRBJ1-2 | 332.87 | 246.06 | 5.23 | 5.13 | 207.08 | 29250.93 | 7293.10 | 84.07 | 5237.72 | 7920.27 | 1255.48 | 38.88 | 0.80 | 0.85 | -0.46 | no |
| TRBV6-1_TRBJ1-4 | 17.26 | 20.94 | 0.31 | 0.39 | 482.22 | 34.19 | 9.46 | 837.04 | 4.16 | 9.59 | 61.67 | 2.73 | 0.71 | 0.79 | 0.74 | no |
| TRBV6-1_TRBJ2-7 | 9.57 | 443.69 | 4.76 | 3.69 | 13757.60 | 28.56 | 92.57 | 4435.12 | 1409.28 | 1139.69 | 123.14 | 62.58 | 0.64 | 0.75 | -0.97 | no |
| TRBV6-1_TRBJ1-1 | 292.08 | 82.46 | 1.54 | 1.18 | 79.34 | 10.66 | 172.31 | 4495.23 | 18.47 | 971.14 | 1373.46 | 99.03 | 0.17 | 0.59 | 3.93 | no |
| TRBV6-1_TRBJ1-2 | 259.93 | 1222.43 | 1.84 | 1.71 | 14.71 | 10.46 | 29896.10 | 1112.28 | 26.32 | 2278.87 | 45.38 | 12.76 | 0.33 | 0.59 | 4.46 | no |
| TRBV6-1_TRBJ2-3 | 40.00 | 2862.37 | 4.30 | 0.39 | 975.16 | 340.67 | 10.81 | 107.57 | 598.44 | 143.06 | 37.95 | 3.95 | 0.29 | 0.59 | -2.23 | no |
| TRBV6-1_TRBJ1-6 | 1.25 | 10.47 | 0.00 | 0.26 | 1.24 | 1.81 | 31.08 | 18.98 | 3.69 | 1633.96 | 14.64 | 2.43 | 0.34 | 0.59 | 6.83 | no |
| TRBV6-1_TRBJ2-4 | 0.00 | 5.24 | 0.00 | 0.00 | 0.00 | 0.20 | 0.68 | 30.28 | 0.46 | 11.35 | 13.82 | 0.00 | 0.14 | 0.59 | 3.38 | no |
| TRBV6-1_TRBJ2-2 | 1.25 | 433.22 | 11.37 | 0.26 | 68.06 | 115.84 | 20.95 | 17.17 | 12.47 | 97.90 | 57.75 | 1.82 | 0.36 | 0.59 | -1.60 | no |
| TRBV6-1_TRBJ2-6 | 56.94 | 722.46 | 962.21 | 18.17 | 107467.38 | 152.64 | 346.65 | 338.97 | 314.46 | 332.55 | 1604.47 | 155.84 | 0.37 | 0.59 | -5.14 | no |
| TRBV6-1_TRBJ1-5 | 1.41 | 28.79 | 1.08 | 0.26 | 458.57 | 3.82 | 87.17 | 17.17 | 1089.75 | 1197.21 | 339.91 | 5.47 | 0.16 | 0.59 | 2.47 | no |
| TRBV6-1_TRBJ1-3 | 0.63 | 9.16 | 0.15 | 0.13 | 1.38 | 7.64 | 8.78 | 11.75 | 6.93 | 634.81 | 1686.15 | 1.52 | 0.22 | 0.59 | 6.94 | no |
| TRBV6-1_TRBJ2-1 | 33.26 | 184.54 | 715.70 | 1.71 | 262.63 | 307.29 | 39.19 | 960.42 | 53.56 | 1938.50 | 499.97 | 9.72 | 0.35 | 0.59 | 1.22 | no |
| TRBV6-1_TRBJ2-5 | 1.41 | 108.63 | 11.07 | 10.27 | 167.34 | 20.11 | 5.41 | 138.75 | 79.88 | 48.70 | 53.42 | 1.22 | 0.97 | 0.98 | 0.04 | no |
| TRBV4-3_TRBJ2-1 | 6.27 | 81.15 | 0.15 | 0.00 | 34.79 | 2.21 | 168.26 | 8.14 | 565.65 | 33.81 | 771.19 | 248.80 | 0.08 | 0.59 | 3.85 | no |
| TRBV4-3_TRBJ2-5 | 3.76 | 159.67 | 0.15 | 0.26 | 37.26 | 2.82 | 7.43 | 140.56 | 650.62 | 33.30 | 90.55 | 0.30 | 0.30 | 0.59 | 2.18 | no |
| TRBV4-3_TRBJ1-3 | 0.00 | 0.00 | 0.00 | 0.00 | 0.00 | 0.60 | 0.00 | 2.26 | 0.46 | 0.00 | 0.83 | 0.00 | 0.24 | 0.59 | 2.56 | no |
| TRBV4-3_TRBJ1-5 | 0.00 | 31.41 | 0.00 | 0.00 | 0.00 | 1.21 | 1.35 | 0.45 | 13.39 | 11.61 | 20.42 | 0.00 | 0.71 | 0.79 | 0.53 | no |
| TRBV4-3_TRBJ1-6 | 0.00 | 1.31 | 0.00 | 0.00 | 0.28 | 0.40 | 1.35 | 0.45 | 0.92 | 1.26 | 232.86 | 0.00 | 0.36 | 0.59 | 6.90 | no |
| TRBV4-3_TRBJ2-2 | 8.63 | 53.66 | 0.00 | 0.00 | 0.00 | 0.40 | 0.68 | 0.90 | 7.39 | 2.27 | 9.49 | 0.00 | 0.47 | 0.61 | -1.60 | no |
| TRBV4-3_TRBJ2-4 | 0.00 | 0.00 | 0.00 | 0.00 | 0.00 | 0.00 | 0.00 | 3.62 | 57.26 | 0.00 | 2.27 | 0.00 | 0.31 | 0.59 | Inf | no |
| TRBV4-3_TRBJ2-6 | 10.35 | 157.06 | 4.30 | 13911.34 | 12.51 | 17.70 | 68.25 | 64.63 | 75.73 | 64.59 | 26.81 | 30.99 | 0.37 | 0.59 | -5.41 | no |
| TRBV4-3_TRBJ2-3 | 1.41 | 77.22 | 0.15 | 0.00 | 4.26 | 0.40 | 2.03 | 10.85 | 110.36 | 3.03 | 113.03 | 3.65 | 0.33 | 0.59 | 1.54 | no |
| TRBV4-3_TRBJ1-2 | 0.00 | 3.93 | 0.00 | 5.27 | 0.83 | 1.41 | 80.41 | 67.34 | 5.08 | 51.72 | 329.80 | 0.30 | 0.14 | 0.59 | 5.55 | no |
| TRBV4-3_TRBJ1-1 | 0.47 | 6.54 | 0.31 | 0.13 | 0.83 | 750.73 | 123.66 | 122.03 | 9.24 | 52.48 | 514.82 | 0.30 | 0.94 | 0.96 | 0.12 | no |
| TRBV4-3_TRBJ1-4 | 0.00 | 49.73 | 0.00 | 0.00 | 0.00 | 0.60 | 0.00 | 0.45 | 0.00 | 19.68 | 37.13 | 0.00 | 0.91 | 0.94 | 0.19 | no |
| TRBV4-3_TRBJ2-7 | 696.79 | 171.45 | 2.00 | 1.45 | 4143.07 | 16.89 | 61.49 | 43.84 | 23539.47 | 92.85 | 519.97 | 26.43 | 0.45 | 0.61 | 2.27 | no |
| TRBV7-7_TRBJ2-5 | 0.00 | 0.00 | 0.00 | 0.00 | 0.00 | 0.00 | 0.00 | 0.45 | 0.00 | 1.77 | 0.21 | 0.00 | 0.21 | 0.59 | Inf | no |
| TRBV7-7_TRBJ2-1 | 0.00 | 81.15 | 0.00 | 0.00 | 0.00 | 0.00 | 4.73 | 0.90 | 0.46 | 32.30 | 5.16 | 0.00 | 0.68 | 0.78 | -0.90 | no |
| TRBV7-7_TRBJ1-5 | 0.00 | 0.00 | 0.00 | 0.00 | 0.00 | 0.20 | 0.68 | 0.00 | 0.00 | 0.00 | 1.24 | 0.00 | 0.24 | 0.59 | 3.25 | no |
| TRBV7-7_TRBJ2-4 | 0.00 | 1.31 | 0.00 | 0.00 | 0.00 | 0.00 | 0.00 | 0.45 | 0.00 | 0.00 | 0.00 | 0.00 | 0.56 | 0.67 | -1.53 | no |
| TRBV7-7_TRBJ2-6 | 0.00 | 0.00 | 0.00 | 0.00 | 0.00 | 0.00 | 0.00 | 43.39 | 0.00 | 0.50 | 0.00 | 0.00 | 0.36 | 0.59 | Inf | no |
| TRBV7-7_TRBJ2-2 | 0.00 | 2.62 | 0.00 | 0.00 | 0.00 | 0.00 | 0.00 | 0.00 | 0.00 | 0.50 | 0.21 | 0.00 | 0.50 | 0.64 | -1.88 | no |
| TRBV7-7_TRBJ1-6 | 0.00 | 1.31 | 0.00 | 0.00 | 0.00 | 0.00 | 0.00 | 0.00 | 0.00 | 0.00 | 0.00 | 0.00 | 0.36 | 0.59 | #NAME? | no |
| TRBV7-7_TRBJ2-3 | 0.00 | 2.62 | 0.00 | 0.00 | 0.14 | 0.20 | 1.35 | 0.90 | 0.00 | 1.51 | 0.21 | 0.00 | 0.75 | 0.82 | 0.43 | no |
| TRBV7-7_TRBJ1-2 | 0.00 | 1.31 | 0.00 | 0.00 | 0.00 | 3.62 | 0.00 | 0.00 | 0.00 | 0.50 | 0.00 | 0.30 | 0.31 | 0.59 | -2.61 | no |
| TRBV7-7_TRBJ1-1 | 0.00 | 2.62 | 0.00 | 0.00 | 0.00 | 0.00 | 0.00 | 0.00 | 0.00 | 0.25 | 0.41 | 0.00 | 0.49 | 0.63 | -1.98 | no |
| TRBV7-7_TRBJ2-7 | 0.00 | 2.62 | 0.00 | 0.00 | 0.00 | 0.00 | 0.00 | 0.00 | 0.00 | 3.03 | 1.86 | 0.00 | 0.60 | 0.71 | 0.90 | no |
| TRBV6-5_TRBJ1-5 | 5.80 | 204.17 | 158.29 | 5.92 | 60.50 | 28.15 | 1947.44 | 243.16 | 15747.32 | 651.47 | 2348.64 | 16.71 | 0.23 | 0.59 | 5.50 | no |
| TRBV6-5_TRBJ1-3 | 1.10 | 22.25 | 0.46 | 0.79 | 2.61 | 1705.77 | 9.46 | 858.28 | 42.02 | 645.16 | 56.51 | 3.04 | 0.95 | 0.96 | -0.10 | no |
| TRBV6-5_TRBJ2-5 | 14.27 | 3514.15 | 1463.37 | 0.39 | 79.75 | 314.13 | 64.87 | 4821.09 | 1361.72 | 413.28 | 1865.18 | 15.80 | 0.59 | 0.70 | 0.67 | no |
| TRBV6-5_TRBJ2-1 | 265.26 | 1680.51 | 107470.08 | 28.04 | 5970.60 | 8719.71 | 796.68 | 2235.86 | 536.56 | 7176.72 | 3168.72 | 3575.49 | 0.35 | 0.59 | -2.83 | no |
| TRBV6-5_TRBJ1-6 | 3.14 | 94.23 | 7.38 | 0.53 | 4.40 | 28.15 | 321.65 | 347.56 | 45.71 | 5199.11 | 207.08 | 8.81 | 0.29 | 0.59 | 5.47 | no |
| TRBV6-5_TRBJ2-2 | 41.88 | 7259.97 | 87626.96 | 12.38 | 63.94 | 7208.61 | 1004.80 | 647.21 | 707.41 | 2726.72 | 1454.11 | 108.15 | 0.31 | 0.59 | -3.94 | no |
| TRBV6-5_TRBJ2-4 | 0.00 | 289.25 | 0.00 | 0.00 | 0.96 | 0.60 | 12.84 | 111.63 | 7.39 | 71.66 | 238.64 | 0.30 | 0.69 | 0.78 | 0.61 | no |
| TRBV6-5_TRBJ2-6 | 47.53 | 765.65 | 64466.59 | 16.59 | 654.92 | 136.35 | 320.97 | 775.57 | 247.96 | 4656.14 | 166.86 | 131.54 | 0.39 | 0.59 | -3.39 | no |
| TRBV6-5_TRBJ1-2 | 5.33 | 13546.18 | 1.23 | 0.92 | 115.23 | 53.09 | 379.76 | 1601.31 | 54.49 | 1368.53 | 412.72 | 8.51 | 0.50 | 0.64 | -1.84 | no |
| TRBV6-5_TRBJ2-3 | 8.31 | 280.09 | 851.87 | 2.37 | 55.55 | 215.99 | 68.92 | 3252.33 | 3152.42 | 4973.04 | 1317.77 | 28.56 | 0.07 | 0.59 | 3.18 | no |
| TRBV6-5_TRBJ1-4 | 21.02 | 85.07 | 0.31 | 0.66 | 9.08 | 3382.39 | 101.36 | 1630.23 | 32.78 | 49.96 | 2074.53 | 10.33 | 0.92 | 0.95 | 0.16 | no |
| TRBV6-5_TRBJ2-7 | 29.80 | 9669.49 | 4.15 | 1.71 | 2959.31 | 130.32 | 1119.68 | 986.64 | 1703.42 | 2763.05 | 798.42 | 32.20 | 0.60 | 0.71 | -0.79 | no |
| TRBV6-5_TRBJ1-1 | 20399.99 | 12719.01 | 7.84 | 5.27 | 35.89 | 81.05 | 14991.63 | 1274.08 | 305.22 | 6802.03 | 1110.28 | 1181.70 | 0.78 | 0.84 | -0.37 | no |
| TRBV9_TRBJ2-5 | 119.37 | 31.41 | 0.15 | 0.00 | 1.10 | 37.61 | 2.03 | 2.71 | 1.85 | 6.56 | 2.27 | 0.00 | 0.18 | 0.59 | -3.62 | no |
| TRBV9_TRBJ2-1 | 1.41 | 3.93 | 1.08 | 0.00 | 0.14 | 3.42 | 5.41 | 14.91 | 18.47 | 17.91 | 5.57 | 0.30 | 0.04 | 0.59 | 2.65 | up |
| TRBV9_TRBJ1-3 | 0.00 | 0.00 | 0.00 | 0.00 | 0.28 | 0.20 | 0.00 | 0.90 | 0.00 | 0.25 | 70.75 | 0.00 | 0.36 | 0.59 | 7.24 | no |
| TRBV9_TRBJ1-5 | 0.00 | 0.00 | 0.00 | 0.00 | 0.28 | 0.20 | 0.00 | 0.45 | 0.00 | 0.25 | 2.89 | 0.00 | 0.32 | 0.59 | 2.92 | no |
| TRBV9_TRBJ1-6 | 0.00 | 18.32 | 0.00 | 0.00 | 174.35 | 0.20 | 1.35 | 0.00 | 0.00 | 8.33 | 3.30 | 0.61 | 0.34 | 0.59 | -3.83 | no |
| TRBV9_TRBJ2-2 | 397.50 | 5.24 | 0.00 | 0.26 | 0.28 | 53.49 | 20.27 | 6.78 | 1.85 | 30.28 | 12.38 | 1.52 | 0.37 | 0.59 | -2.64 | no |
| TRBV9_TRBJ2-4 | 0.00 | 0.00 | 0.00 | 0.00 | 0.00 | 0.00 | 0.00 | 2.71 | 0.00 | 6.56 | 0.00 | 0.00 | 0.22 | 0.59 | Inf | no |
| TRBV9_TRBJ2-6 | 0.00 | 0.00 | 0.00 | 0.13 | 0.00 | 0.20 | 0.00 | 5.42 | 0.46 | 0.25 | 0.00 | 0.00 | 0.32 | 0.59 | 4.21 | no |
| TRBV9_TRBJ2-3 | 3.92 | 5.24 | 0.46 | 0.00 | 0.55 | 0.80 | 25.68 | 0.45 | 9.24 | 2.27 | 2.48 | 0.30 | 0.28 | 0.59 | 1.88 | no |
| TRBV9_TRBJ1-2 | 2.67 | 1.31 | 0.00 | 0.39 | 1.24 | 2.41 | 25.68 | 20.34 | 0.00 | 0.25 | 1.03 | 0.61 | 0.23 | 0.59 | 2.58 | no |
| TRBV9_TRBJ1-1 | 0.00 | 39.26 | 0.00 | 0.00 | 0.14 | 0.00 | 0.00 | 0.45 | 1.85 | 1.51 | 1.24 | 0.00 | 0.42 | 0.60 | -2.96 | no |
| TRBV9_TRBJ1-4 | 0.16 | 3.93 | 0.00 | 0.00 | 0.14 | 0.00 | 2.70 | 1.81 | 0.00 | 0.00 | 1.24 | 0.61 | 0.66 | 0.76 | 0.59 | no |
| TRBV9_TRBJ2-7 | 0.94 | 115.18 | 0.15 | 0.00 | 1.10 | 117.24 | 1.35 | 30.28 | 6.93 | 5.05 | 2.68 | 1.52 | 0.26 | 0.59 | -2.30 | no |
