# Supplementary material for: The intrarenal landscape of T cell receptor repertoire in clear cell renal cell cancer
Source: J Transl Med. 2022 Dec 3;20:558. doi: 10.1186/s12967-022-03771-3 (PMC9719196; doi:10.1186/s12967-022-03771-3)
Supplement: Supplementary file 5 — Additional file 5. Specific antigen of ccRCC. [file 12967_2022_3771_MOESM5_ESM.docx]

| Additional file 5. Specific antigen of ccRCC | | | | | | | |
| --- | --- | --- | --- | --- | --- | --- | --- |
| sample_id | db.column.name | db.column.value | unique | frequency | reads | db.unique | weight |
| Convert.A12.TRB | summary | not.found | 2472 | 0.996979843 | 3211627 | 35265 | 50.2078853 |
| Convert.A9.TRB | summary | not.found | 5692 | 0.982442259 | 2093386 | 35265 | 76.27636617 |
| Convert.A11.TRB | summary | not.found | 15435 | 0.97281404 | 4605397 | 35265 | 117.3764259 |
| Convert.A8.TRB | summary | not.found | 7987 | 0.955229919 | 2113237 | 35265 | 85.34816103 |
| Convert.A7.TRB | summary | not.found | 4733 | 0.953344758 | 1394649 | 35265 | 75.07994156 |
| Convert.A10.TRB | summary | not.found | 21366 | 0.938514064 | 3652998 | 35265 | 186.7501046 |
| Convert.A10.TRB | summary | found | 143 | 0.061485936 | 239323 | -1 | 188 |
| Convert.A10.TRB | mhc.class | MHCI | 139 | 0.061483624 | 239314 | 33626 | 139 |
| Convert.A10.TRB | antigen.species | CMV | 86 | 0.05253986 | 204502 | 18688 | 86 |
| Convert.A10.TRB | antigen.epitope | KLGGALQAK | 65 | 0.050125362 | 195104 | 12667 | 65 |
| Convert.A10.TRB | antigen.gene | IE1 | 65 | 0.050125362 | 195104 | 12758 | 65 |
| Convert.A7.TRB | summary | found | 58 | 0.046655242 | 68252 | -1 | 76 |
| Convert.A7.TRB | mhc.class | MHCI | 56 | 0.046653191 | 68249 | 33626 | 56 |
| Convert.A7.TRB | antigen.species | CMV | 32 | 0.046463158 | 67971 | 18688 | 32 |
| Convert.A7.TRB | antigen.epitope | KLGGALQAK | 25 | 0.046411206 | 67895 | 12667 | 25 |
| Convert.A7.TRB | antigen.gene | IE1 | 25 | 0.046411206 | 67895 | 12758 | 25 |
| Convert.A8.TRB | summary | found | 61 | 0.044770081 | 99044 | -1 | 86 |
| Convert.A8.TRB | mhc.class | MHCI | 60 | 0.044768273 | 99040 | 33626 | 60 |
| Convert.A8.TRB | antigen.species | SARS-CoV-2 | 3 | 0.029856063 | 66050 | 2294 | 3 |
| Convert.A8.TRB | antigen.epitope | LLLEWLAMA | 2 | 0.029563152 | 65402 | 13 | 2 |
| Convert.A8.TRB | antigen.gene | ORF14 | 2 | 0.029563152 | 65402 | 13 | 2 |
| Convert.A11.TRB | summary | found | 82 | 0.02718596 | 128701 | -1 | 118 |
| Convert.A11.TRB | mhc.class | MHCI | 80 | 0.02690671 | 127379 | 33626 | 80 |
| Convert.A11.TRB | antigen.species | CMV | 52 | 0.020853814 | 98724 | 18688 | 52 |
| Convert.A11.TRB | antigen.epitope | KLGGALQAK | 43 | 0.019808631 | 93776 | 12667 | 43 |
| Convert.A11.TRB | antigen.gene | IE1 | 43 | 0.019808631 | 93776 | 12758 | 43 |
| Convert.A9.TRB | mhc.class | MHCI | 54 | 0.017557741 | 37412 | 33626 | 54 |
| Convert.A9.TRB | summary | found | 54 | 0.017557741 | 37412 | -1 | 77 |
| Convert.A10.TRB | antigen.gene | pp65 | 27 | 0.016491959 | 64192 | 5641 | 27 |
| Convert.A10.TRB | antigen.epitope | NLVPMVATV | 24 | 0.016489904 | 64184 | 4687 | 24 |
| Convert.A10.TRB | antigen.species | InfluenzaA | 16 | 0.014044063 | 54664 | 4623 | 16 |
| Convert.A10.TRB | antigen.epitope | GILGFVFTL | 13 | 0.014043292 | 54661 | 3501 | 13 |
| Convert.A10.TRB | antigen.gene | M | 13 | 0.014043292 | 54661 | 3501 | 13 |
| Convert.A9.TRB | antigen.species | EBV | 10 | 0.012481709 | 26596 | 4369 | 10 |
| Convert.A9.TRB | antigen.epitope | GLCTLVAML | 6 | 0.01245355 | 26536 | 1039 | 6 |
| Convert.A9.TRB | antigen.gene | BMLF1 | 6 | 0.01245355 | 26536 | 1039 | 6 |
| Convert.A10.TRB | antigen.species | EBV | 29 | 0.011126523 | 43308 | 4369 | 29 |
| Convert.A10.TRB | antigen.species | HomoSapiens | 19 | 0.01060781 | 41289 | 2348 | 19 |
| Convert.A10.TRB | antigen.epitope | RLPGVLPRA | 3 | 0.009308585 | 36232 | 48 | 3 |
| Convert.A10.TRB | antigen.gene | SF3B1 | 3 | 0.009308585 | 36232 | 48 | 3 |
| Convert.A10.TRB | antigen.gene | EBNA4 | 11 | 0.008777796 | 34166 | 2037 | 11 |
| Convert.A10.TRB | antigen.epitope | AVFDRKSDAK | 8 | 0.008773685 | 34150 | 1648 | 8 |
| Convert.A8.TRB | antigen.species | EBV | 13 | 0.007362989 | 16289 | 4369 | 13 |
| Convert.A8.TRB | antigen.epitope | GLCTLVAML | 9 | 0.007329087 | 16214 | 1039 | 9 |
| Convert.A8.TRB | antigen.gene | BMLF1 | 9 | 0.007329087 | 16214 | 1039 | 9 |
| Convert.A9.TRB | antigen.species | CMV | 32 | 0.005021124 | 10699 | 18688 | 32 |
| Convert.A9.TRB | antigen.epitope | KLGGALQAK | 27 | 0.005010799 | 10677 | 12667 | 27 |
| Convert.A9.TRB | antigen.gene | IE1 | 27 | 0.005010799 | 10677 | 12758 | 27 |
| Convert.A11.TRB | antigen.species | HIV-1 | 8 | 0.003953446 | 18716 | 2361 | 8 |
| Convert.A11.TRB | antigen.gene | Nef | 3 | 0.003947743 | 18689 | 328 | 3 |
| Convert.A11.TRB | antigen.gene | Gag | 6 | 0.003946898 | 18685 | 1804 | 6 |
| Convert.A11.TRB | antigen.epitope | KAFSPEVIPMF | 2 | 0.003944996 | 18676 | 246 | 2 |
| Convert.A11.TRB | antigen.epitope | HTQGYFPD | 2 | 0.003944996 | 18676 | 57 | 2 |
| Convert.A11.TRB | antigen.species | HIV | 2 | 0.003944996 | 18676 | 148 | 2 |
| Convert.A8.TRB | antigen.species | HomoSapiens | 7 | 0.003572331 | 7903 | 2348 | 7 |
| Convert.A8.TRB | antigen.epitope | ELAGIGILTV | 6 | 0.003555606 | 7866 | 1422 | 6 |
| Convert.A8.TRB | antigen.gene | MLANA | 6 | 0.003555606 | 7866 | 1457 | 6 |
| Convert.A11.TRB | antigen.species | EBV | 16 | 0.003061618 | 14494 | 4369 | 16 |
| Convert.A12.TRB | mhc.class | MHCI | 39 | 0.003020157 | 9729 | 33626 | 39 |
| Convert.A12.TRB | summary | found | 39 | 0.003020157 | 9729 | -1 | 51 |
| Convert.A12.TRB | antigen.species | CMV | 25 | 0.002908403 | 9369 | 18688 | 25 |
| Convert.A12.TRB | antigen.epitope | KLGGALQAK | 21 | 0.002903125 | 9352 | 12667 | 21 |
| Convert.A12.TRB | antigen.gene | IE1 | 21 | 0.002903125 | 9352 | 12758 | 21 |
| Convert.A8.TRB | antigen.species | CMV | 36 | 0.002505559 | 5543 | 18688 | 36 |
| Convert.A8.TRB | antigen.species | InfluenzaA | 7 | 0.001830238 | 4049 | 4623 | 7 |
| Convert.A8.TRB | antigen.epitope | GILGFVFTL | 6 | 0.001829786 | 4048 | 3501 | 6 |
| Convert.A8.TRB | antigen.gene | M | 6 | 0.001829786 | 4048 | 3501 | 6 |
| Convert.A8.TRB | antigen.epitope | KLGGALQAK | 27 | 0.001796336 | 3974 | 12667 | 27 |
| Convert.A8.TRB | antigen.gene | IE1 | 27 | 0.001796336 | 3974 | 12758 | 27 |
| Convert.A8.TRB | antigen.gene | pp65 | 13 | 0.001783227 | 3945 | 5641 | 13 |
| Convert.A8.TRB | antigen.epitope | NLVPMVATV | 11 | 0.001775995 | 3929 | 4687 | 11 |
| Convert.A11.TRB | antigen.gene | EBNA4 | 5 | 0.001599037 | 7570 | 2037 | 5 |
| Convert.A11.TRB | antigen.epitope | IVTDFSVIK | 1 | 0.001589321 | 7524 | 551 | 1 |
| Convert.A11.TRB | antigen.epitope | GLCTLVAML | 8 | 0.001460468 | 6914 | 1039 | 8 |
| Convert.A11.TRB | antigen.gene | BMLF1 | 8 | 0.001460468 | 6914 | 1039 | 8 |
| Convert.A11.TRB | antigen.epitope | RAKFKQLL | 6 | 0.001446527 | 6848 | 838 | 6 |
| Convert.A11.TRB | antigen.gene | BZLF1 | 6 | 0.001446527 | 6848 | 873 | 6 |
| Convert.A10.TRB | antigen.epitope | RAKFKQLL | 5 | 0.001426141 | 5551 | 838 | 5 |
| Convert.A10.TRB | antigen.gene | BZLF1 | 5 | 0.001426141 | 5551 | 873 | 5 |
| Convert.A10.TRB | antigen.epitope | FLYALALLL | 1 | 0.001422801 | 5538 | 28 | 1 |
| Convert.A10.TRB | antigen.gene | LMP2A | 1 | 0.001422801 | 5538 | 48 | 1 |
| Convert.A10.TRB | antigen.epitope | ELAGIGILTV | 12 | 0.001296142 | 5045 | 1422 | 12 |
| Convert.A10.TRB | antigen.gene | MLANA | 12 | 0.001296142 | 5045 | 1457 | 12 |
| Convert.A11.TRB | antigen.gene | pp65 | 13 | 0.001061871 | 5027 | 5641 | 13 |
