# Supplementary figures and images for: The intrarenal landscape of T cell receptor repertoire in clear cell renal cell cancer
Source: J Transl Med. 2022 Dec 3;20:558. doi: 10.1186/s12967-022-03771-3 (PMC9719196; doi:10.1186/s12967-022-03771-3)

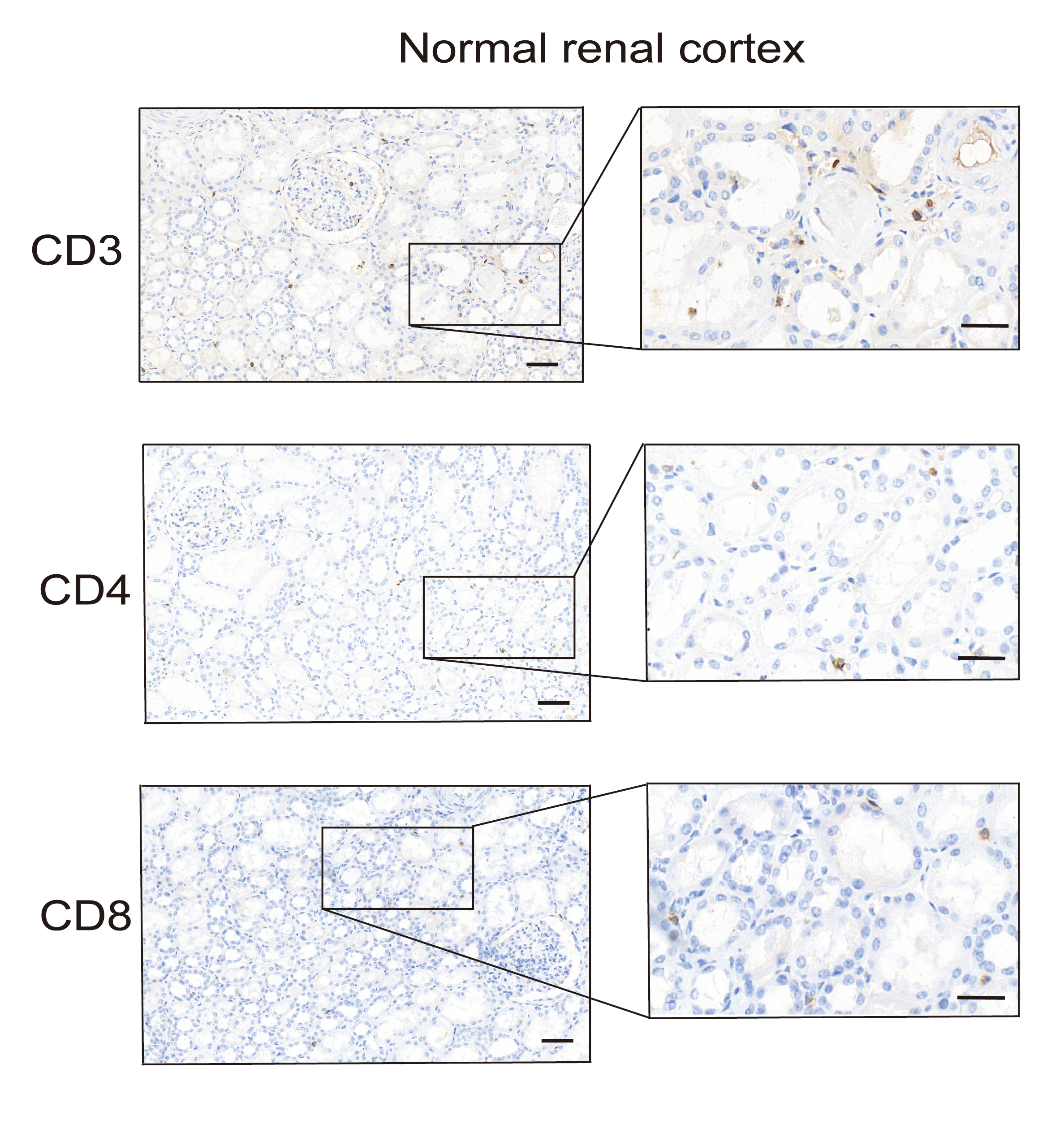

Supplement: Supplementary file 6 — Additional file 6: Figure S1. Representative images of CD3, CD4, and CD8 immunohistochemistry results in normal renal cortex. Scale bar, 100 μm. [file 12967_2022_3771_MOESM6_ESM.jpg]

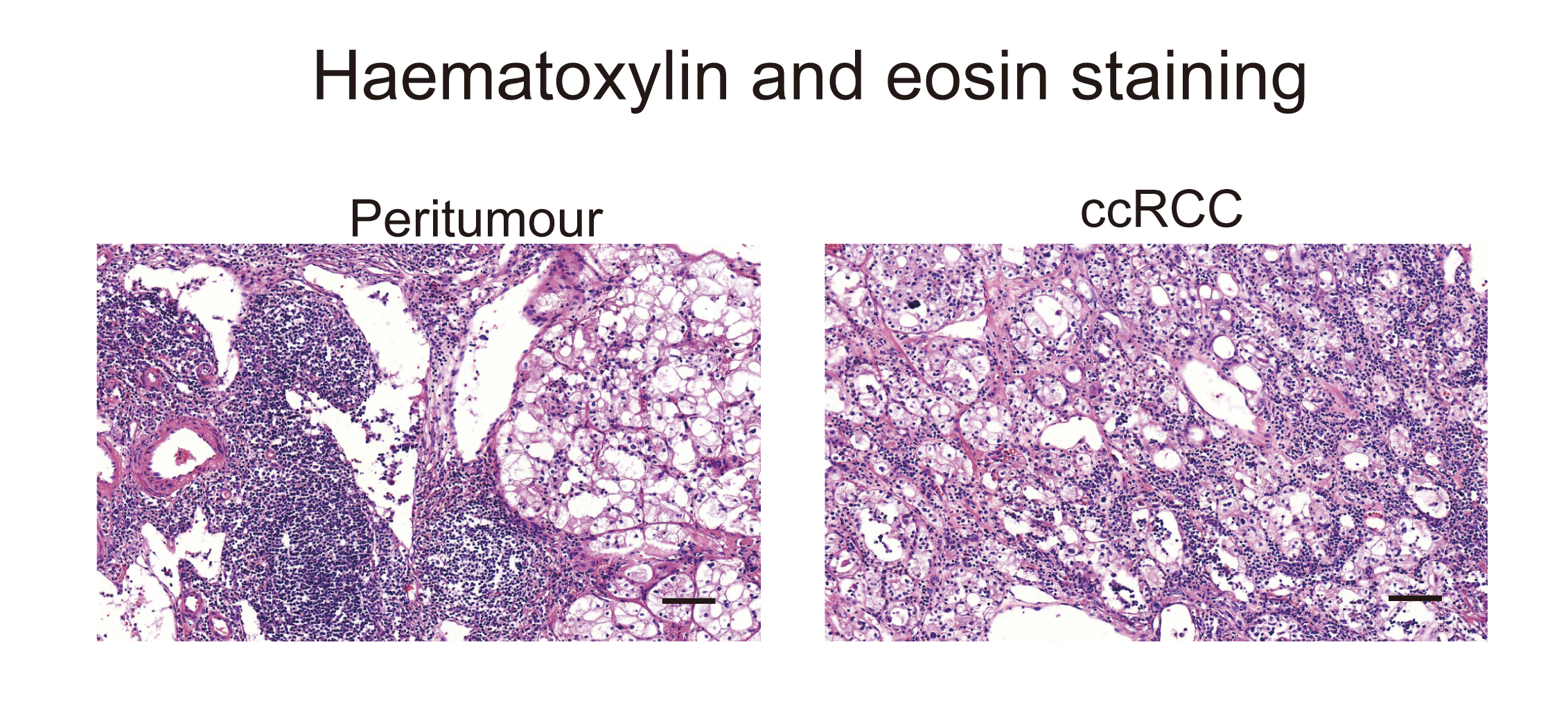

Supplement: Supplementary file 7 — Additional file 7: Figure S2. Representative images of haematoxylin and eosin staining in peritumour (Left) and ccRCC (Right) tissue. Scale bar, 100 μm. [file 12967_2022_3771_MOESM7_ESM.jpg]

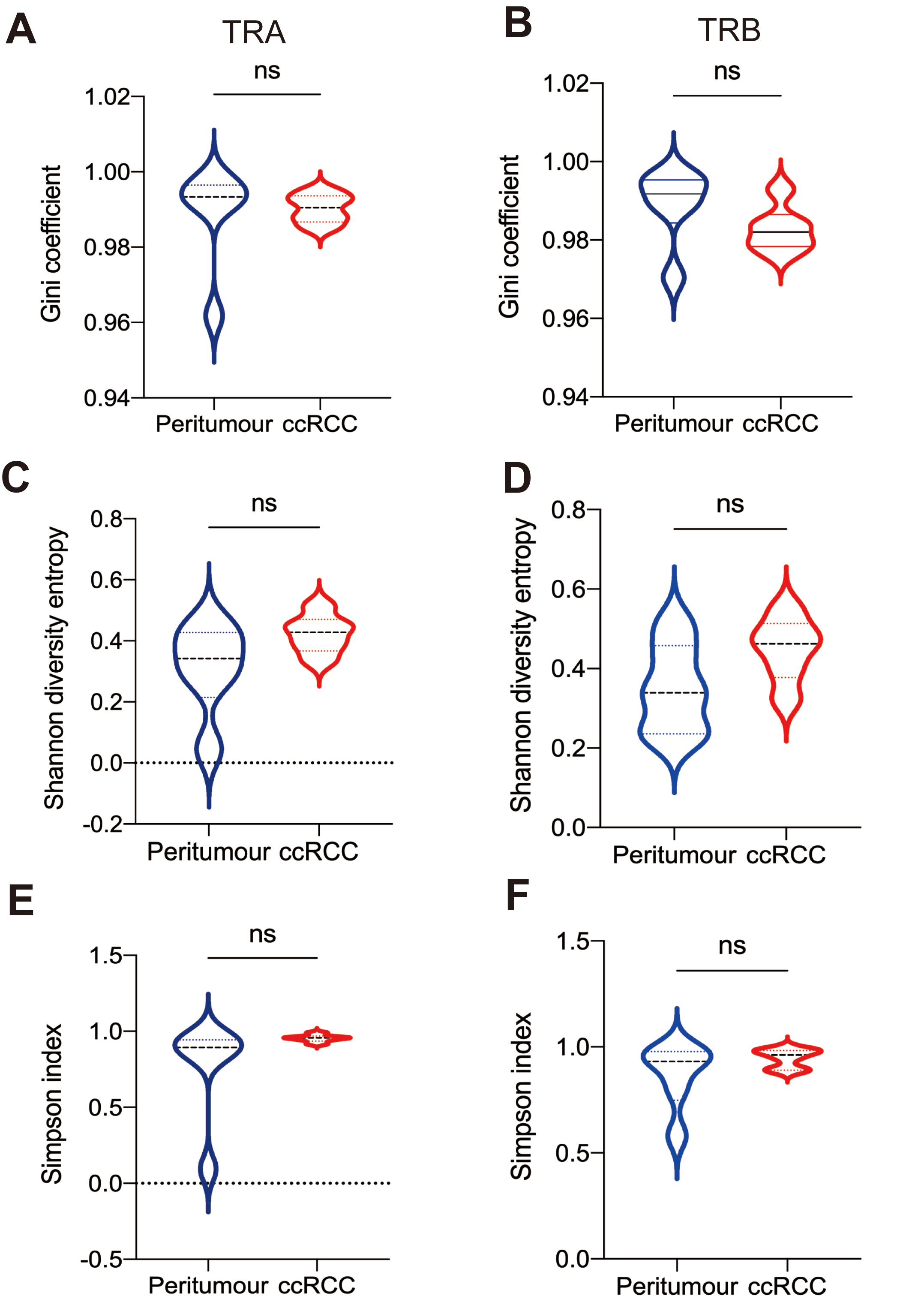

Supplement: Supplementary file 8 — Additional file 8: Figure S3. Comparison of the peritumor and ccRCC tissue TCR repertoire diversity by Gini coefficient (A, B), Shannon diversity entropy (C, D), and Simpson index (E, F). Data are mean ± SEM. Student’s t-test is used to calculate the Gini coefficient, Shannon diversity entropy, and Simpson index difference in the ccRCC and peritumour groups. P value >0.05; *P value <0.05; **P value <0.01; ***P value <0.001; ns, not significant. [file 12967_2022_3771_MOESM8_ESM.jpg]
