# Supplementary material for: The intrarenal landscape of T cell receptor repertoire in clear cell renal cell cancer
Source: J Transl Med. 2022 Dec 3;20:558. doi: 10.1186/s12967-022-03771-3 (PMC9719196; doi:10.1186/s12967-022-03771-3)
Supplement: Supplementary file 9 — Additional file 9: Table S1. Analysis of antigen in Peritumour tissue. [file 12967_2022_3771_MOESM9_ESM.docx]

| Table S1. Analysis of antigen in Peritumour tissue. | | | | |
| --- | --- | --- | --- | --- |
|  | TRA | | TRB | |
| Antigen name | Antigen value | Frequency (%) | Antigen value | Frequency (%) |
| MHC. class | MHCI | 4.40 | MHCI | 1.06 |
| Antigen. species | CMV | 3.12 | CMV | 1.06 |
|  | EBV | 1.47 | SARS-CoV-2 | 1.06 |
|  | HIV-1 | 0.87 |  |  |
|  | HomoSapiens | 0.70 |  |  |
|  | InfluenzaA | 0.51 |  |  |
|  | HCV | 0.41 |  |  |
|  | SARS-CoV-2 | 0.32 |  |  |
| Antigen. gene | IE1 | 3.01 | IE1 | 1.06 |
|  | EBNA3A | 1.23 | Nucleocapsid | 1.06 |
|  | BZLF1 | 1.04 |  |  |
|  | EBNA4 | 0.94 |  |  |
|  | Gag | 0.87 |  |  |
|  | BST2 | 0.70 |  |  |
|  | M | 0.51 |  |  |
|  | CORE | 0.37 |  |  |
|  | pp65 | 0.25 |  |  |
|  | Matrix | 0.24 |  |  |
|  | Spike | 0.08 | KLGGALQAK | 1.06 |
|  | NS3 | 0.04 | SPRWYFYYL | 1.06 |
| Antigen. epitope | KLGGALQAK | 3.01 |  |  |
|  | RLRAEAQVK | 1.23 |  |  |
|  | RAKFKQLL | 1.04 |  |  |
|  | AVFDRKSDAK | 0.94 |  |  |
|  | IVTDFSVIK | 0.87 |  |  |
|  | RTLNAWVKV | 0.79 |  |  |
|  | LLLGIGILV | 0.70 |  |  |
|  | GILGFVFTL | 0.51 |  |  |
|  | GPRLGVRAT | 0.37 |  |  |
|  | NLVPMVATV | 0.25 |  |  |
